# Single-cell and spatial profiling highlights TB-induced myofibroblasts as drivers of lung pathology

**DOI:** 10.1084/jem.20251067

**Published:** 2026-01-05

**Authors:** Ian M. Mbano, Nuo Liu, Marc H. Wadsworth, Mark J. Chambers, Thabo Mpotje, Osaretin E. Asowata, Sarah K. Nyquist, Kievershen Nargan, Duran Ramsuran, Farina Karim, Travis K. Hughes, Joshua D. Bromley, Robert Krause, Threnesan Naidoo, Liku B. Tezera, Michaela T. Reichmann, Sharie Keanne Ganchua, Henrik N. Kløverpris, Kaylesh J. Dullabh, Rajhmun Madansein, Sergio Triana, Adrie J.C. Steyn, Bonnie Berger, Mohlopheni J. Marakalala, Gabriele Pollara, Sarah M. Fortune, JoAnne L. Flynn, Paul T. Elkington, Alex K. Shalek, Alasdair Leslie

**Affiliations:** 1 Africa Health Research Institute, Durban, South Africa; 2 School of Laboratory Medicine and Medical Sciences, College of Health Sciences, University of KwaZulu-Natal, Durban, South Africa; 3 Institute for Medical Engineering & Science, Department of Chemistry, and Koch Institute for Integrative Cancer Research, Massachusetts Institute of Technology, Cambridge, MA, USA; 4 Ragon Institute of MGH, MIT, and Harvard, Cambridge, MA, USA; 5 Broad Institute of MIT and Harvard, Cambridge, MA, USA; 6 Program in Computational & Systems Biology, Massachusetts Institute of Technology, Cambridge, MA, USA; 7 Computer Science & Artificial Intelligence Lab, Massachusetts Institute of Technology, Cambridge, MA, USA; 8 Program in Health Sciences & Technology, Harvard Medical School and Massachusetts Institute of Technology, Boston, MA, USA; 9 Program in Immunology, Harvard Medical School, Boston, MA, USA; 10 Microbiology Graduate Program, Massachusetts Institute of Technology, Cambridge, MA, USA; 11 NIHR Biomedical Research Centre, School of Clinical and Experimental Sciences, Faculty of Medicine, University of Southampton, Southampton, UK; 12 Institute for Life Sciences, University of Southampton, Southampton, UK; 13 Department of Infection and Immunity, University College London, UK; 14 Department of Immunology and Microbiology, University of Copenhagen, Copenhagen, Denmark; 15 Department of Microbiology, Centre for AIDS Research and Free Radical Biology, University of Alabama at Birmingham, Birmingham, AL, USA; 16 Department of Mathematics, Massachusetts Institute of Technology, Cambridge, MA, USA; 17 Department of Microbiology and Molecular Genetics, University of Pittsburgh School of Medicine, Pittsburgh, PA, USA; 18 Center for Vaccine Research, University of Pittsburgh, Pittsburgh, PA, USA; 19 Department of Immunology and Infectious Diseases, Harvard T.H. Chan School of Public Health, Boston, MA, USA

## Abstract

Tuberculosis (TB) typically causes lung destruction and fibrosis, leading to ∼1.3 million deaths annually. The cellular drivers of human TB immunopathology remain poorly defined. We performed single-cell RNA sequencing and spatial transcriptomics on lung tissues from TB-infected and TB-negative individuals, identifying 30 distinct immune, parenchymal, and stromal cell subsets. Several were linked to TB pathology and corroborated through immunohistochemistry, flow cytometry, and independent human datasets. Fibroblasts were identified as major drivers in both active TB granuloma and TB-diseased lung tissue. In particular, the *MMP1*^+^*CXCL5*^+^ fibroblast subset, expressing a myofibroblast-like gene signature, was associated with severe disease and higher bacterial burden in nonhuman primate granulomas. Network analyses revealed cross talk between *MMP1*^+^*CXCL5*^+^ fibroblasts and *SPP1*^+^ macrophages within the granuloma cuff, which has been reported in other disease contexts, and may play an important role in TB immunopathology. Our findings highlight previously unappreciated cell populations and potential targets for novel TB therapies.

## Teaser

Single-cell and spatial transcriptomics of human TB lung tissues from individuals in South Africa revealed that MMP1^+^CXCL5^+^ fibroblasts and SPP1^+^ macrophages are linked to TB disease and TB lung granuloma, uncovering targetable cellular cross talk underlying TB immunopathology and potential avenues for host-directed therapies.

## Introduction

Tuberculosis (TB), caused by infection with *Mycobacterium tuberculosis* (*M.tb*), remains a global epidemic, with ∼10.6 million new cases and 1.3 million deaths annually (Carranza et al., 2020, World Health Organization, 2023). The development of highly effective anti-TB drugs and programmatic improvements led to global cure rates of ∼85% in drug-susceptible TB from 1995 to 2015, as well as reduced mortality rates (World Health Organization, 2020). Unfortunately, however, mortality remains persistently high (Pai et al., 2022), highlighting the need for improved interventions.

*M.tb* infection occurs primarily in the lung, where interactions between host cells and the pathogen typically result in the formation of a granuloma—an aggregation of infected myeloid cells, usually surrounded by an inner ring of macrophages and an outer cuff of lymphoid cells. This specialized immunological niche is highly heterogeneous in its overall cellular makeup, with the composition of each lesion independently influencing bacterial growth and disease progression (Davis and Ramakrishnan, 2009; McCaffrey et al., 2022). In progressive TB, extensive lung extracellular matrix (ECM) remodeling via both matrix destruction and fibrosis leads to the formation of lung cavities that facilitate transmission (Ravimohan et al., 2018). This ECM remodeling also increases the risk of post-TB lung disease (PTLD), resulting in high rates of recurrent TB infection and mortality—even after successful eradication of initial infection (Allwood et al., 2021; Romanowski et al., 2019). Although some features of the immunopathology of TB infection that lead to PTLD are known, including granuloma formation, cytokine production, hypoxia-inducible factors, and production of matrix metalloproteinases (MMPs), the exact mechanisms remain unclear (Allwood et al., 2021). While animal models of TB disease—from zebrafish to nonhuman primates (NHPs)—have provided valuable insights into aspects of these processes, they do not fully recapitulate human pathology (Fonseca et al., 2017). Critically, these models generally reflect primary infection, often without the cavities and the extensive ECM remodeling observed in human TB, and fail to capture the development of chronic secondary TB disease that arises in humans (Hunter, 2018). Consequently, the key features and cellular drivers of immunopathology in human TB remain poorly understood.

The advent of high-throughput single-cell RNA sequencing (scRNA-seq) has transformed our ability to analyze the cellular makeup of complex tissues and phenotypic changes associated with disease (Cui et al., 2019). For example, application of this technology to study idiopathic pulmonary fibrosis (IPF)—a lung disease characterized by dysregulated ECM turnover—identified aberrant basal-like cells, peribronchiolar endothelial cells, *SPP1*^*+*^ macrophages, and myofibroblasts as key drivers of pulmonary tissue remodeling, suggesting new strategies to combat the disease (Adams et al., 2020). Similar characterization of the cell types and states involved in the immunopathogenesis of human TB and PTLD could potentially help uncover effective targets for host-directed therapies (HDTs) (Hawn et al., 2013).

Here, we applied scRNA-seq and spatial transcriptomics to human TB-diseased lung tissues and TB-negative controls to examine the cellular and molecular features of TB lung disease and investigate mediators of immunopathology. Overall, we identified depletion of most macrophage subsets and an enrichment of fibroblast and neutrophil subsets in TB-diseased lungs, consistent with altered fibrotic and pro-inflammatory activity. We validated these observations with bulk RNA-seq data of LN TB granuloma from a well-characterized cohort of treatment-naïve, culture-confirmed TB patients (Reichmann et al., 2021). To further contextualize specific disease-associated cell subsets, we integrated our data with those from the Human Lung Cell Atlas (HLCA) and NHP lung TB granulomas (Gideon et al., 2022; Sikkema et al., 2023). This enabled us to uncover a putative central role for fibroblast subsets—including a MMP1^+^CXCL5^+^ fibroblast cluster expressing a myofibroblast-like gene module—in TB immunopathology, where we further evidenced via flow cytometry and immunohistochemistry. Through cell network analyses on the single-cell data, we found that these cells appear to coordinate their activities with macrophages, including an *SPP1*^*+*^ subset not previously implicated in TB biology that was observed to be coresident in immunohistochemical stainings of human lung TB granuloma. Moreover, these two subsets were co-inducible by a standard skin challenge of TB patients with *M.tb*-derived antigen (tuberculin), and analyses of spatial transcriptomics data from an independent cohort of TB patients showed colocalization of both this myofibroblast signature and the *SPP1*^*+*^ macrophage signal within lung TB granuloma cuffs. Overall, our data reveal key cellular subsets and pathways that could inform next-generation HDTs and provide an essential reference for the community.

## Results

### Cellular composition of human TB-infected lung tissue

**Figure 1.**
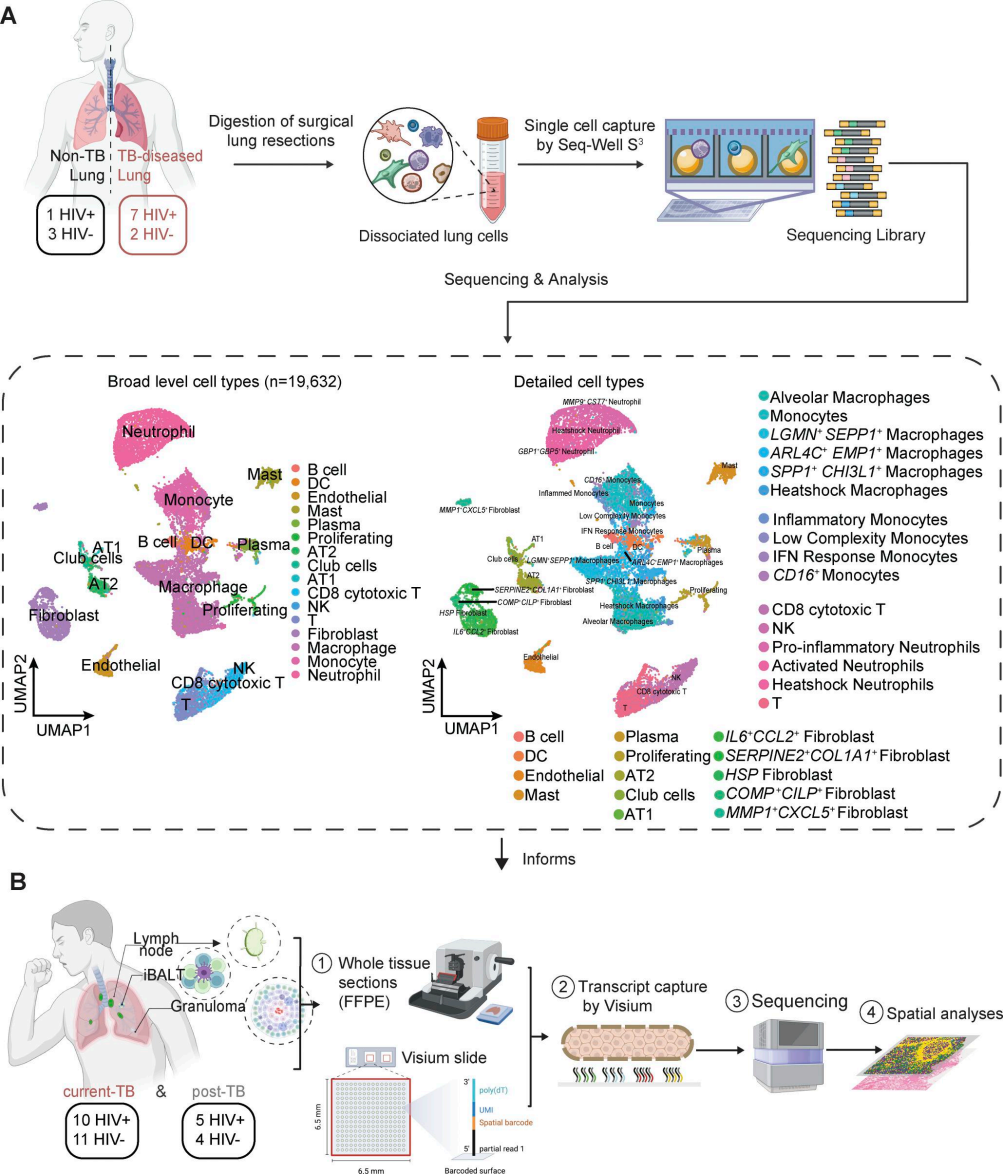
**Overview of the single-cell and spatial data generated from TB-diseased and control lungs. (A)** Schematic showing the experimental flow for the isolation of cells from human lung tissues, generation of single-cell libraries using Seq-Well S^3^. Four TB-negative and nine TB-positive lung samples were processed through scRNA-seq. Shown adjacent to the process flow is a low-dimensional embedding (UMAP) of the 19,632 cells passing quality control annotated with high-level cell types (middle) or detailed cell subtype (right). **(B)** 10x Visium platform workflow for spatial transcriptomics profiling on FFPE samples from TB-diseased lung resections. 21 of these samples come from current TB patients with detectable *M.tb*; 9 came from post-TB patient, where bacteria are no longer detected in BAL TB culture after infection. Samples contain either granulomas, iBALTs, or lung LNs, representing different pathological states.

**Figure S1.**
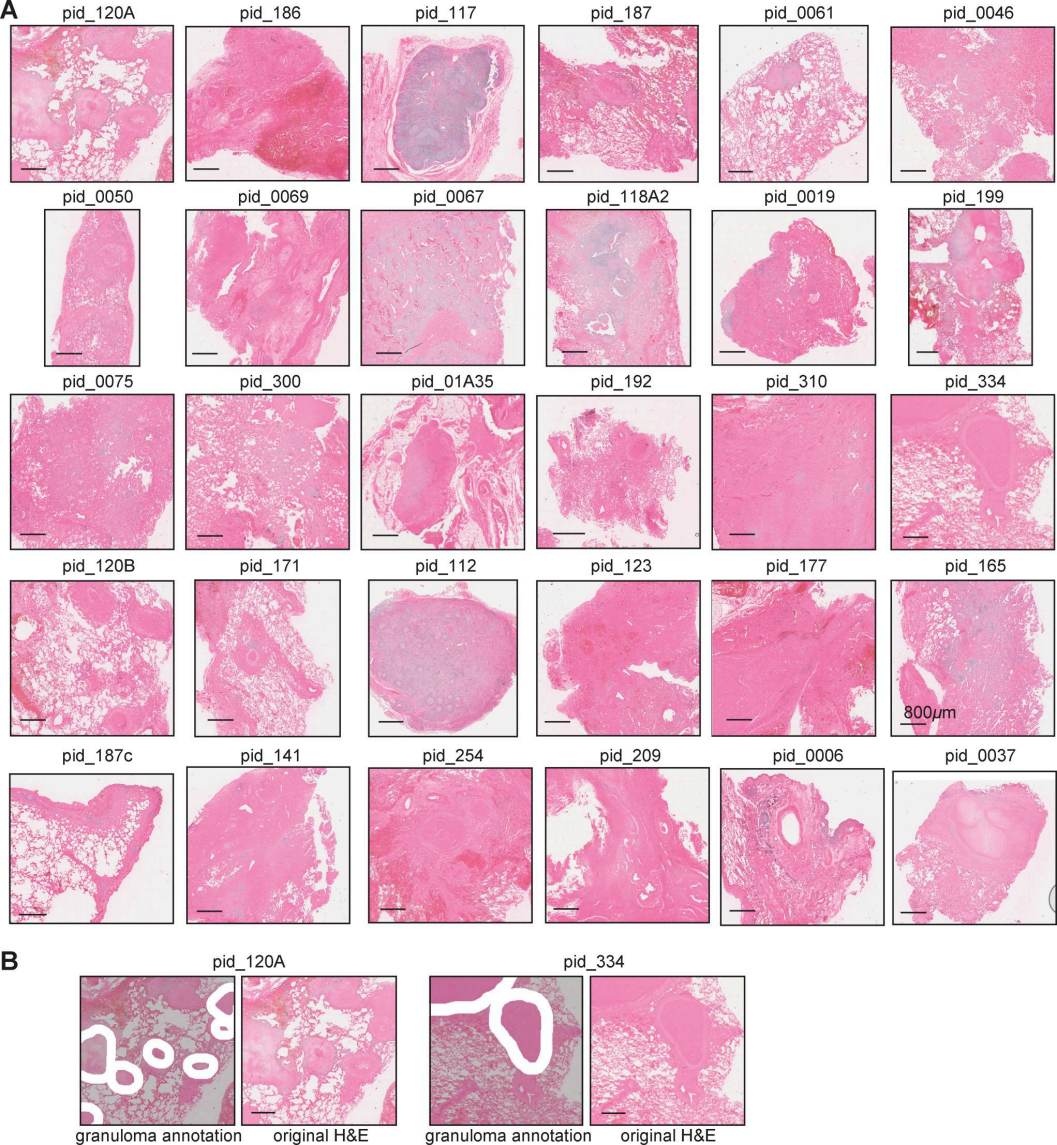
**Spatial transcriptomics on TB-infected human lung samples and single-cell deconvolution. (A)** H&E staining on all 30 lung samples from patients previously infected with TB. Scale bars: 800 μm. Identical images for pid_0037, pid_177, pid_0186, pid_187, pid_0192, pid_199, pid_0209, and pid_304. **(B)** Examples of manual annotation on granuloma structures on H&E staining images. Scale bars: 800 μm.

Fresh, TB-diseased human lung tissue pieces were obtained from nine participants (seven HIV^+^ TB; two HIV^−^ TB) enrolled in the African Health Research Institute lung cohort study (Fig. 1 A). All participants underwent TB treatment after initial diagnosis but had subsequent lung resection surgery to treat complications consistent with PTLD, including hemoptysis and bronchiectasis (Table S1). As a control, TB-negative lung samples were obtained from the healthy tissue margins of four surgically resected lung tumors (one HIV^+^ cancer control; three HIV^−^ cancer controls). All participants, irrespective of TB status, received prophylactic anti-TB treatment prior to surgery. Tissue pieces were washed thoroughly and homogenized into a single-cell suspension via mechanical and enzymatic digestion using an optimized protocol in BSL3 containment (Ardain et al., 2019). Lung cells were then processed and sequenced following the Seq-Well S^3^ protocol as described previously to obtain our scRNA-seq dataset (Hughes et al., 2020). An additional 30 samples were obtained from different participants in the same TB lung cohort and profiled using the 10x Visium Spatial Gene Expression platform (Fig. 1 B and Data S11). As above, all participants received TB-drug treatment prior to surgery. Fresh tissue pieces were removed from resected lung tissue and preserved using standard formalin-fixed paraffin-embedded (FFPE) procedures, followed by Visium version 2 chemistry protocols with paired H&E staining to generate reference images (Fig. S1 A; Materials and methods). 21 samples (10 HIV^+^, 11 HIV^−^) were derived from subjects with active microbiologically confirmed TB, termed “current TB.” The remaining samples (five HIV^+^, four HIV^−^) termed “post-TB,” were obtained from individuals in whom bacterial load was no longer detectable from bronchoalveolar lavage (BAL) TB culture. This spatial dataset contains both samples with TB lung granulomas and samples with inducible bronchus-associated lymphoid tissues (iBALTs) or lung-draining LNs, which are considered as less severe pathological states. For each granuloma sample, pathological grading and manual annotation of the granuloma structures on the H&E image were performed by an expert TB pathologist to enable better disease contextualization (Fig. S1 B and Data S7).

**Figure S2.**
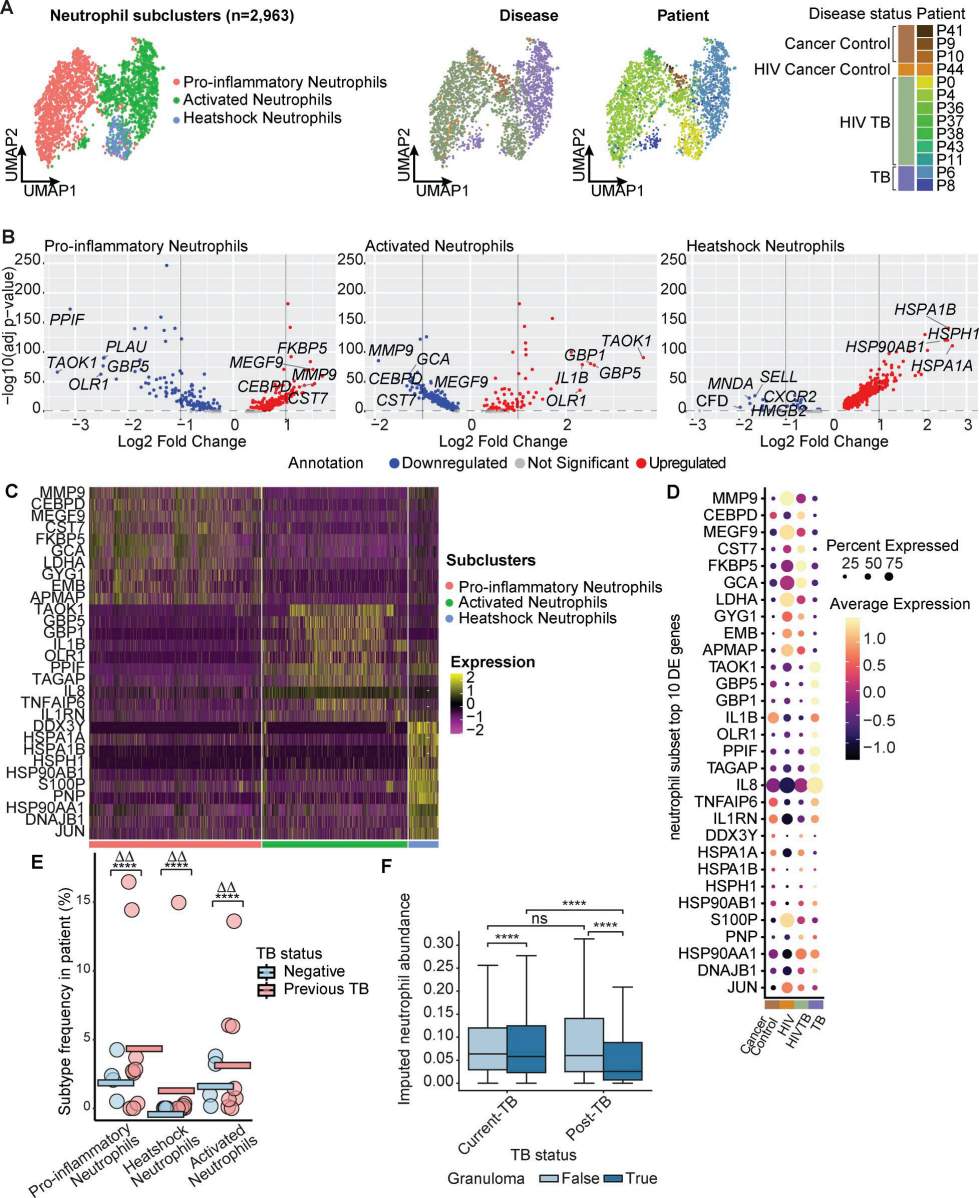
**Single-cell transcriptomic reveals heterogeneity within neutrophil populations with disease-specific difference. (A)** Neutrophil (*n* = 2,963) subclustering reveals three subclusters (left), also colored by patient ID (middle) and disease condition (right). **(B)** Volcano plot of differential gene expression results of each neutrophil subcluster compared with the rest. Y axis shows −log10 (BH-adjusted P value); x axis shows log2 fold change between cells in subcluster and outside the subcluster. **(C)** Heatmap of subtype top 10 differentially expressed (DE) genes in each of the neutrophil subcluster. **(D)** Expression of marker genes in neutrophil subclusters by disease conditions. **(E)** Fisher’s exact test on abundance of detailed neutrophil subclusters between TB conditions. Statistical annotations: fold-change >2 (ΔΔ). **(F)** Cell2loc imputed neutrophil abundance distribution on the Visium dataset grouped by TB and granuloma status (Materials and methods). The 5% quantile of the estimated posterior distribution of cell abundance at each Visium spot is displayed, representing the value of cell abundance that the model has high confidence in. Two-sided Mann–Whitney U test without correction were used for statistical testing. ****: P < 0.0001.

**Figure 2.**
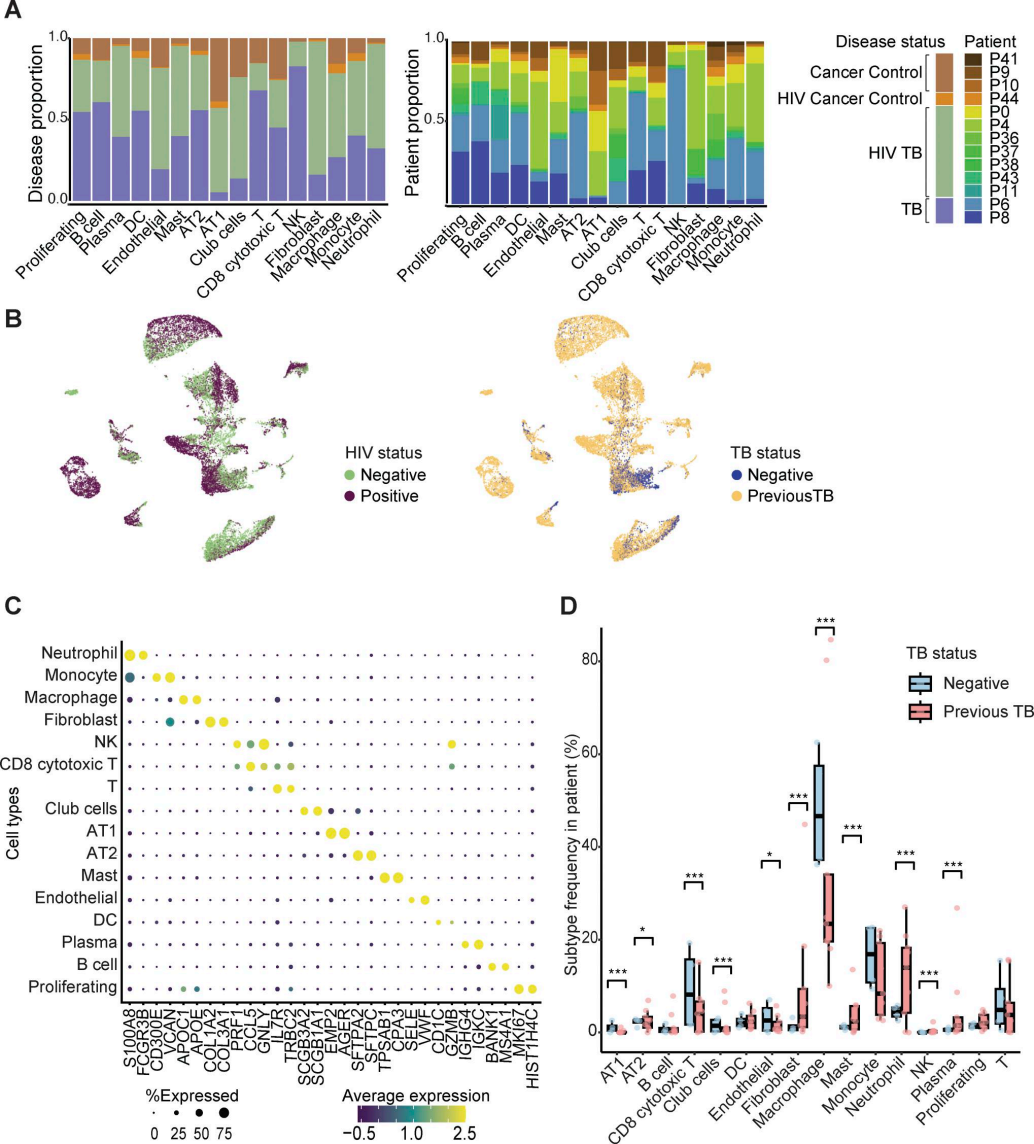
**Overview of tissue heterogeneity and cell type abundance in the single-cell dataset. (A)** Cell type proportions by disease status (left) and patient (right, *n* = 7 HIV^+^TB^+^; *n* = 2 TB^+^; *n* = 1 HIV^+^, *n* = 3 cancer control). **(B)** Low-dimensional embedding (UMAP) of all scRNA-seq data colored by patient HIV status (left) and TB status (right). **(C)** Dot plot showing expression levels of top 2 DE genes in each of the broad-level cell types. **(D)** Two-sided Fisher’s exact test for abundance of major cell types between samples from patients with previous TB diagnosis and samples from control patients. Holm’s method was applied to adjust P values for multiple-testing correction. Statistical annotations: P value < 0.05 (*) and P value < 0.001 (***).

After quality control of the scRNA-seq data, we retained 19,632 high-quality single-cell profiles from the homogenized lung tissues. Neighborhood-based clustering revealed 16 canonical cell types. Further subclustering of high abundance populations resulted in a total of 30 phenotypically distinct immune, parenchymal, and stromal subsets (Fig. 1 A; Data S1, A–E; Fig. S2, A–F; and Data S2, A and B; Materials and methods). The fractional representation of cells per participant and clinical characteristic varied between clusters reflecting biological heterogeneity between patients, TB disease states, and potentially anatomical sampling location, though our data are limited with respect to the latter (Fig. 2 A and Table S1). Most cells derived from HIV^−^ TB samples, and while most of the clusters contained cells from the majority of patients, we observed substantial inter-patient variability in cell numbers (Fig. 2 B and Table S2). Canonical cell type markers and genes differentially expressed between clusters were examined for manual annotation (Fig. 2 C; Materials and methods). Notably, we found large populations of neutrophils, which are captured by Seq-Well S^3^ but often underrepresented by other scRNA-seq technologies due to their fragility (Hughes et al., 2020). Overall, observed clusters closely mirrored those seen in a scRNA-seq characterization of lung tissue from IPF patients and healthy donors (Reyfman et al., 2019).

Next, we looked for evidence of differential abundance by comparing the representation of each cell type per donor between the TB-diseased and TB-negative lung groups, irrespective of HIV status (Fig. 2 D). Given the limitation in cohort size, we were underpowered to detect significant cell type proportion differences at the sample level through a Wilcoxon test or z-proportion test (P > 0.05 for patient level comparisons). However, at the single-cell level, pronounced shifts in the frequency of most cell types were observed between the TB-diseased and TB-negative groups, including an expansion of neutrophils in the TB-diseased group, consistent with several human studies linking neutrophil recruitment with TB lung pathology (Leisching, 2018; Muefong and Sutherland, 2020; Sutherland et al., 2009). We also found an increased frequency of mast and plasma B cells in TB-diseased tissue, supporting findings from recent scRNA-seq studies of NHP models where both cell populations were expanded in TB granuloma with higher bacterial burden (Gideon et al., 2022). In addition to these immune cell populations, fibroblasts were enriched in TB-diseased lung tissue. Conversely, in TB-diseased lung samples, we found a decrease in the proportions of macrophages, the cell type targeted by and primarily responsible for killing bacilli, and CD8 T cells, thought to contribute to *M.tb* control (Queval et al., 2017; Winchell et al., 2023). Although we detected significant changes between the abundance of these cell subsets using a Fisher’s exact test, given high inter-patient variability and limited sample numbers, we were underpowered to determine significance using a Dirichlet-multinomial regression or a Wilcoxon test (Smillie et al., 2019).

### Specific innate immune cell subclusters are associated with TB-diseased human lung tissue

Given the limited participant numbers that compose our scRNA-seq dataset, we leveraged these data to impute cell type abundances in both the current- and post-TB lung samples in our spatial transcriptomics cohort. This allowed us to better understand the phenotypic shifts associated with TB disease and select relevant single-cell subclusters for further characterization. This strategy drove us to focus on neutrophils, macrophages, monocytes, and fibroblasts, whose abundances also showed the most dramatic shifts between the TB and control samples in the scRNA-seq data (Fig 2 D).

#### Neutrophil subclusters

Neutrophils play a crucial role in the innate immune system and are quickly recruited as a first line of defense against bacterial infections. They are suggested to have immunoregulatory functions in TB granulomas in NHP models (Gideon et al., 2019); however, their role in the immunopathogenesis of human TB has been contentious and less well understood (Gaffney et al., 2022).

Neutrophils were highly enriched in TB-diseased lung tissue in our single-cell dataset (Fig. 2 D) and associated with three distinct subclusters (termed “pro-inflammatory neutrophils,” activated neutrophils,” and heat-shock [HSP] neutrophils”). Both activated neutrophil and pro-inflammatory neutrophil subclusters expressed markers genes associated with IFN-γ and TNF-α signaling—critical responses linked to inflammation and immune activation in TB disease (Chandra et al., 2022; Luster et al., 2005; Pagán and Ramakrishnan, 2018) (Fig. S2, A–D; and Data S8). Activated neutrophils were annotated by their high expression of neutrophil activation markers, including *IL1RN,* and *IL1B* and *IL8,* inflammatory cytokines involved in neutrophil recruitment (Fig. S2, B–D) (de Oliveira et al., 2013; Prince et al., 2004). They also expressed GBP1 and GBP5, genes involved in a previously described blood neutrophil transcriptional signature used to diagnose pulmonary TB (Zak et al., 2016). Pro-inflammatory neutrophils, in contrast, highly expressed high levels of MMP9, CST7, and LDHA (Fig. S2, B–D). MMP9 is a proteinase involved in the degradation of ECM that is strongly associated with TB granuloma (Rohlwink et al., 2019); CST7 (cystatin F) is a neutrophil marker of acute inflammation (Huang et al., 2021); and, LDHA encodes lactate dehydrogenase, which enhances neutrophil migration and activity, and is highly elevated in hypoxic lung TB granuloma in animals (Chowdhury et al., 2022; Krishnamoorthy et al., 2020). Pro-inflammatory neutrophils also highly expressed FKBP5 and CEBPD, both implicated in an immunometabolic network predictive of TB progression (Duffy et al., 2019), and VEGFA, PLAUR, TPM4, and CD44, which are involved in neutrophil recruitment and lymphangiogenesis during inflammation (Adams et al., 2021; He et al., 2024; Tan et al., 2013; Zhou et al., 2021) (Data S8). The remaining small subcluster of neutrophils, marked by high expression of heat-shock protein genes (HSP neutrophils), was also elevated in TB-diseased lungs, which is notable given that heat-shock protein expression by neutrophils can trigger pro-inflammatory response in macrophages (Kauffman et al., 2018; Zheng et al., 2004) (Fig. S2, A–E).

Given the small sample size and high HIV prevalence in our scRNA-seq dataset, we examined neutrophils in the spatial cohort to understand the link between neutrophils and TB disease, running cell type deconvolution using the scRNA-seq cohort as reference and imputing individual cell type abundances (Materials and methods). Within granuloma structures, neutrophil abundance was significantly higher in sample from current TB infections than those from post-TB infections, consistent with the recruitment of this cell type to the granuloma during active disease (Fig. S2 F). Interestingly, however, neutrophils were more abundant in non-granuloma tissues (e.g., iBALT and LN) compared with granuloma, though the difference was less pronounced in current TB. This may reflect the involvement of neutrophils in tissue remodeling and chronic inflammation associated with both active TB and PTLD (Santos et al., 2025).

To further test the association between neutrophil subsets and TB disease, we quantified the expression of each subset’s top marker genes in an independent bulk RNA-seq dataset generated from laser-captured human LN TB granuloma, in which all patients were HIV negative (Materials and methods; Data availability) (Reichmann et al., 2021). Importantly, these LN were excised prior to TB therapy initiation and contained viable *M.tb* bacilli. We found that 7 of the top 10 unique marker genes associated with the activated neutrophils were significantly upregulated in LN granuloma compared with noninfected LN controls (Data S9), as well as the pro-inflammatory markers *CEBPD* and *LDHA* (Data S9). We note that although LNs are common sites of extrapulmonary TB, LN granulomas have functional and structural differences from those found in the lung, which may contribute to differences in expression levels of these marker genes (Ganchua et al., 2020; Mekonnen et al., 2021).

#### Monocyte and macrophage subclusters

Macrophages are necessary to control TB disease but also provide a niche for bacterial growth and survival (Guirado et al., 2013). In addition, they have been implicated in pulmonary remodeling, with reported roles in both promoting and inhibiting pathology (Kishore and Petrek, 2021). Tissue-infiltrating monocytes, meanwhile, provide a source for macrophage differentiation and are key players in inflammatory response and bacterial persistence (Sampath et al., 2018). Hence, understanding the functional differences in monocytes and macrophages between the TB-diseased and TB-negative controls could provide insights into understanding TB immunopathology. In aggregate, macrophages were significantly decreased in TB-diseased lung tissue, and monocytes were decreased, albeit not significantly (Fig. 2 D).

**Figure 3.**
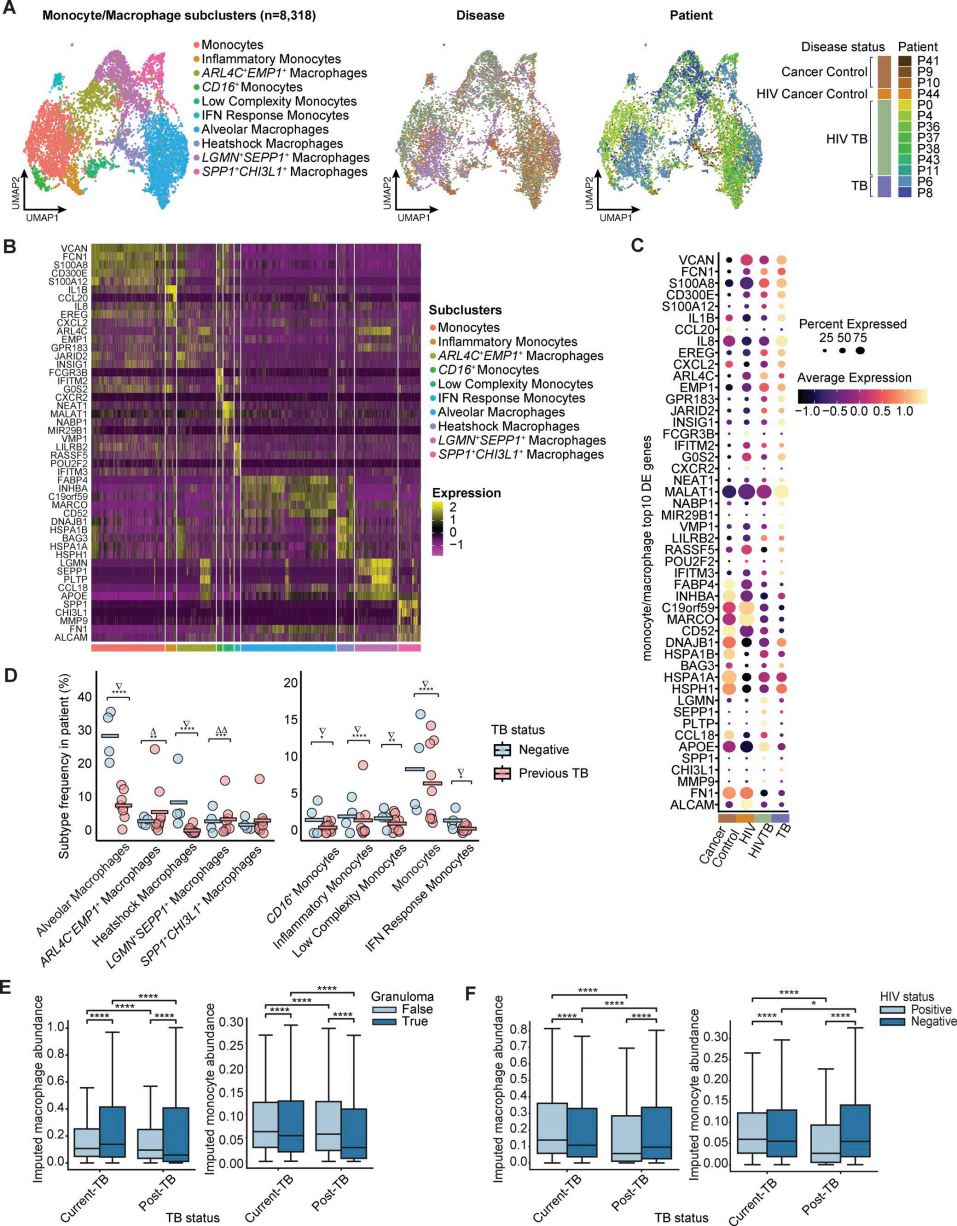
**Single-cell transcriptomic reveals heterogeneity within monocyte and macrophage populations with disease-specific difference. (A)** Monocyte/macrophage (*n* = 8,318) subclustering reveals 10 subclusters (left), also colored by patient ID (middle) and disease condition (right). **(B)** Heatmap of subtype top 10 DE genes in each of the monocyte/macrophage subcluster. **(C)** Expression of marker genes in monocyte/macrophage subclusters by disease conditions. **(D)** Two-sided Fisher’s exact test on abundance of detailed macrophage (left) and monocyte (right) subclusters between TB conditions. Holm’s method was applied to adjust P values for multiple-testing correction. Statistical annotations: P value < 0.05 (*), P value < 0.01 (**), P value < 0.001 (***), fold-change >1 (Δ), fold-change >2 (ΔΔ), and fold-change <1 (∇). **(E)** Cell2loc imputed macrophage (left) and monocyte (right) abundance distribution on the Visium dataset grouped by TB and granuloma status (Materials and methods). The 5% quantile of the estimated posterior distribution of cell abundance at each Visium spot is displayed, representing the value of cell abundance that the model has high confidence in. Two-sided Mann–Whitney U test without correction were used for statistical testing. Statistical annotations: P value < 0.0001 (****). **(F)** Similar to E, but grouped by TB status and HIV status.

Subclustering of 8,313 macrophages/monocytes single-cell transcriptomes generated 10 distinct subclusters, which we annotated manually based on marker genes (Fig. 3, A–C and Data S2 A). Alveolar macrophages (*INHBA*^+^*FABP4*^*+*^*MARCO*^+^) and a subcluster we termed “heat-shock (HSP) macrophages” were significantly reduced in TB-diseased lung tissue compared with TB-negative lungs. The former may reflect the loss of normal lung alveolar structure observed in TB-diseased lung tissue. Upregulated proteins in *HSP* macrophages included those encoding for Hsp70 family proteins (e.g., *HSPA1A, HSPA1B, HSPA6*, and *HSPA8*), which are known to modulate NF-κB–mediated release of pro-inflammatory cytokines from alveolar macrophages in pulmonary TB (Radons, 2016; Wang et al., 2017). In addition, when tested separately, all monocyte subsets were significantly reduced in TB disease, possibly due to rapid transition to macrophage phenotypes in the pro-inflammatory environment of the diseased lung (Desalegn and Pabst, 2019). The remaining three macrophage subsets (defined by *ARL4C/EMP1*, *LGMN/SEPP1*, and *SPP1/CHI3L1*) were higher in TB-diseased lung, but subtly so (Fig. 3 D). Therefore, to explore the potential skewing of macrophage subsets further, we performed cluster-free differential abundance testing using Milo, which models cellular states as overlapping neighborhoods on k-nearest neighbor graphs representing the similarities between single-cell profiles (Dann et al., 2022). This analysis highlighted the underrepresentation of alveolar macrophages in TB-diseased lung tissue, as this was the only subcluster with its phenotypic neighborhoods depleted among TB disease samples (Data S2, C and D). In contrast, although present at low frequency, *ARL4C*^*+*^*EMP1*^*+*^, *LGMN*^*+*^*SEPP1*^+^, and *SPP1*^*+*^*CHI3L1*^+^ macrophages were all significantly associated with TB-diseased lung. Marker genes enriched in *ARL4C*^*+*^*EMP1*^*+*^ macrophages did not obviously associate with published functional annotations but included *GPR138*, which favors *M.tb* replication in macrophages (Tang et al., 2020). *LGMN*^*+*^*SEPP1*^+^ macrophages were enriched for lipid metabolism activities, while *SPP1* encodes for osteopontin, a known macrophage attractant, which has been associated with granulomatous diseases and is upregulated in *M.tb* infection (Nau et al., 1997; Wang et al., 2020) (Data S8).

In our spatial cohort, we observed a higher abundance of both macrophages and monocytes in current TB compared with post-TB, consistent with continuous recruitment of myeloid cells during active disease (Fig. 3 E, left, and Fig. 3 F, left). As with neutrophils, macrophages were more abundant in granuloma from current TB samples compared with post-TB. Additionally, in current TB, macrophages were more abundant in granuloma compared with non-granuloma tissue, while the opposite was true for monocytes, which may be explained by maturation into macrophages within this environment. (Fig. 3 E, right). HIV co-infection was associated with increases in macrophages in current TB samples and a decrease in monocyte abundance in both current and post-TB samples, suggesting a potential effect of HIV co-infection on myeloid populations during both active TB and PTLD (Fig. 3 F). In active TB samples, HIV infection may lead to more macrophages to compensate for the loss of CD4^+^ T cells, which are important for adaptive immune responses against *M.tb* (Bromley et al., 2023, *Preprint*). The decrease in macrophages associated with HIV co-infection in post-TB samples, however, might reflect impaired monocyte differentiation or persistent depletion of macrophage precursors (Burdo et al., 2013; Campbell et al., 2014).

**Figure S3.**
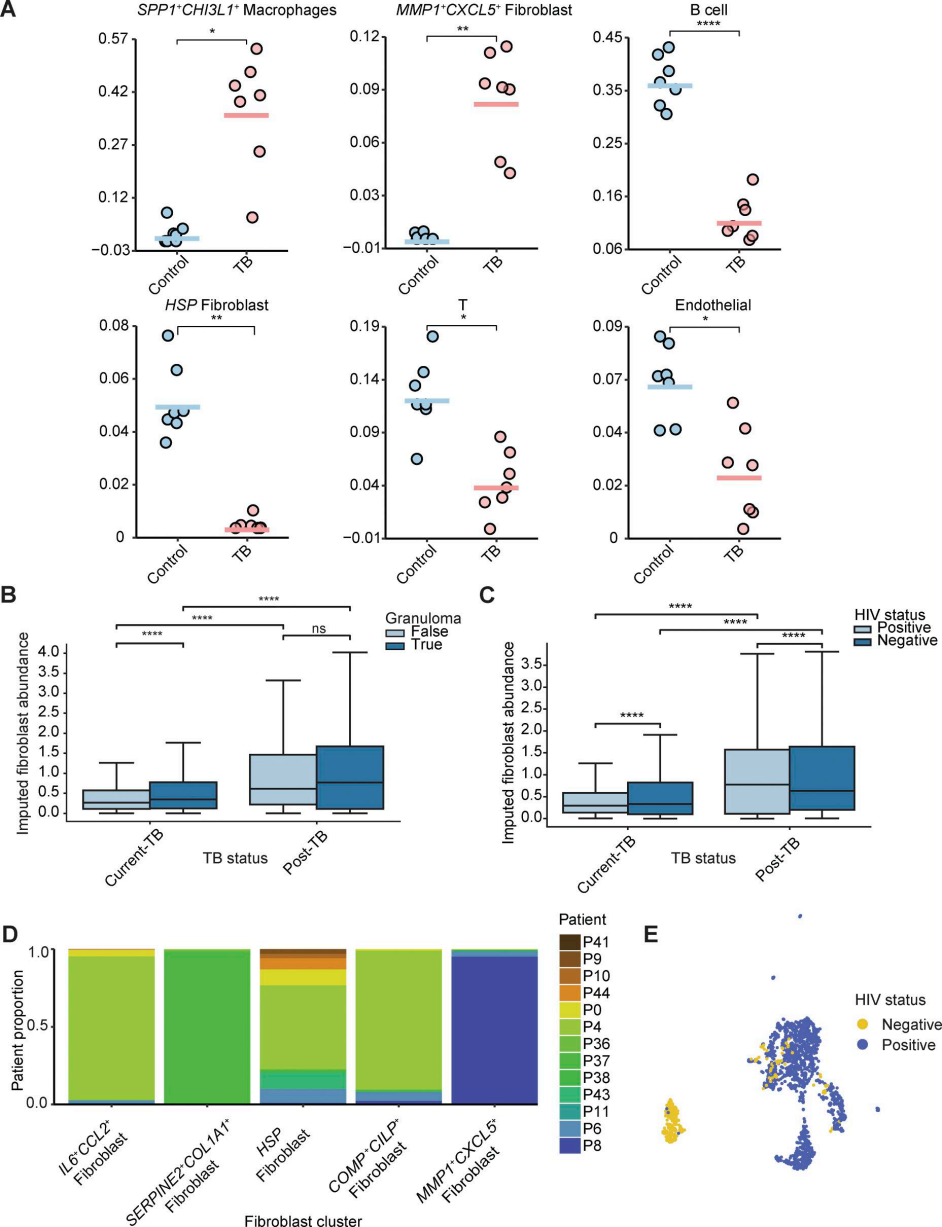
**Deconvolution of bulk human LN dataset and fibroblast in spatial and single-cell dataset. (A)** Dot plot showing distribution of cell type proportion from deconvolution results on each bulk RNA-seq human LN TB granuloma sample, separated by cell type and colored by TB conditions. Only cell types with significant difference between TB conditions are shown. Two-sided *T* test with Bonferroni correction was used to compare the means. Statistical annotations: P value < 0.05 (*) and P value < 0.01 (**). **(B)** Cell2loc imputed fibroblast abundance distribution on the Visium dataset group by TB and granuloma status (Materials and methods). The 5% quantile of the estimated posterior distribution of cell abundance per Visium spot is displayed, representing the value of cell abundance that the model has high confidence in. Two-sided Mann–Whitney U test without correction were used for statistical testing. P value < 0.0001 (****); P value > 0.05 (ns). **(C)** Same as B, but grouped by HIV and TB status. **(D)** Bar plot of patient distribution in each fibroblast subcluster. **(E)** UMAP embedding of fibroblasts colored by HIV status of the sample.

To further contextualize monocyte/macrophage subclusters in human TB granuloma, we similarly assessed expression of subcluster marker genes in the LN dataset described above. We found the strongest signal for the *SPP1*^*+*^*CHI3L1*^*+*^ macrophages, where 5/10 of the top markers of the subcluster were significantly upregulated in human LN TB, over fivefold in the case of *SPP1* and *FN1* (Data S9). Bulk gene expression deconvolution of this data supported a significant increase in the frequency of several populations in untreated TB granuloma compared with control LN, including the *SPP1*^*+*^*CHI3L1*^*+*^ macrophage (Fig. S3 A).

Finally, to investigate these myeloid subsets under control conditions, we examined the expression of subcluster defining genes within single-cell data from TB-granuloma isolated from experimentally infected NHPs (Gideon et al., 2022). In this dataset, we identified overlapping gene signatures between most of the subclusters observed in our current study, including the *SPP1*^*+*^*CHI3L1*^*+*^ macrophage population (*LILRB4, MMP9, PKM, MYOF,* and *CHI3L1*) (Data S10) (Gideon et al., 2022). Collectively, these data identify diverse myeloid subsets in TB-diseased lung tissue and support a putative role for *SPP1*^*+*^*CHI3L1*^*+*^ macrophages in TB immunopathology in humans and NHPs.

### Single-cell analysis identifies TB-associated fibroblast populations

**Figure 4.**
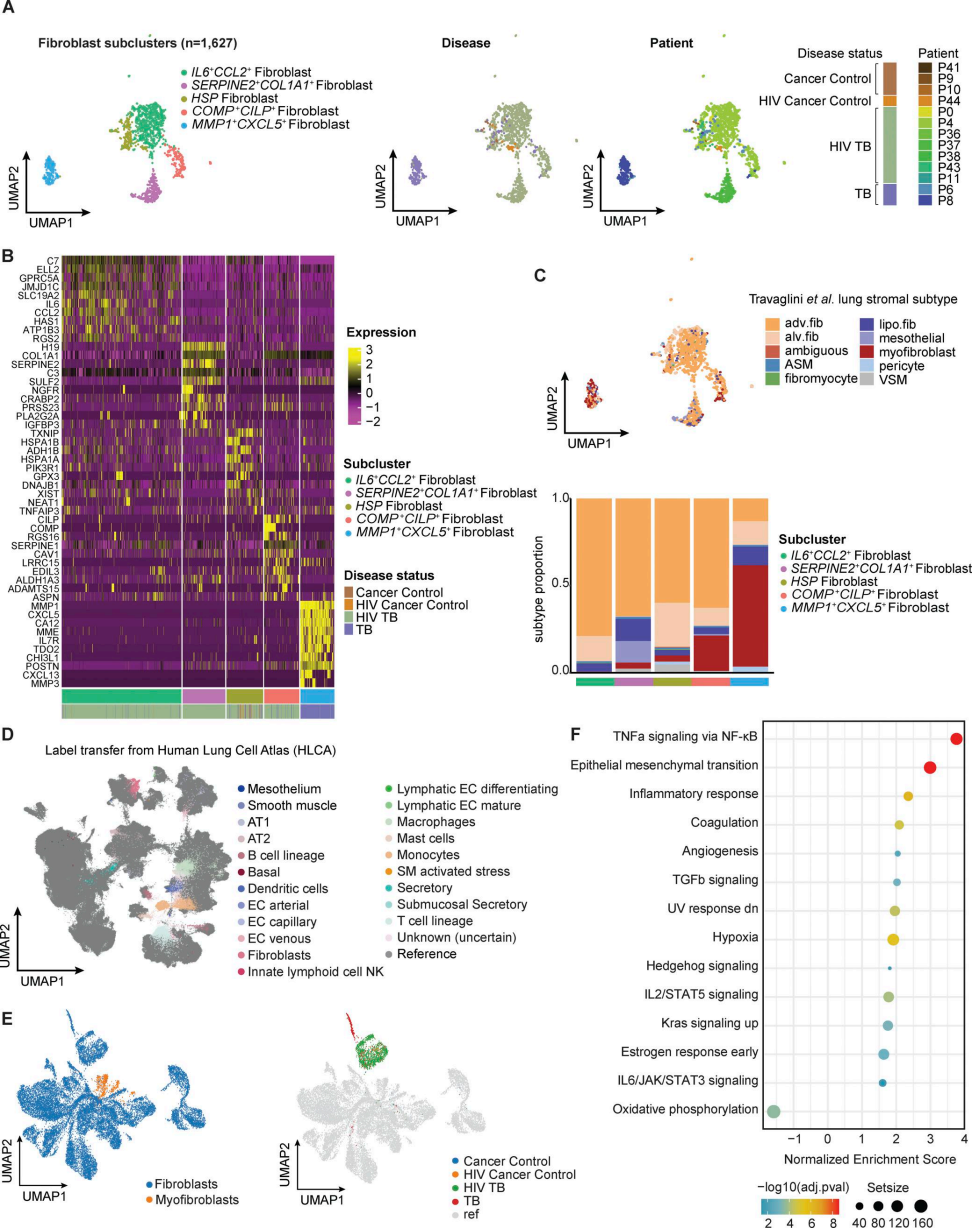
**Fibroblast exhibit TB-specific phenotypes. (A)** Fibroblast (*n* = 1,627) subclustering reveals five subclusters (left), also colored by patient ID (middle) and disease condition (right). **(B)** Heatmap of subtype top 10 DE genes in each of the fibroblast subcluster. Columns (cells) are annotated by fibroblast subclusters and sample source disease status. **(C)** Comparing annotation against literature stromal annotation from Travaglini et al. (2020). Left: Original fibroblast UMAP as seen in A colored by mapped cell types in Travaglini et al. (2020). Right: Barplot showing distributions of mapped cell type in each original subcluster. ASM, airway smooth muscle; VSM, vascular smooth muscle; MyoF, myofibroblast; FibM, fibromyocyte; AdvF, adventitial fibroblast; AlvF, alveolar fibroblast; LipF, lipofibroblast; Peri, pericyte; Meso, mesothelial. **(D)** Reference mapping to the HLCA. Query (all cells in this study, *n* = 19,632) vs. reference cells (*n* = 584,944) on integrated UMAP with transferred label from HLCA to query cells. **(E)** Query (all fibroblasts in this study that was mapped to fibroblast/myofibroblast in label transfer, *n* = 1,601) and reference lung fibroblast cells (*n* = 17,500) from HLCA colored by annotation (either “Fibroblast” or “Myofibroblast”) and TB conditions. **(F)** GSEA on DE genes between TB fibroblasts and TB-negative fibroblasts on HLCA-integrated data.

**Figure S4.**
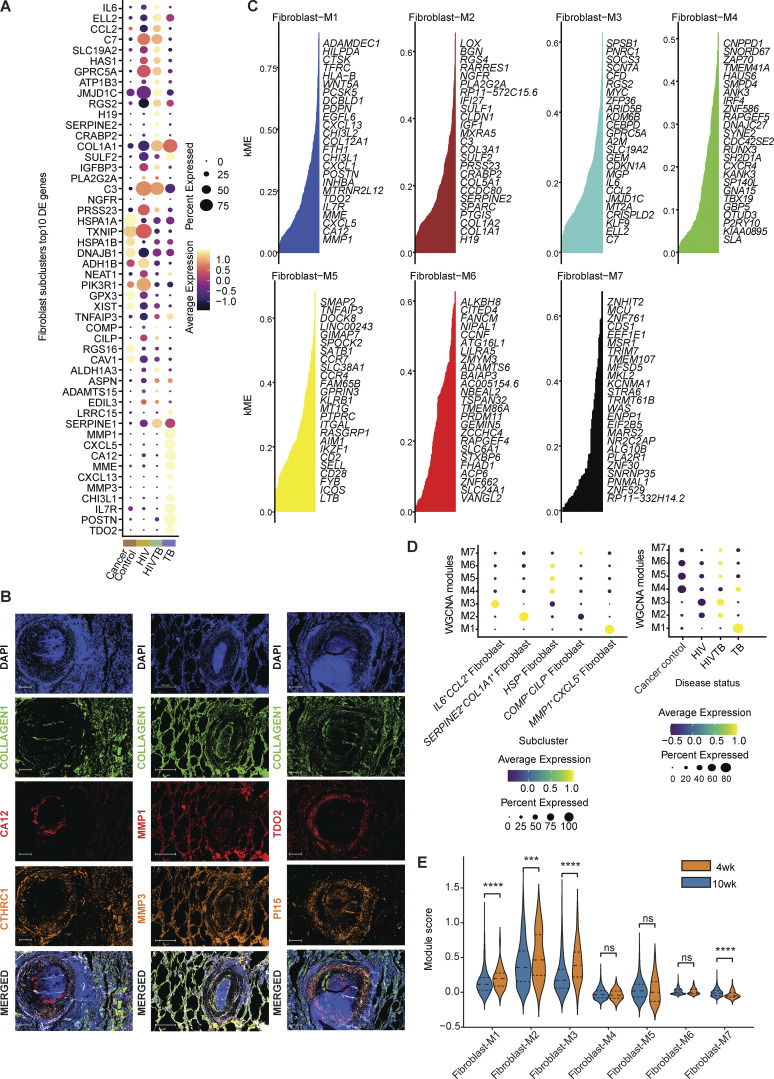
**Fibroblast subclusters’ marker genes, WGCNA analysis, and comparisons against public datasets. (A)** Expression of marker genes in fibroblast subclusters by disease conditions. **(B)** Fluorescence immunohistochemistry images of human TB granuloma showing nuclear staining (DAPI), protein expression of COL1A1, and the *MMP1^+^CXCL5^+^* fibroblast subcluster-specific genes MMP1, MMP3, TDO2, and PI15. Scale bars: 200 μm (left), 500 μm (middle), and 200 μm (right). **(C)** Top 25 hub genes by eigen-based connectivity (kME) in each fibroblast WGCNA module. Correlation between each module based on their MEs, a metric representing the expression of each module in each cell. **(D)** WGCNA module expression across fibroblast subclusters and HIV status in TB samples. **(E)** Evaluation of all WGCNA module expression in NHP TB granuloma fibroblasts from samples gathered at 10 wk compared with 4 wk. Two-sided Mann–Whitney U test without correction was used on each module. Statistical annotations: P value < 0.001 (***) and P value < 0.0001 (****).

Despite playing a prominent role in tissue remodeling in other lung diseases, there is limited understanding of how fibroblasts contribute to granuloma formation, immunopathology, and protective TB immunity (Lee et al., 2019; McCaffrey et al., 2022). In our spatial transcriptomics samples, fibroblast abundance was estimated to be higher in granuloma samples than iBALT/LN samples for both TB conditions, suggesting fibroblast involvement in long-term tissue remodeling and granuloma formation (Fig. S3 B). Holding granuloma status or HIV status constant, we observed a higher abundance of fibroblasts in post-TB samples relative to current TB samples, consistent with the role fibroblasts play in long-term tissue damage in PTLD (Fig. S3, B and C). Further subclustering of the 1,627 fibroblasts in the scRNA-seq dataset revealed five distinct subclusters (Fig. 4 A and Fig. S3, D and E): *IL6*^*+*^*CCL2*^*+*^ fibroblasts, *SERPINE2*^*+*^*COL1A1*^*+*^ fibroblasts, heat-shock (HSP) fibroblasts, *COMP*^*+*^*CILP*^*+*^ fibroblasts, and *MMP1*^*+*^*CXCL5*^*+*^ fibroblasts (Fig. 4 B and Fig. S4 A). We note that most of the fibroblasts we recovered came from TB-diseased patients, a trend consistent with fibrotic change due to TB damage in the lung (Gai et al., 2023). One of the subclusters, MMP1^+^CXCL5^+^ fibroblasts, almost solely consists of cells from HIV^-^ TB patients, whereas the others were mostly occupied by cells from HIV^+^ TB patients, suggesting potentially different phenotypes for fibroblasts in HIV/TB co-infected patients versus patients with TB alone (Fig. 4 A and Fig. S3 E). This subcluster also had the strongest phenotypic shifts among fibroblast populations in a Milo analysis (Data S2, C and D).

To better understand the phenotypic properties of these five subclusters, we further contextualized them against the existing literature by mapping them onto a trained reference model for lung stromal cell annotation (Travaglini et al., 2020) (Fig. 4 C). The majority mapped strongly to adventitial fibroblasts, which are associated with pulmonary vascular remodeling in response to stress, including hypoxia and infection (Stenmark et al., 2011). Although canonically associated with vascular beds, adventitial fibroblasts become highly migratory and invasive in response to activating signals, notably including osteopontin (SPP1), and have been shown to drive tissue remodeling by inducing a pro-inflammatory/profibrotic phenotype in macrophages through IL-6 signaling, (Anwar et al., 2012; El Kasmi et al., 2014). The MMP1^+^CXCL5^+^ fibroblast cluster, however, mapped primarily to the myofibroblast phenotype, followed by the lipofibroblast phenotype. Myofibroblasts are involved in wound healing after tissue injury and can differentiate from recruited fibroblasts under mechanical stress, through the influence of cytokines like TGF-β, and epithelial-to-mesenchymal transition (EMT) (Li et al., 2016; Talbott et al., 2022). In addition, lipofibroblasts can differentiate into myofibroblasts during fibrosis (El Agha et al., 2017). Consistent with this, overrepresentation analysis (ORA) showed that the *MMP1*^*+*^*CXCL5*^*+*^ fibroblast markers were enriched among genes associated with EMT and myoblast differentiation (Data S8).

To test the association between *MMP1*^*+*^*CXCL5*^*+*^ fibroblast markers and TB, we again examined the LN dataset and found that 5/10 top unique marker genes in the *MMP1*^*+*^*CXCL5*^*+*^ fibroblast subcluster were upregulated in the LN TB data, including *MMP1, CA12, TDO2, POSTN,* and *COL12A1* (Data S9). Interestingly, *CA12* plays a role in many biological processes, including preventing calcification (Zhao et al., 2020), an essential process in granuloma resolution (Lin et al., 2014). In addition, this gene was found to co-express with MMP1 and CXCL5 in a subset of cancer-associated fibroblasts associated with poor clinical outcome (Qin et al., 2023). We also observed a significant increase in the imputed frequency of *MMP1*^*+*^*CXCL5*^*+*^ fibroblasts in TB LN granuloma compared with control LNs via deconvolution of bulk RNA-seq profiles (Fig. S3 A). Together, these data suggest TB is associated with skewing of lung fibroblasts to phenotypes that overlap with known disease processes in the infected lung.

To confirm the presence of the *MMP1*^*+*^*CXCL5*^*+*^ phenotype via an orthogonal method, we stained sections of human lung from the same surgical cohort that contained distinct TB granuloma (5 µm sections from two patients) for associated gene products of the *MMP1*^*+*^*CXCL5*^*+*^ subcluster: COL1, TDO2, MMP1, MMP3, and CA12, together with PI-15 and CTHRC-1, which were also significantly upregulated in this subcluster (Fig. S4 B and Data S8). COL1, a general fibroblast marker, was expressed across lung tissue; the MMPs, which are secreted to facilitate ECM breakdown, had less strict localization; TDO2, CA12, PI-15, and CTHRC-1, meanwhile, were expressed higher in the granuloma compared with the surrounding tissue. These data support the presence of *MMP1*^*+*^*CXCL5*^*+*^ fibroblasts in TB-diseased human lung and their localization with TB granuloma. It is worth noting, however, that some differences in fibroblast populations we observe between TB conditions may be exacerbated by the limited number of control lung samples and difficulties associated with extracting stromal cells from fresh tissues during single-cell isolation (Guilliams et al., 2022).

### Reference mapping to HLCA reveals distinct activities between TB-diseased and control fibroblasts

Given the limited recovery of fibroblasts from TB-negative controls, we next explored how the fibroblast subsets detected in TB-diseased lung tissue relate to lung fibroblasts in published datasets. For this, we used the data from the HLCA, which integrates 49 datasets from the human respiratory system, encompassing 2.4 million single cells, to generate consensus cell type annotations (Sikkema et al., 2023). Using the HLCA as a reference, we confirmed the heterogeneous immune and nonimmune cell types present in our lung tissue samples (Fig. 4 D). Via label transfer, we independently re-annotated our fibroblasts, observing high consistency with our original annotations (>95% fibroblasts were re-annotated as fibroblast/myofibroblast; Materials and methods). Combining cells mapped to fibroblasts/myofibroblasts in our data and the HLCA, we performed differential expression (DE) analysis between all TB-negative cells (mostly consisting of healthy cells from the HLCA reference) and our TB-diseased cells (Fig. 4 E). We then ran gene set enrichment analysis (GSEA) on the resulting top differentially expressed (DE) genes using the MSigDB Hallmark database (Fig. 4 F). This confirmed upregulation of EMT processes, thought to directly contribute to the fibroblast/myofibroblast pool during fibrosis, in TB-diseased fibroblasts. In addition, oxidative phosphorylation was highly upregulated, consistent with alteration of metabolic activity in fibrotic lung disease (Geng et al., 2021). Several enriched terms are related to inflammatory process, including TNF-α signaling, TGF-β signaling, IL2/IL6 signaling, and were also observed, suggesting an overall elevated inflammatory response in the TB fibroblasts.

### Identification of fibroblast gene modules associated to bacterial burden within TB granuloma

**Figure 5.**
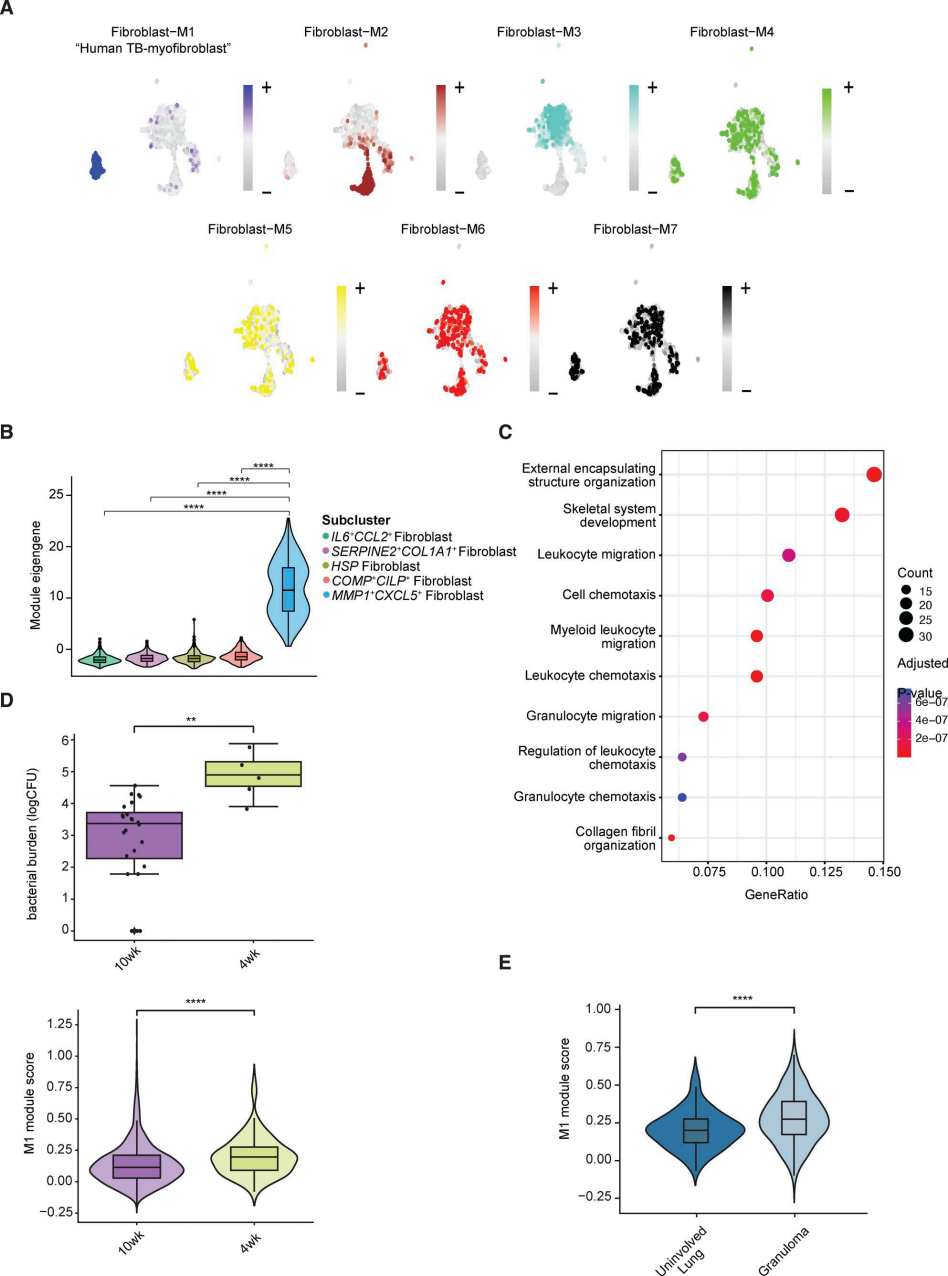
**Fibroblast** **WGCNA** **(hdWGCNA). (A)** High-dimensional WGCNA (hdWGCNA) for gene module detection in fibroblasts of this study. UMAPs are colored by eigengene of each of the seven modules. **(B)** Evaluation of M1 module expression in fibroblast subclusters. Bonferroni-adjusted P computed from two-sided Wilcoxon test is shown. **(C)** ORA by enricher on all assigned M1 module genes using MSigDB Gene Ontology Biological Processes (GOBP) gene set database. **(D)** Top: Bacterial burden of NHP lung granulomas by Gideon et al. (2022) grouped by the time point. Bottom: Evaluation of human TB-myofibroblast module expression in NHP TB fibroblasts on 4-wk and 10-wk samples. Two-sided Mann–Whitney U test without correction was used. Statistical annotations: P value < 0.05 (*), P value < 0.01 (**), and P value < 0.001 (***). **(E)** Evaluation of human TB-myofibroblast module expression in fibroblasts from granuloma vs uninvolved lungs in an independent NHP study with 4-wk post-infection (p.i) macaques (Bromley et al., 2024) (Materials and methods). Two-sided Mann–Whitney U test without correct was used. Statistical annotations: P value < 0.01 (**) and P value < 0.0001 (****).

Having compared our fibroblast subclusters against public lung stromal datasets, we next examined how the cell states within these subclusters might shift with TB disease in an experimentally controlled setting. Given the paucity of TB-associated gene signatures, we opted to pursue unbiased gene module identification on the entire fibroblast population, applying a tool for weighted gene co-expression network analysis (WGCNA) in high-dimensional single-cell transcriptomics data (hdWGCNA) (Morabito et al., 2023). This yielded seven gene modules with varying degrees of expression across the five clusters and disease states (named fibroblast-M1–7; Fig. 5 A and Data S11). The fibroblast-M1 module was highly enriched in the *MMP1*^*+*^*CXCL5*^*+*^ fibroblast cluster (Fig. 5 B). Top hub genes in this module included: *MMP1, CA12, CXCL5, CXCL13, TDO2, PDPN,* and *FAP*, showing a high degree of overlap with cluster markers for *MMP1*^*+*^*CXCL5*^*+*^ fibroblast (Fig. 4 B and Fig. S4, C and D). There was also a clear overlap between *SERPINE2*^*+*^*COL1A1*^*+*^ fibroblasts and fibroblast-M2 module (Fig. 4 B and Fig. S4 D). ORA showed the M1 module was highly enriched for known biological processes associated with immune cell migration and chemotaxis (“myeloid leukocyte migration,” and “granulocyte chemotaxis”) and control of ECM structure (“external encapsulating structure,” “collagen fibril organization,” and “ECM disassembly”), as well as myofibroblast-related signatures (Talbott et al., 2022) (“response to wounding,” “muscle tissue development,” “myoblast differentiation,” and “response to mechanical stimulus”) (Fig. 5 C and Data S8). Hence, we refer to this M1 module, enriched in the *MMP1*^*+*^*CXCL5*^*+*^ fibroblast population, as the “human TB-myofibroblast” module.

To investigate these modules in the context of defined pulmonary TB granuloma, we evaluated the expression of each module in fibroblasts from a well-controlled SIV-uninfected NHP TB granuloma dataset by Gideon et al. mentioned above (Gideon et al., 2022). This study collected data on positron emission tomography (PET)-tracked granulomas isolated at 4 and 10 wk after infection, with each granuloma individually resected, homogenized, and subjected to scRNA-seq, as well as quantification of total and viable *M*.*tb*. Interestingly, our human TB-myofibroblast module (M1) and M2 and M3 modules were significantly elevated in the week 4 granulomas, which contained higher *M.tb* burdens, compared with those at week 10 (Fig. 5 D and Fig. S4 E). These data suggest that the human TB-myofibroblast phenotype, in addition to other diverse fibroblasts, is likely present in untreated early TB lung granuloma, and that their frequency is associated with bacterial burden. Next, to further localize human TB-myofibroblast phenotype relative to granuloma, we evaluated the expression of this module in fibroblasts in an independent dataset of SIV-uninfected NHP TB lung TB dataset, which included single-cell data from uninvolved lung tissue (Bromley et al., 2024; Ganchua et al., 2024). From this dataset, lung granulomas from 4 wk p.i. (granuloma data published by Bromley et al.) were compared against uninvolved lung samples from the same experimental condition (Bromley et al., 2024) (uninvolved lung data unpublished, Fig. 5 E and Table S3; Materials and methods). Evaluating our hdWGCNA modules, we observed that the human TB-myofibroblast module was upregulated in the granuloma compared with uninvolved lung tissues, confirming that this phenotype is associated with granuloma-specific structural or cellular processes that reflect a local response to *M.tb*.

### Confirmation of the myofibroblast-like phenotype in different TB disease contexts

**Figure 6.**
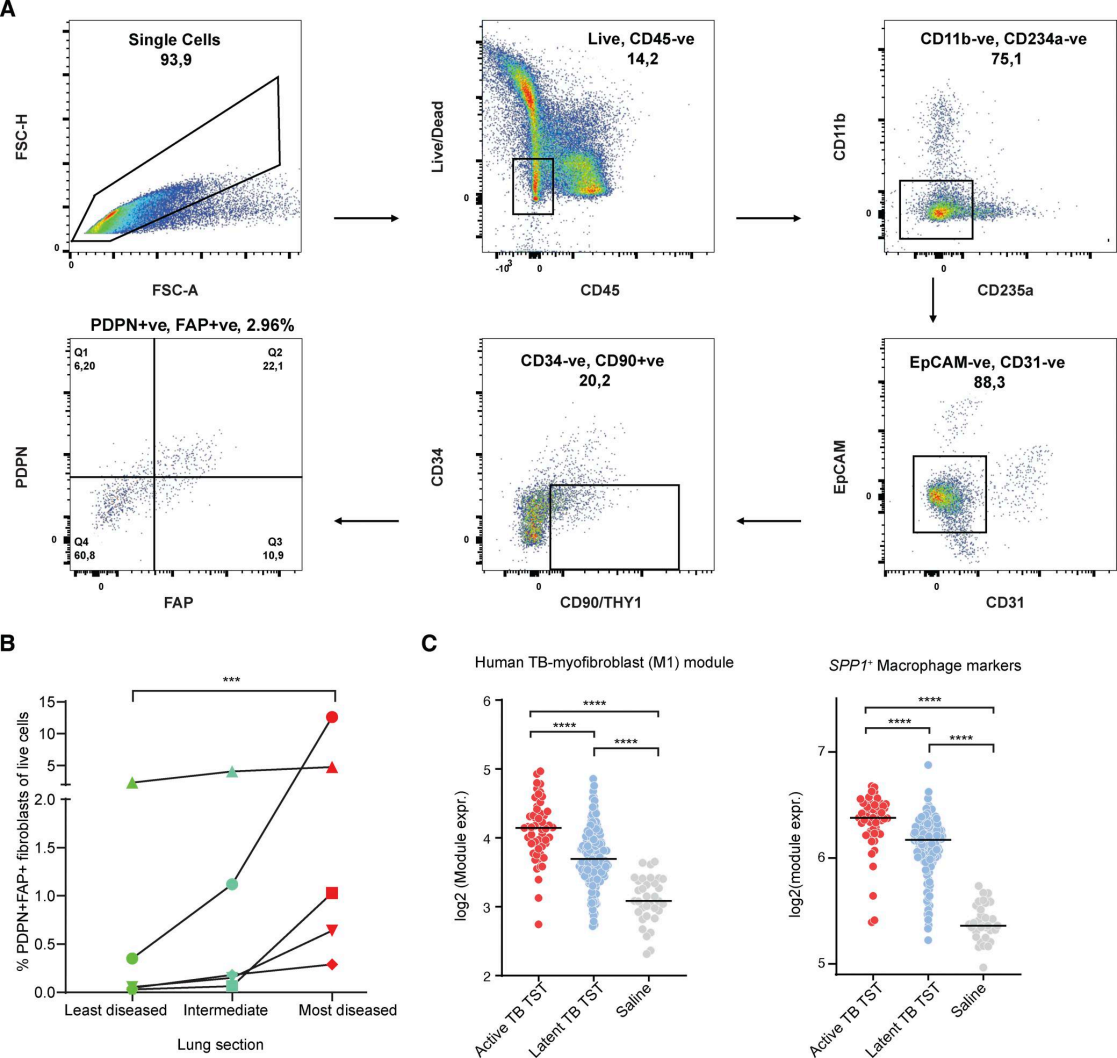
**Evidence of *MMP1*** ^***+***^ ***CXCL5*** ^***+***^ **fibroblast populations in TB-diseased human lungs. (A)** Representative flow cytometry plot showing the isolation strategy of the PDPN^+^FAP^+^ fibroblast population from the CD45-EPCAM cell fraction. **(B)** Cumulative data on frequency of PDPN^+^FAP^+^CD90^+^ fibroblasts as a fraction of live lung cells from five separate lung resections. Three separate sections were taken from each TB-diseased lung, corresponding to the most diseased and least diseased tissues areas and an intermediate lung piece, according to the expert opinion of the operating surgeon. The Friedman test was used to ascertain statistical significance between proportion of PDPN^+^FAP^+^ fibroblast between severity groups. **(C)** Expression of human TB-myofibroblast signature and *SPP1*^+^*CHI3L1*^+^ marker genes in the TST challenge site *in vivo* model. Active TB TST (*n* = 48): biopsies from participants with microbiologically confirmed pulmonary TB disease within the first month of treatment who underwent TST; latent TB TST (*n* = 191): biopsies from participants lacking clinical and radiological evidence of active TB disease but with a positive peripheral blood IFN-γ release assay; saline (*n* = 34): biopsies from participants that received saline under the skin instead of tuberculin. Each dot corresponds to a sample; horizontal lines represent median values. Two-sided Mann–Whitney U test without correct was used. Statistical annotations: P value <0.001 (***), P value < 0.0001 (****).

Taken together, our findings suggest a previously underappreciated role for fibroblasts—including a myofibroblast-like *MMP1*^*+*^*CXCL5*^*+*^ subcluster—in TB immunopathology. However, while this population was detected in 5/9 TB-diseased samples, the majority of cells were derived from a single donor. Therefore, we quantified this fibroblast subset in additional patients undergoing surgery for post-TB lung complications by flow cytometry (Table S4). For this, we gated on non-hematopoietic cells (CD45^−^), lacking expression of CD234a (Duffy antigen), CD31 (endothelial cells), EPCAM (epithelial cells), and CD34 (progenitors) but expressing the fibroblast marker CD90, as well as PDPN and FAP, which are both canonical myofibroblast markers and hubs genes in our human TB-myofibroblast module (Fig. 6 A). These data confirmed the presence of PDPN^*+*^FAP^*+*^ fibroblasts in 5/5 TB-diseased lung samples. In addition, by examining tissue samples from regions of lung tissue with varying degree of disease pathology, as determined by operating surgeon, we found that PDPN^*+*^FAP^*+*^ fibroblasts were elevated in the most diseased lung pieces (P value = 8 × 10^−4^, Friedman test; Fig. 6 B).

To determine whether a *M.tb* stimulation *in vivo* could induce these TB-associated fibroblast gene signatures, we evaluated the expression of our TB-myofibroblast gene module in a previously published bulk transcriptomics dataset from a standardized tuberculin skin test (TST) challenge (Fig. 6 C; Materials and methods) (Pollara et al., 2021). In this study HIV^−^ participants with active pulmonary TB or “latent TB” (i.e., individuals with T cell memory to *M.tb* but no evidence of TB disease) received a standard TST (intradermal injection of purified *M.tb* proteins) challenge or saline control. The TST site was biopsied 48 h later and processed for bulk RNA-seq. Consistent with our observations, the human TB-myofibroblast signature was induced in response to the standardized mycobacterial antigen stimulation *in vivo* compared with saline controls, where no inflammatory response is expected. This signal was amplified in the context of active TB disease compared with latent TB. This implies that systematic inflammation from active *M.tb* infection may prime the differentiation of a pathological fibroblast cell state. Interestingly, genes associated with the *SPP1*^*+*^*CHI3L1*^*+*^ macrophage subset were similarly induced by TST, supporting the hypothesis that *M*.*tb* stimulation induces myofibroblast-like phenotype and *SPP1*^*+*^ macrophages in humans, especially in the context of active TB disease (Fig. 6 C).

### Cell–cell interaction analysis reveals fibroblasts dominate cellular cross talk in TB-diseased lung

**Figure 7.**
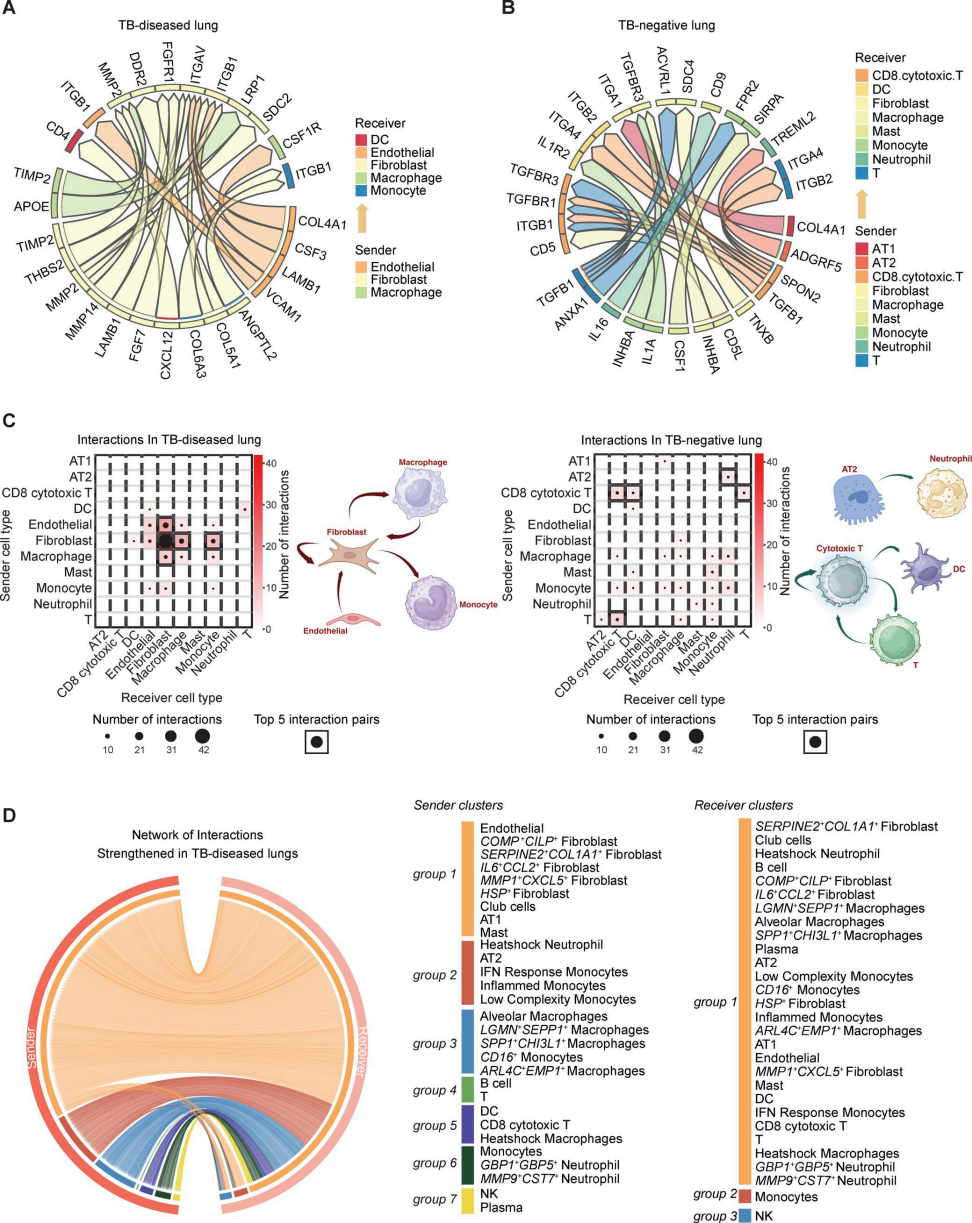
**Cell–cell interaction analysis reveals key discrepancies between TB-diseased and control lung niches. (A)** Top 20 ligand–receptor (L–R) pairs from MultiNichenet analysis highlighting putative interaction pairs with upregulated interactions in TB-negative lung compared with TB-diseased lung. **(B)** Top 20 ligand–receptor (L–R) pairs from MultiNichenet analysis highlighting putative interaction pairs differentially communicating in TB-diseased lungs. **(C)** Summary of top 200 interactions in TB-diseased and TB-negative/control lungs by the number of interactions between each cell pair. Cartoons on the right of each heatmap show the suggested major modes of interactions in each condition. **(D)** Circos plots of significant interaction pairs in TB-diseased lungs from LIANA, where sender and receiver cell types in each condition are clustered to reflect similar patterns.

To identify putative intercellular interactions regulating differentially expressed genes between TB-diseased and control lung niches, we used MultiNichenet (Browaeys et al., 2020) (Fig. 7, A and B; Materials and methods). This indicated that fibroblasts were both the dominant sender and receiver cell type in TB-diseased lungs (Fig. 7 C). Interestingly, a significant proportion of fibroblasts expressed ligand and receptor pairs consistent with autocrine signaling. In contrast, top interactions in the TB-negative condition were more diverse, with pronounced T cell and myeloid involvement.

We performed additional analysis with LIANA to drill down into the specific cellular subclusters contributing to niche cross talk in TB infected and control lungs (Dimitrov et al., 2022) (Data S3, A and B; and Data S4 A). This integrated ligand–receptor analysis framework leverages multiple resources and methods to generate aggregated inference on samples from each condition. Our results suggested a dominant role for *COMP*^*+*^*CILP*^*+*^, *IL6*^*+*^*CCL2*^*+*^*, MMP1*^*+*^*CXCL5*^*+*^*,* and *SERPINE2*^*+*^*COL1A1*^*+*^ fibroblasts, but not *HSP* fibroblasts, in TB-diseased conditions. This analysis also implied that AT1 cell sender signaling is upregulated in the TB-diseased lung, although these cells were significantly depleted in TB-diseased lungs (Fig. 2 D and Data S4 B). AT1 cells normally serve as the interface of oxygen exchange in the alveoli, but we found they have high expression of collagen in the TB-diseased lungs, which broadly targets other cell types (Data S4 B). In TB-negative diseased lungs, only *HSP* fibroblasts were predicted to contribute to signaling (Data S3 B). It is important to note, however, that the lack of fibroblasts in TB-negative lungs may influence this analysis.

**Figure 8.**
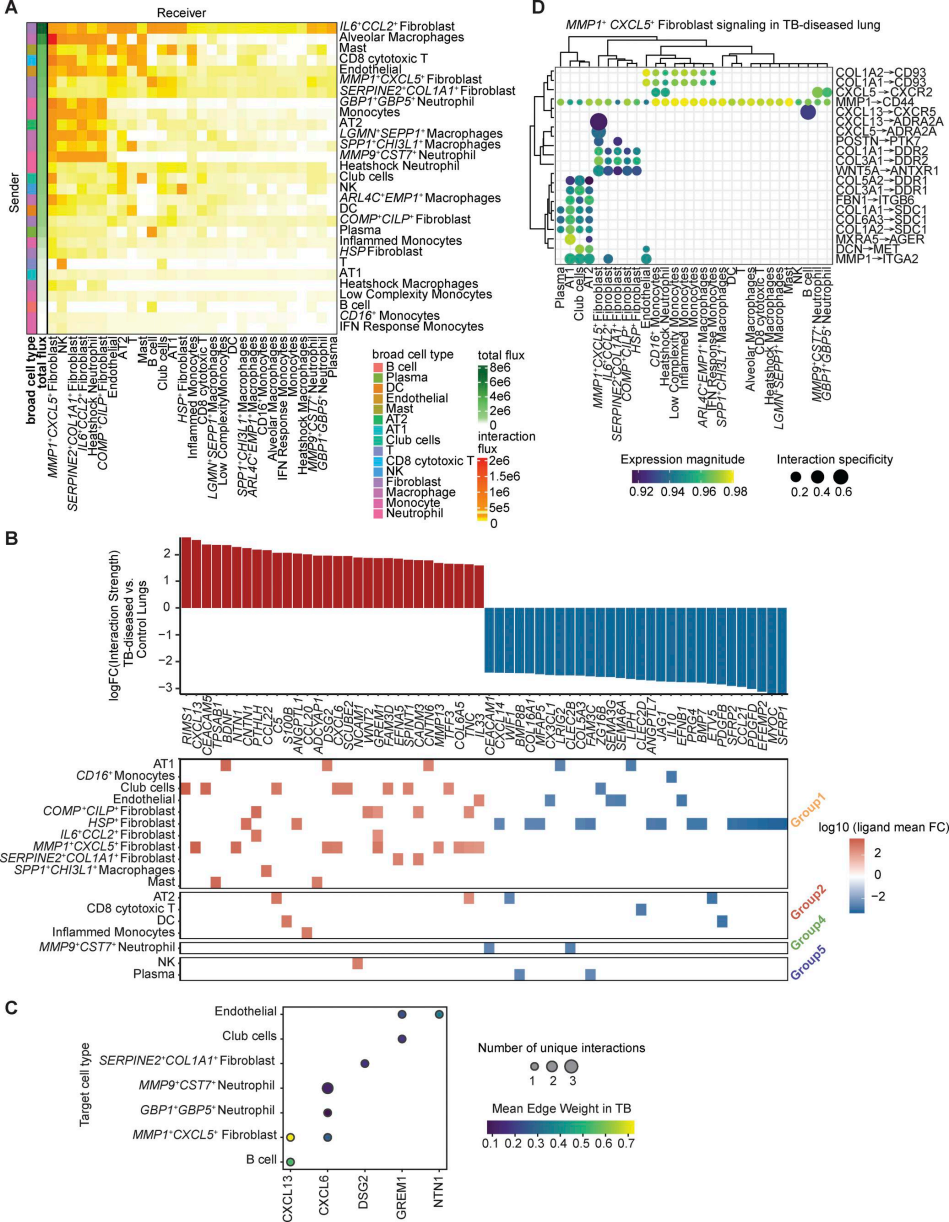
**Global interaction analysis identifies key players in cellular communication within TB-diseased lung tissues. (A)** Heatmap visualization of interaction flux analysis. Rows represent sender cell types; columns represent receiver cell types. Each entry represents the potential flux of interaction from sender cell to receiver cell, whereas the total flux of each sender cell type is summarized on the left. Sender cell types are sorted based on descending order of total flux (Materials and methods). **(B)** Top: Bar plot showing top 30 and bottom 30 ligands by log fold-change of interaction strength between TB and control lungs across all sender cell types. Bottom: Log fold-change of interaction strength between TB and control lungs in each sender cell type (Materials and methods). **(C)** Dot plot of top five ligand by ligand activity in TB-diseased lungs secreted by *MMP1*^*+*^*CXCL5*^*+*^ fibroblasts and their receivers (Materials and methods). **(D)** L–R interactions with *MMP1*^*+*^*CXCL5*^*+*^ fibroblasts in the TB-diseased lungs; rows (L–R pair) and columns (target cell types) are hierarchically clustered by correlation distance (Materials and methods). L–R, ligand–receptor.

To understand broader signaling patterns, we grouped sender and receiver cell types based on similarities of their signaling patterns (Materials and methods). Within TB-diseased samples, we observed more distinctive patterns among sender cell types than receivers, with senders roughly grouped by cell type (Fig. 7 D). The opposite was observed in TB-negative lung (Data S4 C). In TB, most of the fibroblast sender subclusters (*MMP1*^*+*^*CXCL5*^*+*^, *COMP*^*+*^*CILP*^*+*^, and *IL6*^*+*^*CCL2*^*+*^) grouped together and with other nonimmune cells (endothelial cells, AT1, and club cells; sender group 1). Quantification of net cell signaling flux—defined as the product of a sender population’s relative abundance and its average expression of a given signal—highlighted that, despite making up a small proportion of the entire dataset, *MMP1*^*+*^*CXCL5*^*+*^ fibroblasts were among the most prolific signal senders in the TB-diseased condition (Fig. 8 A).

Next, to quantify ligand-driven changes in cellular cross talk during TB infection, we calculated the difference in ligand interaction strengths between TB-diseased and TB-negative lung samples (Fig. 8 B; Materials and methods). TB sender group 1 secreted most of the top upregulated ligands in TB-diseased lung (binomial test P value < 0.01, Data S5 A, left). Top senders of all upregulated interactions in TB-diseased lungs were *COMP*^*+*^*CLIP*^*+*^ fibroblasts, followed by *MMP1*^*+*^*CXCL5*^*+*^ fibroblasts (Data S5 A, right). In contrast, control sender group 2, which consists mostly of monocytes and macrophages, exhibited the greatest signaling flux in control lung (binomial test P value < 0.01, Data S5 B). Notably, *MMP1*^*+*^*CXCL5*^*+*^ fibroblasts expressed most of the top flux ligands (9/30) with increased overall interaction strength in TB, supporting a central role in TB disease (Fig. 8 B). The top five ligands from the *MMP1*^*+*^*CXCL5*^*+*^ fibroblasts, ranked by average interaction strength across receptors, were CXCL13, CXCL6, DSG2, GREM1, and NTN1 (Fig. 8 C). CXCL13 may act in both autocrine and paracrine modes, signaling to B cells via CXCR5, a homing marker for activated lymphocyte to lymphoid tissues, and on B cells in NHP lung TB granuloma, where it regulates host–pathogen interactions (Loxton, 2019). CXCL6 appears to function similarly, signaling to neutrophils and self, consistent with known functions in inducing fibroblast matrix expression, neutrophil recruitment, and activation (Bahudhanapati et al., 2021, *Preprint*; Mittal et al., 2008). DSG2 (desmoglein) is known to induce pro-proliferative activity in dermal fibroblasts (Overmiller et al., 2017) and is highly upregulated in zebrafish and human granuloma (Cronan et al., 2016). GREM1, part of the TGF-β superfamily, contributes to pulmonary fibrosis during the early stages of disease (Shi et al., 2022). NTN1 (netrin-1), meanwhile, supports endothelial survival and regulates angiogenesis, an important process for dissemination of the pathogen (Castets et al., 2009; Polena et al., 2016).

We also examined other top ligands sent by this subcluster in TB-diseased lungs specifically. Our analyses suggested a prominent role for the *MMP1*^*+*^*CXCL5*^*+*^ subcluster in coordinating fibrosis and inflammation through expression of collagen proteins, *MMP1*, and cytokines (Fig. 8 D). Notably, MMP1 itself acts as a ligand for ITGA2, a receptor expressed on epithelial cells, endothelial cells, and *IL6*^*+*^*CCL2*^*+*^ fibroblasts, mirroring the AT1–fibroblast interactions aforementioned. CXCL5 interacts with CXCR2 on *CD16*^*+*^ monocytes and neutrophils, a key axis for recruitment of these cells during TB infection and likely fueling granuloma formation (Nouailles et al., 2014; Sawant et al., 2016; Serbina et al., 2008). MMP1^+^*CXCL5*^*+*^ fibroblasts also secrete numerous ECM-related ligands: for instance, collagen molecules *COL1A1, COL1A2, COL6A3, COL3A1,* and *COL5A2* signal to epithelial cells and monocytes, in addition to other fibroblasts via an autocrine loop; ECM proteins *POSTN, FBN-1*, and *DCN* signal with other nonimmune cell types; and, *MXRA5*, a matrix remodeling protein like *MMP1*, communicates to AT1/AT2 cells via *AGER*. Notably, many of these ECM-related ligands are highly upregulated in LN TB granuloma (Data S5 C). Collectively, our analyses suggest aberrant lung remodeling may be driven by fibroblast and AT1 communication, leading to the fibrosis typical of TB—a trend not necessarily reflected from cell type abundance changes.

### Cell–cell interaction analysis underscores the relevance of *SPP1*^*+*^ macrophages in human TB

**Figure S5.**
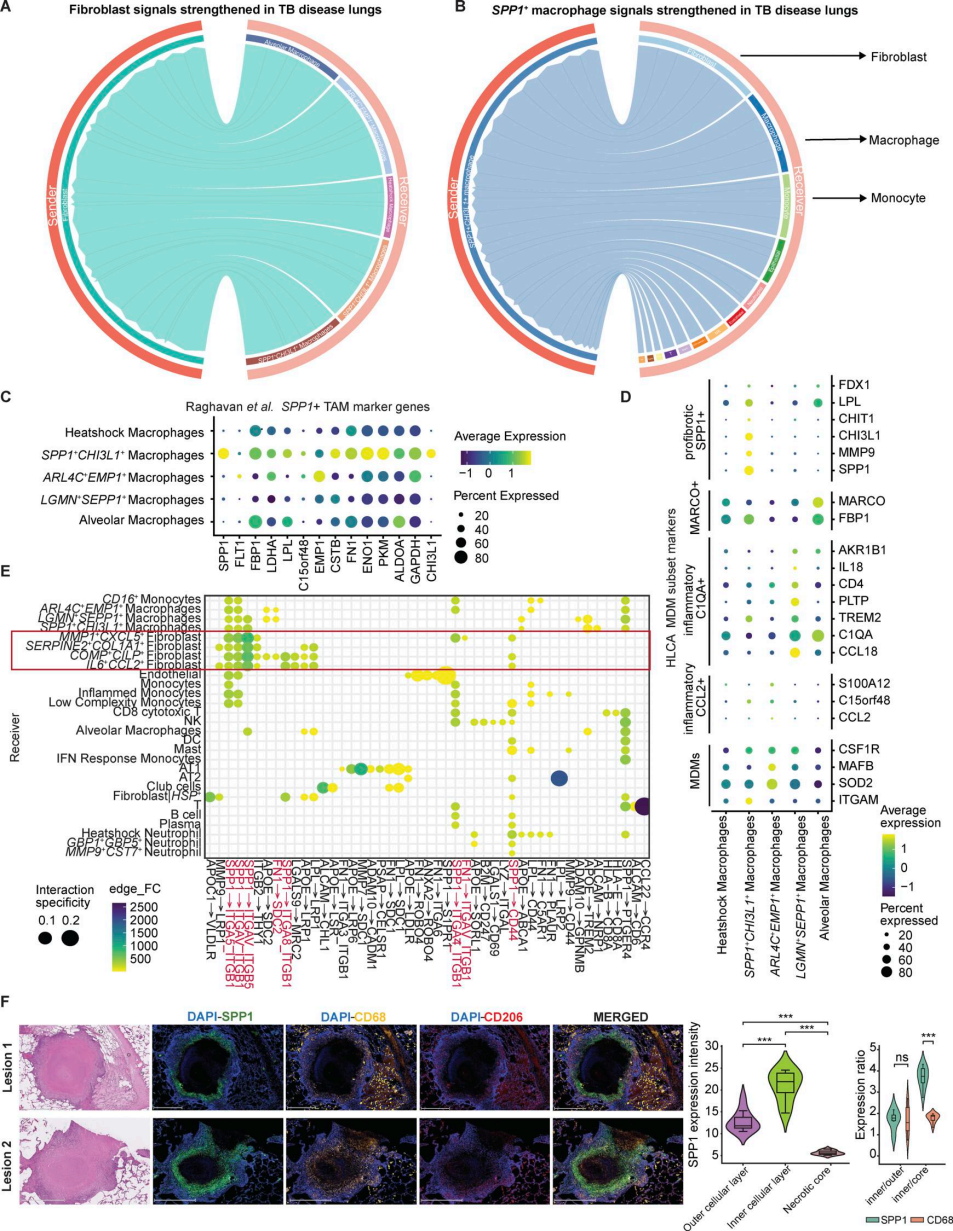
***SPP1* ^+^ macrophage interaction with fibroblasts in TB. (A)** Circos plot showing proportion of inferred signals upregulated in TB-diseased lungs from LIANA analysis with fibroblasts as sender and macrophage subclusters as receivers. **(B)** LIANA analysis on detailed cell level with *SPP1^+^* macrophage as sender. Circos plot showing proportion of inferred signals upregulated in TB-diseased lungs sending from *SPP1*^+^ macrophage to broad cell groups, with fibroblasts at the top. **(C)** Evaluation of markers gene expression for *SPP1^+^* TAM from PDAC by Raghavan et al. (2021) on macrophage subclusters in this study. **(D)** Evaluation of marker gene expression for monocyte-derived macrophage (MDM) subsets from the HLCA on macrophage subclusters in this study. **(E)** Specific upregulated ligand–receptor pairs and involved receiver cells in the LIANA analysis with *SPP1^+^* macrophage as sender. **(F)** Left: Fluorescence immunohistochemistry staining images of human lung granuloma with DAPI (nuclear), SPP1 (*SPP1*^*+*^ macrophage), CD68 (pan macrophage marker), and CD206 (macrophage enriched in alveolar spaces). Scale bars: 1 mm. Middle: Quantified SPP1 expression from 10 ROIs (5 μm/pixel) in outer cellular layer, inner cellular layer, or the necrotic core of the granulomas. Right: Expression ratio of SPP1 and CD68 between inner cellular layer and outer layer and inner cellular layer and necrotic core. The images in F come from serial sections of the same TB granuloma depicted by Mpotje et al. (2025), *Preprint*. As both studies identify macrophages, the macrophage stain CD68 is shown in both. However, this current study colocalizes with CD206 and SPP1, while the preprint co-stains with ALOX5. Two-sided Mann–Whitney U test without correction was used on each module. Statistical annotations: P value < 0.001 (***); P value > 0.05 (ns).

The main receivers of fibroblast signaling were fibroblasts and macrophages (Fig. 7 C). Among macrophage subclusters, the top receivers for TB-upregulated fibroblast signals were the *ARL4C*^*+*^*EMP1*^*+*^ and *SPP1*^*+*^*CHI3L1*^*+*^ cells (Fig. S5 A). Conversely, *SPP1*^*+*^*CHI3L1*^*+*^ subcluster mostly signals to fibroblasts, followed by macrophages (Fig. S5 B), suggesting a potentially important role in fibroblast-macrophage cross talk. *SPP1*^*+*^ macrophages have been identified in the lungs of individuals with COVID-19, IPF, and lung carcinoma and in BAL fluids from TB and latent TB patients (Sikkema et al., 2023; Yang et al., 2023). In tumors, SPP1^+^ macrophages are highly immunosuppressive and associated with poor outcomes, and they have been shown to orchestrate fibroblast activation during fibrosis, driving myofibroblast activation in heart and kidney injuries (Gao et al., 2022; Hoeft et al., 2023; Matsubara et al., 2022). Comparing against other known markers, we noted that our *SPP1*^*+*^*CHI3L1*^*+*^ macrophages appeared congruent with *SPP1*^*+*^ macrophages described in other disease contexts (Fig. S5, C and D). Cell–cell interaction analysis showed that fibroblasts were the major receiver of *SPP1*^*+*^*CHI3L1*^*+*^ macrophage signals (binomial test P value = 8.7 × 10^−14^), and nominated *SPP1* and *FN1* as the major ligand genes driving cross talk with fibroblasts (Fig. S5 E).

To further confirm the presence of *SPP1*^*+*^ macrophages in human lung TB granulomas, we performed immunohistochemical staining of tissues from two independent donors. We observed abundant total macrophages (CD68^+^) in both the granuloma and surrounding lung tissue and localization of alveolar macrophages (CD68^+^CD206^+^) in the non-granulomatous lung tissue, where alveolar sacs were still visible (Fig. S5 F, left). In stark contrast, CD68^+^SPP1^+^ macrophages localized to the inner cellular periphery immediately bordering the necrotic core of the granuloma and were largely absent from surrounding lung tissue. Quantification of SPP1 expression shows a significant difference between the inner cellular layer and the other regions and to a larger degree than CD68 (Fig. S5 F, right). Notably, CTHRC-1, a marker for *MMP1*^*+*^*CXCL5*^*+*^ fibroblasts that was localized to granuloma at the protein level, has been suggested to play a role in cross talk with *SPP1*^*+*^ macrophages (Liu et al., 2022) (Fig. S4 B). These lines of evidence support the direct interaction between *SPP1*^*+*^ macrophages and myofibroblast-like phenotype in human TB granuloma implied by the single-cell data.

### Spatial transcriptomics confirms myofibroblast-like phenotype in independent human cohort

**Figure 9.**
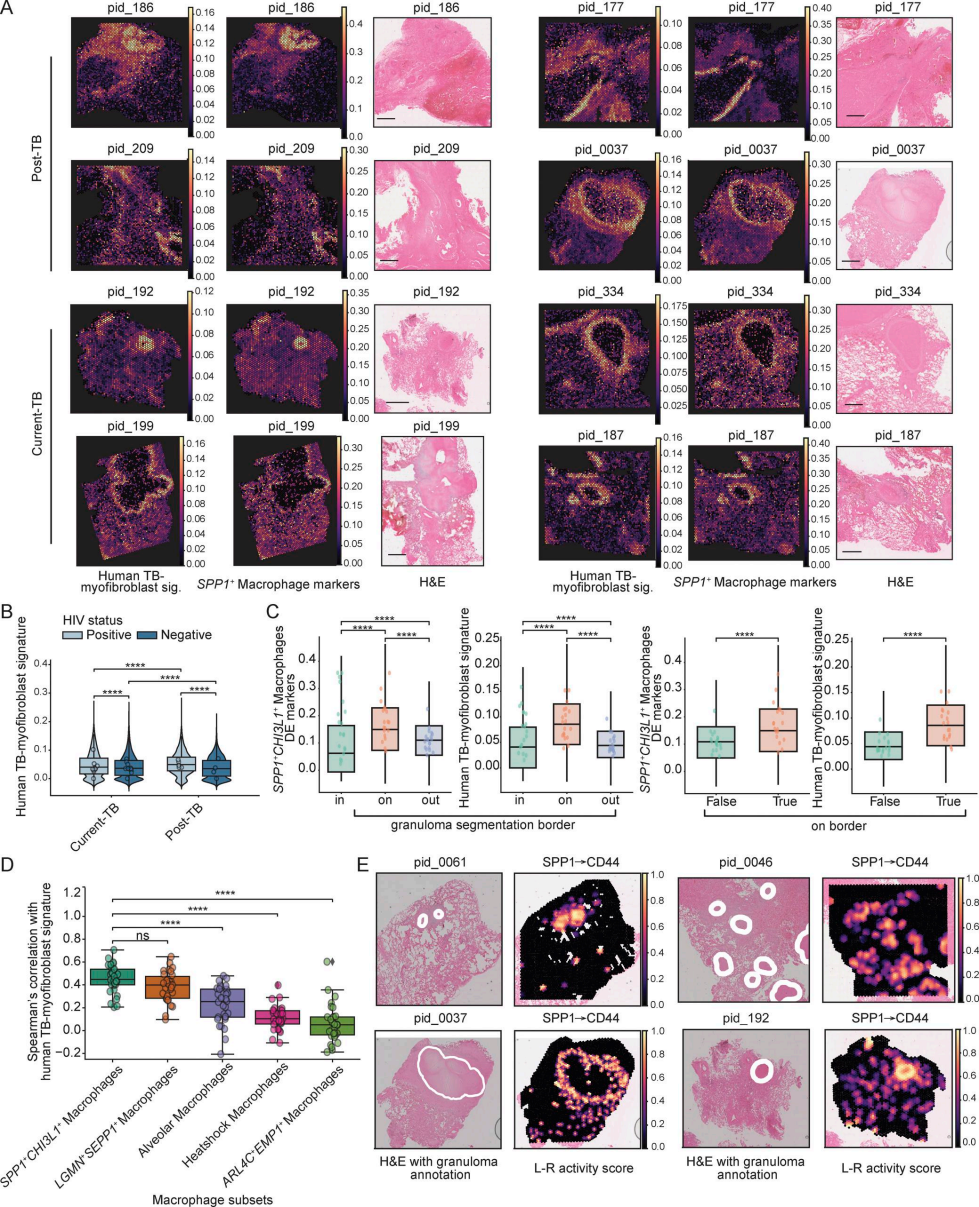
**Spatial transcriptomics analysis on post- and current TB lung resections. (A)** Heatmap showing the expression of human TB-myofibroblast gene signature and *SPP1*^+^*CHI3L1*^+^ macrophage markers on selective tissue slides from patients who are post-TB (top) or current TB (bottom), alongside paired H&E staining (these H&E stains are also shown in Fig. S1 A together with those other samples used for spatial transcriptomics not shown here). **(B)** Distribution of human TB-myofibroblast signature expression on the spatial cohort. HIV statuses are shown in different shades of blue for positive or negative. Two-sided Mann–Whitney U test without correction was used for statistical testing. Statistical annotation: P value < 0.0001 (****). **(C)** Distribution of *SPP1*^+^*CHI3L1*^+^ macrophage markers and human TB-myofibroblast signature on the spatial data across all Visium spots. Left two panels: Manual segmentation of the granuloma structure was done to allow separation of the Visium slide into three different regions: in granuloma, on granuloma border (cuff), and outside of granuloma (Materials and methods). Right two panels: The same as left panels with the exception that “on border” = True means on granuloma cuff and False means the rest. Two-sided Mann–Whitney U test without correction was used for statistical testing. Statistical annotation: P value < 0.0001 (****). **(D)** Correlation between human TB-myofibroblast signature and all macrophage subpopulations’ markers. Each circle represents a Visium sample. Boxplot of the Pearson’s r distribution is shown for each macrophage subtype. Mann–Whitney U test without correction were used for statistical testing. Statistical annotation: P value < 0.0001 (****). **(E)** Spatially informed ligand–receptor (L–R) analysis using LIANA+ on Visium samples. Examples are shown where SPP1(L)–CD44(R) interactions are being nominated as top L–R pairs. H&E overlaid with pathology annotation for granuloma structures are shown next to heatmap of L–R interaction scores, which are calculated at each Visium spot using spatially weighted Cosine similarity (Materials and methods).

Finally, to confirm our observations from human TB lung and LN granulomas, we investigate cells within the Visium dataset for expression of the myofibroblast-like module (Table S5, Fig. 1 B, and Fig. S1 A). Consistent with our other data, the human TB-myofibroblast signature was detected in both current and post-TB lesions and was particularly highly expressed around granuloma structures (Fig. 9 A). In addition, we found that both HIV^+^ and HIV^−^ samples displayed clear human TB-myofibroblast signature expression, suggesting it is not limited to TB mono-infected individuals, as potentially suggested by our single-cell data (Fig. 4 A). Indeed, in both current and post-TB samples, HIV was associated with a higher TB-myofibroblast signature expression (Fig. 9 B). This may be because HIV impairs CD4^+^ T cells and macrophage-driven repair and increases TGF-β release, keeping myofibroblast-like cells chronically active (Joseph et al., 2022; Meng et al., 2016; Theron et al., 2017). In HIV^−^ samples, current TB was associated with elevated expression of the myofibroblast-like module, but the opposite was true in HIV^+^ samples, likely due to persistent systemic immune activation from HIV. These observations suggest that both pathogens can exacerbate the expression of this disease-associated module. Within each disease condition, we found granuloma samples had higher human TB-myofibroblast signature expression, with the exception of HIV^+^ post-TB group, where expression was highest in the iBALT sample (Data S6 A). However, only one iBALT sample was available for this condition, which limits our confidence in the observation. Nevertheless, these data confirm that the human TB-myofibroblast phenotype is localized to human TB lung granuloma in both active TB and PTLD, irrespective of concurrent HIV infection.

For each of the granuloma samples, annotations on granuloma borders (“granuloma cuff”) were designated in paired H&E staining images by a trained histopathologist and used to examine the spatial distribution of gene signatures (Fig. S1 B). This analysis confirmed that the human TB-myofibroblast signature was strongly expressed in the granuloma cuffs compared with surrounding regions, with a slightly higher presence outside the granuloma compared with the granuloma core (Fig. 9 C). Interestingly, examining the other fibroblast modules revealed distinct spatial orientation relative to the granuloma (Data S6 B). Like *MMP1*^*+*^*CXCL5*^*+*^ fibroblasts*, COMP*^*+*^*CLIP*^*+*^ and *SERPINE2*^*+*^*COL1A*^*+*^ fibroblasts displayed a similar pattern of enrichment around the granuloma cuff, whereas *IL6*^*+*^*CCL2*^*+*^ fibroblasts and *HSP* fibroblasts exhibited greater enrichment outside the granuloma. *MMP1*^*+*^*CXCL5*^*+*^ fibroblast, however, showed the largest difference for marker expression between the Visium spots on the granuloma cuff and those inside/outside the cuff.

*SPP1* ^*+*^ *CHI3L1* ^*+*^ macrophage marker expression was similarly enriched on the granuloma cuff, supporting the colocalization of myofibroblast-like phenotype and *SPP1*^*+*^*CHI3L1*^*+*^ macrophages at this site (Fig. 9 C). To confirm this relationship, we looked at the correlation between all macrophage subset markers with the human TB-myofibroblast signature across all samples and found the strongest correlation with *SPP1*^*+*^*CHI3L1*^*+*^ macrophages compared with the other macrophage subsets (Fig. 9 D). Finally, we conducted a ligand–receptor analysis to identify spatially co-expressed ligand–receptor pairs using the same database as our analysis on scRNA-seq data (Materials and methods). This identified the same L–R pairs as the top pairs in both samples, including, for example, SPP1–CD44. This interaction was nominated as the top L–R pair in several samples, specifically highlighted around the granuloma cuffs and in our scRNA-seq data (Fig. 9 E and Fig. S5 E).

Taken together, our scRNA-seq and spatial transcriptomics data support the robustness and generalizability of the human TB-myofibroblast signature and confirm its colocalization and cross talk with *SPP1*^*+*^ macrophages in human TB lung granuloma.

## Discussion

TB is a global pandemic, and transformative interventions are hindered by an incomplete understanding of its pathogenic processes, including the extensive lung remodeling in pulmonary TB that drives transmission, mortality, and a high burden of PTLD following successful treatment (Dheda et al., 2016). Several sequencing studies have highlighted a central role for ECM remodeling of the human lung in TB, but none have resolved the contributions of individual cell types (Elkington et al., 2022). Additionally, an emerging issue in TB research is that findings from the circulation—the compartment mostly frequently studied—often fail to reflect processes in diseased tissue (Ogongo et al., 2020). To address these gaps, we analyzed scRNA-seq data generated from lung tissue freshly resected to treat complication arising from TB disease and systematically cross-referenced our findings with public datasets from *M.tb*-infected NHPs, the HCLA, LN TB granulomas, and TST challenge, as well as additional immunohistochemical, flow cytometric, and spatial transcriptomic data from the same cohort to identify TB-specific changes at the cellular level. Collectively, our lung datasets provide a key resource defining the cellular subsets present in TB-diseased lung and dissecting immunopathogenic mechanisms. Our data demonstrate substantial heterogeneity among key innate immune populations, such as macrophages and neutrophils, in infected lung tissue. We find that several of these subsets correlate with a recent single-cell analysis of *M.tb*-infected NHPs (Gideon et al., 2022), a study not limited by tissue availability or complicated by comorbidities, such as HIV, further strengthening our observations. In addition, our data highlight a possible central role for diverse fibroblast subsets with TB-diseased lung tissue and with TB granuloma, particularly an underappreciated *MMP1*^*+*^*CXCL5*^*+*^ fibroblast population that colocalizes with *SPP1*^*+*^ macrophages at the granuloma cuff. We hypothesize that the interaction between these cells, which express a myofibroblast-like gene module, and *SPP1*^*+*^ macrophages may play an important role in human TB granuloma development and PTLD, potentially aggravating granuloma progression and lung fibrosis. Further examining these putative interactions could more deeply inform our understanding of granuloma biology and suggest promising targets for novel TB HDTs.

Previously, limited knowledge on matrix turnover mechanisms has hindered the development of clinical strategies for managing PTLD (van Kampen et al., 2018); here, our study identifies potential cell targets, including heterogeneous fibroblast subsets such as those expressing a myofibroblast-like gene module. Lung myofibroblasts are thought to arise from a variety of routes, ranging from differentiation of tissue-resident fibroblasts, EMT (Willis et al., 2006), endothelial-to-mesenchymal transition (Piera-Velazquez et al., 2011), and bone marrow–derived progenitors such as fibrocytes (Mori et al., 2005). The myofibroblast-like cells showed in this study express genes observed in immune fibroblasts (lineage^−^, CD34^−^, CD90^+^, FAP^+^, and PDPN^+^) (Nayar et al., 2019). These cells are critical for the formation of tertiary lymphoid structures, which arise in response to sustained inflammation (Gago da Graça et al., 2021) and are commonly observed in TB-infected lung tissue (Sawyer et al., 2023). Additionally, matrix remodeling driven by skewed fibroblast populations can profoundly impact the cellular niche. Changes in ECM composition can further perpetuate fibroblast reprogramming and ECM remodeling, as seen in escalating MMP1 expression (Cole et al., 2018). These findings help guide interpretation of our cell–cell interaction analyses, highlighting significant roles for ECM-related molecules.

Postprimary human TB is often paucibacillary (Hunter and Actor, 2019), and it remains puzzling how profound lung destruction is generated under such conditions. The data presented here may support a model in which fibroblast–ECM interactions exacerbate and perpetuate lung destruction in human TB and highlight the emerging immune regulatory role of fibroblasts (Davidson et al., 2021). Of note, a phase II clinical trial in patients with pulmonary TB found that 2 wk of doxycycline, an MMP inhibitor, led to significant changes in the peripheral transcriptome at 8 wk (Miow et al., 2021), demonstrating how a matrix-modulating HDT may influence the immunological trajectory of disease. Overall, our single-cell and spatial transcriptomics analyses highlight a previously overlooked role for myofibroblast-like phenotype as a likely key player in orchestrating the immune response and regulating immunopathology in TB.

Anti-inflammatory macrophages are generally enriched in TB-diseased tissue during chronic TB infection, potentially limiting immunopathology but also creating a favorable niche for *M.tb* replication (Shim et al., 2020). Here, we found that most macrophage populations were skewed in TB-diseased lung tissues compared with TB-negative tissues, with a similar trend between post-TB and current TB spatial samples. Of particular interest are *SPP1*^*+*^ macrophages, which were elevated in TB-diseased lung tissue and strongly associated with the granuloma cuff in our spatial transcriptomics and histology data. This population has not been characterized in TB lung granuloma but is emerging as an important player in tumors, IPF-diseased lung tissue, and other fibrotic conditions (Morse et al., 2019; Qi et al., 2022). The presence of SPP1^+^ macrophages in TB granuloma was further supported by granuloma RNA-seq data from human LNs and experimentally infected NHPs. Furthermore, the *SPP1*^*+*^ macrophage markers were upregulated following TST challenge, which was amplified by concurrently active TB disease, linking their induction to *M.tb* exposure. Moreover, we found evidence of cross talk between *SPP1*^*+*^ macrophages and the human TB-myofibroblast phenotype, a previously underappreciated but potentially important interaction in TB. This putative interaction is supported by histological and spatial transcriptomics data, indicating both *SPP1*^*+*^*CHI3L1*^*+*^ macrophages and *MMP1*^*+*^*CXCL5*^*+*^ fibroblasts are tightly associated with the granuloma cuff. In IPF, *SPP1*^*+*^ macrophages are highly expanded in fibrotic lesions and cross talk with myofibroblasts to drive fibrotic changes (Morse et al., 2019); in colorectal cancer, there are direct interactions between *SPP1*^*+*^ macrophages and *FAP*^*+*^ fibroblasts expressing high levels of *MMP1*/*3* (Qi et al., 2022). In addition, mechanistic work in murine models showed *SPP1*^*+*^ macrophages can directly activate myofibroblasts via SPP1 and FN1 (Hoeft et al., 2023), both of which are implicated in the *SPP1^+^* macrophage-fibroblast cross talk we found in TB lung tissues. This interaction was also linked to an immune-suppressive, pro-tumorigenic microenvironment through active ECM deposition—resembling granuloma formation in TB (Li et al., 2024). Thus, we hypothesize that the *SPP1*^*+*^ macrophages-myofibroblast axis likely plays an important role in TB granuloma biology.

While our study provides much needed information on TB-diseased human lungs, several limitations should be acknowledged. Our cohort size is modest, and substantial variability between patients and sampling location exists in both the primary resections used in the single-cell analysis and flow cytometry experiments. We attempted to address these challenges by obtaining additional samples for spatial transcriptomics and by integrating our analyses with data from relevant public datasets. However, we are still likely to have missed some biological features underlying TB pathology. In addition, further work is needed to dissect the mechanistic role of the myofibroblast-like phenotype and the interaction of the cells that express it with *SPP1*^+^ macrophages in TB immunopathology. Possible avenues include co-culture systems, conditioned media assays, or recruitment assays to determine whether and how these fibroblasts influence and are influenced by macrophage behaviors, as well as whether chemotactic interactions exist. *Ex vivo* stimulation experiments with TB antigens on isolated fibroblasts or macrophages could help establish whether TB-specific cues directly drive differentiation toward these disease phenotypes. Genetic approaches, such as targeted knockout of key genes in *MMP1*^*+*^*CXCL5*^+^ fibroblasts or genome-wide CRISPR screens in fibroblasts within animal models of TB, could clarify causal relationships between these cells and TB pathogenesis and tissue remodeling. Beyond identifying causality, studying earlier time points in TB infection will be necessary to understand disease progression and the origins of TB complications. Ultimately, an integrated spatial, temporal, single-cell resolution disease map may be required to fully understand pulmonary reprogramming due to TB and guide optimal treatment strategies that maximize bacterial clearance while minimizing or restoring post-TB lung damage.

In sum, our study demonstrates the power of single-cell profiling to help identify and spatial transcriptomics to contextualize potential drivers of immunopathology underlying lung remodeling in TB disease. Our analysis highlights specific macrophage and fibroblast populations, as well as ECM-related processes, as promising targets for novel HDTs that could complement or offer alternatives to standard antibiotic regimens.

## Materials and methods

### Human study ethics and participants

Human lung tissue was obtained from patients undergoing surgery due to TB sequelae, including, but not limited to, hemoptysis, cavitation, bronchiectasis, shrunken, or collapsed lung, at the Department of Cardiothoracic Surgery at King Dinuzulu Hospital in Durban, KwaZulu Natal, and Inkosi Albert Luthuli Central Hospital in KwaZulu-Natal. All samples were collected with approval from the Biomedical Research Ethics Committee and written informed consents obtained from all subjects (BREC no. 019/13).

### Human lung tissue preparation

scRNA-seq samples: The lung tissue was processed within 3 h of receipt as described (Ardain et al., 2019). Briefly, a piece of the lung tissue was cut for histology and placed in 4% paraformaldehyde. The remaining piece of tissue was dissected into small pieces (5 × 5 × 5 mm) and infiltrated with a collagenase (Sigma-Aldrich) and DNase 1 (Sigma-Aldrich) in RPMI (Sigma-Aldrich) with 10% FBS (Hyclone) for 30 min. Mechanical digestion at room temperature was performed using the Gentle MACS (Miltenyi Biotec), followed by agitation at 37°C for 30 min. The mechanical digestion and agitation were repeated once more, followed by filtration of the resulting cellular suspension using the 70-mm (Corning) and 40-mm (Corning) strainer, followed by the lysis of red blood cells. Cells were then stained with trypan blue (Thermo Fisher Scientific) and enumerated using an automated cell counter (Bio-Rad) or a manual counter (Kova).

Spatial transcriptomics (Visium) samples: A section of lung was cut and transferred to 10% buffered formalin to fix for 24 h, then transferred to 70% ethanol until wax embedding. The sample was then processed in a vacuum filtration processor using a xylene-free method and isopropanol as the main substitute fixative. The tissues were embedded in paraffin wax. Tissue sections (5  µm) of specimens of good quality, as determined by trained histotechnologist, were mounted on charged slides, air-dried for 30 min, then at 42°C for 3 h in a desiccator, and stored in a desiccator at room temperature until use.

### NHP study ethics and research animals

The macaques used for generating the scRNA-seq data were part of the study published by Ganchua et al., and the same ethical and maintenance procedures were followed (Ganchua et al., 2024); all experimental manipulations, protocols, and care of the animals were approved by the University of Pittsburgh School of Medicine Institutional Animal Care and Use Committee (IACUC). The protocol assurance number for our IACUC is A3187-01. Our specific protocol approval numbers for this project are 15066174 and 18124275. The IACUC adheres to national guidelines established in the Animal Welfare Act (7 U.S.C. Sections 2131–2159) and the Guide for the Care and Use of Laboratory Animals (eighth edition) as mandated by the US Public Health Service Policy.

### NHP infections and disease tracking by PET-CT

Five cynomolgus macaques (*Macaca fascicularis*, aged between 5.3 and 9.1 years), obtained from Valley Biosystems, were part of a previously published study as the “immune naïve” control group (Bromley et al., 2024; Ganchua et al., 2024). They only received a low-dose infection (7 CFU) with *M.tb* strain Erdman and were necropsied 4 wk after infection. PET-CT was performed just prior to necropsy and results were analyzed using OsiriX viewer as previously described, with a detection limit of 1 mm (White et al., 2017). The infection dose was determined by colony counts after plating an aliquot of the infection inoculum on 7H11 agar plates, which were incubating for 3 wk at 37°C/5% CO_2_.

### Necropsy protocols

Procedures carried out during necropsy have been previously described (Ganchua et al., 2024). Briefly, 1–3 days before necropsy, PET-CT scans were taken to pinpoint the location and metabolic activity (FDG activity) of granulomas. These scans served as a guide during necropsy for precise identification and excision of these samples. On the day of necropsy, macaques were sacrificed humanely by infection of sodium pentobarbital and terminally bled. Individual granulomas and uninvolved lung tissue were all excised and homogenized separately into single-cell suspensions. Homogenates were aliquoted for plating on 7H11 agar for bacterial burden, freezing for DNA extraction, and staining for flow cytometry analysis. Any remaining samples were frozen for future use.

### Human lung scRNA-seq with Seq-Well S^3^

Seq-Well S^3^ was implemented as described (Hughes et al., 2020), the single-cell suspension was diluted to 15,000 cells in 200 μl of RPMI (Sigma-Aldrich) plus 10% FBS (Hyclone) and loaded onto a polymethylsiloxane array pre-treated with the same solution for 15 min. The cells were allowed to settle into the microwells by gravity, and the array was washed with PBS (Sigma-Aldrich) and sealed with a plasma functionalized polycarbonate membrane (Sterlitech). The arrays were then sealed, followed by incubation at 37°C for 40 min, followed by a 20-min incubation in lysis buffer containing guanidium thiocyanate (Sigma-Aldrich), EDTA (Thermo Fisher Scientific), 1% betamercaptoethanol (Sigma-Aldrich), and sarkosyl (Sigma-Aldrich) at room temperature. The arrays were then transferred to a hybridization buffer containing NaCl (Thermo Fisher Scientific), MgCl_2_ (Sigma-Aldrich), PBS (Thermo Fisher Scientific), and polyethylene glycol (Sigma-Aldrich) and were gently shaken at 60 rpm for 40 min. The capture beads hybridized with released mRNA from the lysed cells were collected from the array by a series of three wash steps with wash buffer containing NaCl (Thermo Fisher Scientific), MgCl_2_(Sigma-Aldrich), Tris-HCl (Thermo Fisher Scientific), and Water (Inqaba Biotec), with centrifugation at 2,500 *g* for 5 min each iteration. The beads were resuspended in a master mix for reverse transcriptase containing Maxima H Minus Reverse Transcriptase, Maxima Buffer, dNTPs, RNAse inhibitor, a template switch oligonucleotide, and PEG for 30 min at room temperature and overnight with endto-end mixing at 52°C. This was followed by the standard exonuclease digestion and denaturation of complementary DNA (cDNA) hybridized to the bead by 5-min incubation in NaOH (Sigma-Aldrich) and washed with a solution containing Tris-HCl, EDTA, and Tween-20 (Thermo Fisher Scientific). The beads were resuspended in a master mix containing Klenow Fragment (NEB), dNTPs, PEG, and the dN-SMRT oligonucleotide, incubating for 45 min at 38°C. PCR was performed as described in the protocol, and the product was subjected to two rounds of AMPure XP SPRI (Agencourt) bead cleanup at 0.6× and 0.8× volumetric ratios sequentially. The library size was analyzed using an Agilent Tape station hsD5000 kit, ensuring that the expected product had an average size of ∼1,000 bp and the absence of primer dimers especially below 200 bp. The Qubit High Sensitivity DNA kit was used to quantify the libraries, and they were prepared for Illumina sequencing using the NextEra XT DNA Sample Preparation kit. A total of 900 pg of the different libraries were added to the tagmentation reaction. The amplified product was purified with the AMPure XP SPRI beads, and the libraries were pooled for loading onto the NovaSeq 6000 using paired-end read structure with custom read 1 primer: read 1: 20 bases, read 2: 50 bases, and read 1 index: 8 bases.

### Spatial transcriptomics with Visium and paired H&E staining

Tissue slides were baked at 60°C for 2 h and dewaxed using two xylene changes and rehydrated with descending grades of alcohol to water. They were then H&E stained and imaged as the reference image, and the same slide was then processed as per Visium version 2 chemistry protocol following the manufacturer’s recommendations (Visium Spatial Gene Expression for FFPE – Deparaffinization, H&E Staining, Imaging and Decrosslinking, document CG000409 RevD, 10x Genomics, [Sept 2023]; Visium Spatial Gene Expression for FFPE Imaging Guidelines, document CG000436 RevB, 10x Genomics, [Sept 2023]; Visium Spatial Gene Expression Reagent Kits for FFPE User Guide, document GC000407 Rev E, 10x Genomics, [Sept 2023]). The sequencing results were processed through the SpaceRanger software following manual alignment of the fiduciary frames using the 10x Loupe browser.

### NHP sample scRNA-seq with Seq-Well S^3^

scRNA-seq was performed on both uninvolved lung tissues and granuloma tissues using the Seq-Well S^3^ platform as described by Bromley et al., where the granuloma data were previously published (Bromley et al., 2024).

### NHP single-cell data alignment and analysis

The transcript reads were aligned as described by Bromley et al. (2024). Briefly, transcript reads were tagged for cell barcode and UMI using DropSeqTools version 1.12, then aligned to the *M. fascicularis* version 5 genome (https://useast.ensembl.org/Macaca_fascicularis/Info/Index) through the Dropseq-tools pipeline on the Terra platform (app.terra.bio) (Macosko et al., 2015). Aligned reads were collapsed by barcode and UMI sequences to generate digital gene expression matrices for each array, covering 10,000 barcodes. For each sample, gene expression matrices with ≥10,000 barcodes were processed through CellBender to estimate ambient RNA fraction. The “remove-background” function in CellBender was applied with default settings. Next, the matrices “corrected” by CellBender were analyzed with Scrublet, with default parameters to detect potential doublets. Any transcriptome with a doublet_score > 0.30 was removed from downstream analyses.

After that, the gene expression matrices for each sample were merged and processed in Scanpy (version 1.8.2). Transcriptomes were filtered using the following criteria: min_genes >300, min_counts > 500, mitochondrial_threshold = 0.05, and genes expressed in at least 10 cells. Gene expression counts were normalized using default Scanpy parameters (i.e., log2(TP10K+1)). Coarse-level cell type clustering and iterative subclustering were used to annotate cell types and further detect low-quality transcriptomes (e.g., doublets). Cell types were identified using canonical markers, and only fibroblast cells were included in the analysis presented in this study.

### Human lung single-cell data analysis and cell type identification

The raw sequencing reads from the NovaSeq run were aligned to the hg19 genome assembly and processed in accordance with the Drop-Seq Computational Protocol version 2.0 (https://github.com/broadinstitute/Drop-seq). The output (cell by gene matrix) was then loaded to the Seurat R package version 3.1.0 (https://satijalab.org/seurat/), transformed to log_e_(UMI +1), and followed by scaling by a factor of 10,000. The overall quality was assessed by the distribution of reads, transcripts, and genes per cell (percentage of mitochondrial genes <5, nFeature_RNA<2500, nFeature_RNA>200, and nCount_RNA>200). SCTransform by Seurat was called to perform normalization of the gene counts, selecting top 3,000 highly variable genes, and scaling normalized gene counts. Principal component analysis was run on the selected highly variable genes to give the top 50 PCs. A custom elbow-based method was used to find the smallest number of PCs (n_pcs) where the eigengap between two adjacent PCs drops below 20 percentile of all eigengaps among top 50 PCs. Uniform Manifold Approximation and Projection (UMAP) was calculated using the RunUMAP function, and neighborhood graph was calculated by FindNeighbors, both using reduction *=* “pca” and selecting top n_pcs as input dimensions. Unsupervised Louvain clustering using the FindClusters was used to identify transcriptionally similar cells with parameters assay *=* “integrated”, dims.use = n_pcs, k.param *=* ceiling(0.5*sqrt(#cells)), and we performed a resolution scan for the best clustering resolution from 0.2 to 2 while optimizing for silhouette score. Cell type annotation were done by cross-referencing canonical cluster defining genes with well-curated lists and online databases such SaVant T (http://newpathways.mcdb.ucla.edu/savant-dev/) and GSEA/MsigDB (https://www.gsea-msigdb.org/gsea/msigdb/index.jsp). Doublet clusters where multiple canonical markers were expressed are identified and removed, and the entire dataset are reprocessed starting from the SCtransform step. Final differentially expressed (DE) gene for each of the major clusters were found by calling FindAllMarkers from Seurat using default setting and adjusted P value cutoff <0.05, and top DE genes were found by ranking log fold-change values from high to low. Heatmap of DE genes were plotted using Seurat function DoHeatmap, and dotplot was achieved using function DotPlot.

Subclustering for major cell groups (macrophage/monocytes, neutrophils, epithelial cells, and fibroblasts) were performed similarly to the entire dataset after subsetting to the specific cell types. Marker genes for each subcluster was found by calling FindAllMarkers from Seurat using default setting, and significant genes (adjusted P value < 0.05) are visualized with custom volcano plots.

Comparison with human LN data was done for the top 10 DE genes in each cellular subcluster and checked over the TB vs. control differential testing result from the human LN granuloma study.

### Clustering analysis on cell subtypes

Proportion of cell subtypes in each patient was calculated, and Pearson’s correlations between every pair of broad level cell type are calculated. For each pair of cell types, we ran permutation test by randomly reassigning cell type labels to generate a set of background correlation values, and P values are calculated as the percentage of the permutated correlation values exceed the original observation. Hierarchical clustering on the cell types are done by feeding in the pairwise correlation into Python function linkage with method = “average,” metric = “correlation”; we then use function fcluster with a defined k to call cluster from the returned linkage result with criterion = “maxclust”. We grid searched through k from 2 to 29 (one less than the number of cell types) and determined the optimal cluster number by computing the silhouette score from each clustering result with function silhouette_score and a precomputed correlation distance. This allowed us to select k = 12, which resulted in the highest silhouette score. For each of the 12 clusters identified through hierarchical clustering, we further calculated permutation test P values to examine average correlation values within and outside of each cluster and annotate those that has within-group P value < 0.05.

### Differential abundance testing

Statistical differences in the cell type abundance between TB-diseased and TB-negative lungs were tested by two-sided Fisher’s exact test at the cell level and adjusted for multiple testing correction by Holm’s method.

Cluster-free differential abundance testing is done using milopy in Python. Neighborhoods are constructed over the entire dataset using $k=ceil\left(0.5\times \sqrt{n}\right)$, where *ceil* rounds up to the nearest integer and *n* is the number of cells. Neighborhoods are made with *prop* = 1. Function DA_nhoods were called with design=∼HIV+TB to account for the effect of HIV status. For interpretation, we only kept neighborhoods with neighborhood annotation fraction > 0.5 and labeled them with the majority cells’ annotation. Due to the small sample size, we opted to use P value instead of the spatial false discovery rate (FDR) devised in milopy for significance.

### Bulk RNA-seq profile deconvolution and comparison

For comparing the marker genes in each subcluster with DE genes in bulk RNA-seq on human LN TB granuloma samples, we first selected top 50 DE genes in each subcluster. Note that some of the DE genes in a broad cell type may overlap with the DE genes in another, since the differential analysis was done within each broad cell type. Hence, we remove the genes that are shared between cell types, re-ranked the remaining DE genes by log-fold change, and took the top 10 DE genes to compare with the bulk differential expression results.

For deconvolution of the human LN TB granuloma and control samples, we applied tool MuSiC (1.0.0) separately on TB and control samples, using annotated data in our study as single-cell reference. We kept all the cell types for deconvolution except alveolar macrophages, which should only exist in lungs. Other parameters are kept as default.

We applied a standard two-sided *T* test to compare the difference between inferred cell type proportions between TB and control LN samples, with Bonferroni correction for multiple testing.

### Fibroblast label transfer and gene signature finding

#### Travaglini et al. (2020) stromal cell type calling

Top 20 markers for each stromal cell population by Travaglini et al. (2020) (Table S4) were found by filtering on P value < 0.05 and sorted by average log fold-change. AddModuleScore from Seurat was used to calculate module score of these markers, and “Travaglini.fib.subtype” was called based on which cell type gives the maximum module score, where “ambiguous” was assigned if no score gives a positive value. Proportion of Travaglini.fib.subtype was calculated in each fibroblast cluster given this new cell annotation.

#### HLCA label transfer

HLCA label transfer onto our dataset was achieved following their tutorial (https://github.com/theislab/scarches/blob/hlca_tutorial_improvements/notebooks/hlca_map_classify.ipynb). Briefly, label transfer was done using asArches on the raw counts of the entire dataset on the genes that are part of the reference model. Annotation level 3 data were used in this paper. Celled called as “fibroblast” or “myofibroblast” are considered together as fibroblast population, which are highly consistent with our manual annotation (>95% true positive rate). For better comparison, we only included HLCA fibroblasts (and myofibroblast) with tissue source annotation “lung parenchyma.” Differential gene expression analysis was performed between all TB-negative controls (from both HLCA and our study) and TB-diseased lungs (only from our study) on log normalized counts. GSEA was run in R using gene sets from MSigDB (accessed using msigdbr) on DE genes passing filter for Benjamin–Hochberg adjusted P value < 0.05.

#### Gene module finding with hdWGCNA

Single-cell version of WGCNA was run using tool hdWGCNA following tutorial (https://smorabit.github.io/hdWGCNA/articles/basic_tutorial.html). Briefly, gene_select = “variable” was used to set the variable gene selection approach using SetupForWGCNA. To avoid sparsity in the single-cell data, we first constructed metacells that aggregates the expression profile based on neighborhood information. Metacells were constructed through MetacellsByGroup call with parameters k = 10, max_shared = 5, and min_cells = 20; group.by uses the categories for fibroblast subcluster and disease status (TB, HIVTB, HIV control, and cancer control), and ident.group is also set to be the subcluster. SetDatExpr was called with “SCT” assay and “data” slot for scaled expression. TestSoftPowers function was called with networkType = “signed”. The rest follows the default analysis workflow. Top genes in each module ranked by eigen-based connectivity (kME) are visualized by running PlotkMEs. Feature plot of module eigengenes (MEs) for each module was plotted by running ModuleFeaturePlot with features = “MEs”. ModuleCorrelogram function was used to visualize the correlation between each module based on their MEs, and VlnPlot from Seurat was used to visualize the difference of module MEs between subclusters.

### LN granuloma laser capture microdissection RNA-seq study

FFPE clinical samples from 24 adult patients undergoing mediastinal or neck LN biopsy were selected (seven TB, 10 sarcoidosis, and seven normal), and the first analysis has previously been reported (Reichmann et al., 2014; GEO accession code GSE174443). The patients were treatment naïve and had no significant comorbidities, were HIV negative, and were nonsmokers. Sections of 10 μm thickness were cut, floated in RNase-free water, mounted on to polyethylene naphthalate membrane glass slides, and dried at 37°C overnight. Sections were dewaxed with xylene immersion followed by xylene removal with 100% EtOH. Laser capture microdissection was used to isolate granulomas or similar area of control normal tissue. Each sample underwent total RNA extraction and sequenced using Ion Torrent sequencing. Raw sequencing data were aligned using Kallisto software and annotated to gene level by ensembldb, and sleuth programs to ensure similar results were found. Inter-sample normalization was performed using TMM normalization (edgeR).

### Evaluation of differential genes in LN granuloma dataset

Genes identified from each cluster during single-cell sequencing analysis were searched within the bulk RNA-seq dataset of granulomas isolated by laser capture microdissection (GEO accession code GSE174443), where differential gene expression analysis was performed using limma with its voomWithQualityWeights function (version 3.38.3, R) with Benjamini–Hochberg FDR of < 0.05. Filter values were optimized to yield the highest number of differentially expressed genes across the study cohort. GraphPad Prism 9 was used to plot the average gene expression of seven control and seven TB LNs, with box-and-whisker values generated using one-tailed unpaired *T* test.

### Evaluate gene module in NHP dataset

Gene modules found from above are taken to be evaluated in NHP data. Top 50 hub genes are ranked by eigengene-based connectivity (kME) and used to score on fibroblasts from the NHP dataset using function score_genes from Python package scanpy. Two sided Mann–Whitney U test without correction was used to compare module usage between different conditions.

### Evaluate gene modules in human TST challenge dataset

Top 50 hub genes from the Fibroblast-M1 module from hdWGCNA are taken as the human TB-myofibroblast module as described above, along with differentially expressed marker genes from *SPP1*^+^*CHI3L1*^+^ macrophages (Data S8), they are used to score on the bulk RNA-seq data which has been preprocessed following methods in Pollara et al. followed by calculating geometric means of all the genes in set (Pollara et al., 2021). Two sided Mann–Whitney U test without correction was used to compare module usage between different conditions.

### Cell–cell interaction analyses

#### MultiNicheNet

Analysis was run using package multinichenetr following tutorials on https://github.com/saeyslab/multinichenetr. Briefly, recommended ligand–receptor network and ligand-target matrix were downloaded from https://zenodo.org/record/7074291/files, and a SingleCellExperiment object was constructed from the RNA assay from the Seurat object. Analysis was defined for senders and receivers as all broad-level cell types shown in Fig. 1. We performed genome-wide differential expression analysis of receiver and sender cell types to define DE genes between the conditions of interest (TB-negative and TB-diseased lungs). Empirical P values were calculated after differential expression calculation using function get_empirical_pvals. Then, we predicted NicheNet ligand activities and NicheNet ligand-target links based on calculated differential expression results using function get_ligand_activities_targets_DEgenes with parameters logFC_threshold = 0.50, p_val_threshold = 0.05, fraction_cutoff = 0.05, p_val_adj = FALSE, and top_n_target = 250. We see the information collected above to prioritize all sender-ligand-receiver-receptor pairs using function generate_prioritization_tables with prioritizing weights: “de_ligand” = 1, de_receptor” = 1, “activity_scaled” = 2, “exprs_ligand” = 2, “exprs_receptor” = 2, “frac_exprs_ligand_receptor” = 1, “abund_sender” = 0, “abund_receiver” = 0, and fraction_cutoff = 0.05; grouping_tbl consists of sample ID and TB status. Circoplot visualizations of top 20 ligand–receptor pairs in each TB status group were done on prioritization table outputs. Summary heatmap was done over top 200 interactions for enrichment of interactions between cell types.

Given the requirement to perform genome-wide differential expression analysis to identify DE genes between TB conditions, we could not apply the same MultiNicheNet framework to all subclusters, given some subclusters do not have enough power to detect. Hence, we switched to LIANA for an unbiased cell–cell communication analysis at the subcluster level.

#### LIANA

LIANA analysis was first independently run on both TB-diseased data and healthy control data using function liana_wrap, followed by liana_aggregate from the LIANA package in R using default parameters on RNA assay from Seurat. We kept only interactions concordant between methods by filtering for interactions with aggregate_rank ≤0.01. Top 20 MMP1^+^ CXCL5^+^ fibroblast-specific signaling in TB was extracted, where interaction specificities are extracted from natmi.edge_specificity values and expression magnitudes are from sca.LRscore value between interactions. Senders/receivers are ordered by hierarchical clustering based on Pearson’s correlation of sca.LRscore values.

We summarized the sender–receiver interaction frequencies from the filtered interactions in each TB status group and calculated the difference between the two frequency matrices. Lastly, we normalized by the largest absolute value of differences for plotting the interaction difference heatmap. To visualize interactions strengthened in TB-diseased group and TB-negative group, we defined the edge weight of interactions by the natmi.edge_specificity from LIANA output and edge_FC as the fold change between the TB group and control group with a pseudo edge weight of 10^−6^ if control group is 0. We counted the number of interactions between sender–receiver groups involved in interactions of edge_FC > 1, defined as “poslogFC.cellcell.count” and similarly the number of interactions between sender–receiver groups involved in interactions of edge_FC < 1, defined as “neglogFC.cellcell.count.” We clustered sender and receiver in TB-upregulated interactions (summarized in “poslogFC.cellcell.count”) and TB-downregulated interactions (summarized in neglogFC.cellcell.count) based on Pearson’s correlation of interaction count similarities between senders and receivers, respectively. Hierarchical clustering was done using pheatmap followed by inspecting tree clusters and calling groups using cutree. For circos plots of TB-upregulated sender–receiver pairs and TB-downregulated sender–receiver pairs, we only selected for pairs with interaction counts exceeding 80 percentile of all pairs in the particular condition, using function chordDiagram from R package circlize.

For visualizing the interactions between fibroblast and macrophages and *SPP1*^*+*^ macrophage signaling, we visualized the count of interactions with aggregate_rank ≤ 0.01 and edge_FC > 1 and used chordDiagram to plot.

#### Ligand interaction strength calculation

Mean TB edge is defined as the mean of interaction edge weight in TB-diseased group for specific sender and ligand combination, and mean_CTR_edge is defined as the mean of interaction edge weight in TB-negative group. weighted_mean_TB is defined as mean_TB_edge weighted by the count of interaction involving that ligand in each sender group, and similarly for weighted_mean_CTR. Finally, weighted_mean_FC (e.g., interaction strength change) is defined as weighted_mean_TB/weighted_mean_CTR. Top 30 and bottom 30 ligands by the interaction strength are chosen to be visualized in Fig. 5 D.

We also calculate an unweighted mean_FC = mean TB edge/mean_CTR_edge for the interaction strength change in each sender cell type, and we consider an interaction involving a ligand as positive if the log10(mean_FC) is positive and negative if the log10(mean_FC) is negative, which reflects whether the interaction is stronger or weaker in TB-diseased vs. TB-negative group. Positive and negative interaction counts are tallied for each ligand, and a ligand is thought to be dominantly “positive” (colored red in barplot) if positive interaction count is 50% higher than negative interaction count and “negative” (colored blue) if negative interaction count is 50% higher than positive interaction count. Mean_FC and log10(mean_FC) between TB-diseased and TB-negative samples are used to indicate ligand activity importance in each sender cell type; sender cell types are grouped according to clustering for TB-diseased senders in circos plot. Grouping of sender cell types in the ligand interaction strength analysis was the same as before. Top five ligands in *MMP1*^*+*^*CXCL5*^*+*^ fibroblast by mean TB edge metric was visualized for their proposed targets and number of possible receptor interactions on each cell type.

For summarizing top 10% of ligands in each TB condition, we calculate the mean of edge_FC for each source/ligand combination. The mean edge_FC is then sorted by descending order, where the top 10% and bottom 10% are visualized as top ligands upregulated in TB (Data S4, B and C). For the barplot of number of interactions upregulated in each TB condition, we filter for all interactions with edge_FC < 1 or edge_FC > 1 and count the number of interactions by sender cell type. We use the same cluster groupings/colors for the senders as for the circus plot in Fig. 4 D.

#### Sender signaling co-occurrence analysis

We first filter out sender-ligand combinations that are upregulated in TB (edge_FC > 1). Then, for each cell type of interest, the normalized RNA count for the upregulated ligands in this cell type is retrieved for all the TB-diseased patients. The ligand expression in each cell is then weighted by log10(mean_FC), so ligands with larger degree of change are weighted higher for their expressions. Then, patient averages of all the weighted ligand expressions are calculated and summed to arrive at a final patient-sender activity score. Pearson’s correlation is computed across each pair of cell type’s sender activity scores in nine patients.

#### Interaction flux analysis

In this analysis, we define the flux of interaction in the direction from sender to receiver cell types. First, we calculate the mean of edge_FC between all LIANA inferred significant interactions (aggregate_rank ≤ 0.01) for each sender–receiver pair. Then, for each pair of sender–receiver, the flux of interaction is calculated by multiplying the sender cell count. The total flux of a given sender cell type is then the sum of flux to all receiver cell types.

### Fluorescent immunohistochemistry staining

#### Sample preparation

Multiplex fluorescent immunohistochemistry staining of macrophage markers was performed on lung tissue sections using the Opal 6-Plex Manual Detection Kit 50 Slides (Akoya) as directed by the manufacturers. Multiplex fluorescent immunohistochemistry staining of fibroblast markers was performed on lung tissue sections using the Opal 4-Color Manual IHC Kit 50 Slides (PerkinElmer) as directed by the manufacturers. For both, lung tissue samples fixed in 4% formalin were paraffin embedded. Four mm sections were cut on X-tra adhesive precleaned micro slides (Leica), allowed to dry for a minimum of 24 h, and the slides were baked at 60°C overnight.

#### Deparaffinization, rehydration, and antigen retrieval

The combined process of deparaffinization, rehydration, and antigen retrieval of the tissue sections was done using 1× Envision Target Retrieval Solution, High PH (Dako) in the PT-Link Pre-Treatment Instrument (Dako). Thereafter, slides were incubated for 1 min in distilled water and equilibrated in EnVision FLEX Wash Buffer (Dako) at room temperature for 10 min (2 × 5 min using fresh buffer for each period) for macrophage markers staining and 5 min for fibroblast markers staining. Then, the macrophage slides were incubated in EnVision FLEX Peroxidase blocking solution (Dako) for 10 min, and fibroblast slides were incubated in Peroxidase blocking solution (PerkinElmer) for 10 min; both were then washed in wash buffer (Dako) as before immediately at room temperature.

#### Background reduction

The macrophage slides were incubated in blocking buffer (0.05 g BSA +10% goat serum dissolved in EnVision FLEX Wash Buffer) for 20 min. The fibroblast slides were incubated in Bloxall blocking solution (PerkinElmer) for 10 min.

Antibody staining: The macrophage slides were incubated in primary antibody-1 for 45 min, fibroblast slides for 30 min, at room temperature, then washed for 5 min in wash buffer. The macrophage slides were then incubated in Secondary Opal Polymer Horseradish Peroxidase (HRP) Mouse and Rabbit (Akoya) for 20 min, and fibroblast slides were incubated in Secondary Opal Polymer Horseradish Peroxidase (HRP) Mouse and Rabbit (PerkinElmer) for 30 min. Then, the slides were washed twice in wash buffer as before, drained, and the sections were incubated in Opal Polymer Fluorophore (macrophage slides: Akoya; fibroblast slides: PerkinElmer) working solution for signal amplification at room temperature for 10 min in the dark. The slides were then washed for 10 min (2 × 5 min using fresh buffer for each time) for macrophage slides, 5 min for fibroblast slides, in wash buffer at room temperature.

#### Antibody stripping

Afterward, the antigen retrieval via microwave treatment was done by placing the slides in a slide jar with pre-warmed buffer AR6 (macrophage slides: Akoya; fibroblast slides: PerkinElmer). The jar was loosely covered and placed in a microwave for 2 min at 100% power (high setting), 10 min at 50% (medium setting) power, and 5 min at 20% (low setting) power. Slides were cooled down in the dark by placing the slide jar on ice for 20 min, and the slides were rinsed in distilled water, followed by incubation in the wash buffer for 10 (2 × 5 min) minutes for macrophage slides and 5 min for fibroblast slides to equilibrate slides. The microwave step re-exposes the antigen on the tissue and allows the introduction of the next primary antibody. For the detection of the next target (primary antibody 2), the protocol was restarted at the blocking step using blocking buffer (macrophage slides: 0.05 g BSA +10% goat serum dissolved in EnVision FLEX Wash Buffer; fibroblast slides: Bloxall blocking solution from PerkinElmer) for 10 min. After the third target was detected (primary antibody 3), a working solution of DAPI (macrophage slides: Akoya; fibroblast slides: PerkinElmer) was applied to the sections as the nuclear counterstain for 5 min in a humidity chamber. The slides were washed in wash buffer for 5 min, then in distilled water for 5 min, and drained. Then, the sections were coverslip with Fluorescence Mounting Medium (Agilent Technologies, Inc.), and the edges of the coverslip were sealed with nail varnish. Slides were stored in a humidity chamber at 4°C until images are acquired.

#### Antibodies and fluorophores

For macrophage slides, the unconjugated primary antibodies used are Anti-CD68 (conc. clone: Ab213363; Abcam), Anti-CD206 (clone: Abcam), and Anti-Osteopontin (clone: ab302942; Abcam). The primary antibodies were diluted in antibody diluent (PerkinElmer) as recommended by the antibody manufacturer, and the Opal fluorophores were diluted in amplification diluent (PerkinElmer). The fluorophores used for signal generation in this study are FITC, tetramethylrhodamine, and Cy5. For fibroblast slides, the unconjugated primary antibodies used are Anti-Collagen I (clone: ab34710; Abcam), Anti- Anti-CTHRC1 (clone: ab85739; Abcam), Anti-TDO2 (clone: OT14G2; Thermo Fisher Scientific), Anti-PI15 (clone: PA5-52312; Thermo Fisher Scientific), and Anti-ACTA2 (clone: 1A4; LSBio). The primary antibodies were diluted in antibody diluent (PerkinElmer) as recommended by the antibody manufacturer, and the Opal fluorophores were diluted in amplification diluent (PerkinElmer). The fluorophores used for signal generation in this study are FITC, Texas-Red, and Cy5.

#### Imaging

For macrophage slides, the images were acquired on Hamamatsu NanoZoomer S60, and analyzed with NDP.view2 (version 2.9.29) imaging software (TissueGnostics). For fibroblast slides, the images were acquired on a Zeiss Axio Observer Z1 inverted microscope (Olympus) and analyzed with TissueFAXS imaging software (TissueGnostics).

#### Quantification

For macrophage slides, using QuPath software (version 0.5.0-x64), TB granulomas were segmented into three distinct layers:

1. An outer cellular layer primarily composed of lymphocytes,
2. An inner cellular layer predominantly consisting of myeloid cells (mainly macrophages),
3. A necrotic core characterized by cellular debris and dead cells.

To assess the expression levels of SPP1 (green) and CD68 (yellow), we divided the granuloma radially similar to pie-cutting, into 10 regions, which further divides each granuloma layer into 10 subregions. Each subregion is defined and analyzed as a separate region of interest (ROI), where they are numbered clockwise from 1 to 10, so ROI1 from the necrotic core is immediately adjacent to ROI1 from the inner cellular layer, etc. This enabled a more precise evaluation of spatial differences in protein expression. Expression intensity was measured at 5 µm per pixel to capture variability in protein distribution across the granuloma architecture.

Mean intensity for each ROI was used to calculate the statistical significance between the groups using two-sided Mann–Whitney U test without correction for SPP1. The ratio of mean intensity between inner cellular layer and outer cellular layer was calculated between the ROI1 and ROI1, ROI2 and ROI2, etc. The same was done for the ratio of mean intensity between inner cellular layer and the necrotic core.

### Flow cytometry

Lung pieces collected after removal from *M.tb*-infected patients were used in flow cytometry analysis after processing as from scRNA-seq (Table S4). Cells were counted and stained with antibody cocktail for 30 min at room temperature and in the dark, followed by 2× wash steps with PBS and resuspension of stained cells in FACSLyse. The surface markers used were CD45 (CD45-APC, cat#304012; BioLegend), CD34 (CD34-FITC, cat#324226; BioLegend), EpCAM (EpCAM-BV650, cat#324204; BioLegend), CD11b (CD11b-PeCy7, cat#557743; BD), CD31 (CD31-BV605, cat#303121; BioLegend), VCAM1 (VCAM-PE, cat#305805; BioLegend), ICAM1 (ICAM-APC-Cy7, cat#353121; BioLegend), podoplanin (PDPN-PERCPefluor710, cat#46-9381-42; Thermo Fisher Scientific), and CD235a (CD235a-PECF594, cat#349119; BioLegend). Viability was determined using the Invitrogen Live/Dead Aqua Fluorescent reactive dye on the HV500 channel. Samples were acquired on the BDFACS Aria Fusion flow cytometer. Analysis of samples was subsequently carried out using FlowJo (version 10, FlowJo).

The Friedman test was used to assess significant changes in the fibroblast subset of interest across different lung resection severities.

### Human lung tissue spatial transcriptomics data analysis

Filtered 10x spatial data from each sample processed by Space Ranger was read into an anndata object using the function “read_visium” from Python package scanpy, along with the corresponding high-resolution image of the H&E stain. No filtering on spots or genes was done to preserve the maximum amount of information as the nature of these data are intrinsically sparse. Data were log-normalized with standard scanpy workflow. Top 50 hub genes from the hdWGCNA fibroblast-M1 module were used to score for human TB-myofibroblast signature on each Visium spot using score_genes from scanpy. The top 20 differentially expressed markers from the macrophage subsets (Data S8) were used to calculate DE marker scores in a similar fashion. Spearman’s correlation and its significance between the human TB-myofibroblast signature and macrophage subset markers were calculated using the function spearmanr from Python package scipy. A two-sided Mann–Whitney U test without correction was used to compare module usage between different conditions, and Spearman’s correlation was used to compare human TB-myofibroblast and different macrophage subsets.

### Deconvolution of spatial transcriptomic data

Since Visium version 2 chemistry has spot size of diameter = 55 μm (generally larger than one cell), we estimated the cell type abundance of each spot using Python package cell2location, a Bayesian model that estimates the combination and abundance of cell types that could give rise to the mRNA counts in each spatial location. We first learned reference signatures of each broad-level cell type from the original scRNA-seq cohort generated using Seq-Well S^3^, then decomposed the Visium multi-cell RNA counts into these reference signatures, establishing a spatial mapping of cell types. For training the reference signatures, we used patient ID as categorical_covariate_keys and sequencing batch as batch_key*,* num_samples *= *1,000, batch_size *= *2,500, and batch_size *= *250, with the rest set to default. For the posterior estimating, we created and trained the model with hyperparameters: N_cells_per_location = 10, detection_alpha = 20, and max_epoches = 15,000, with the rest set to default. For each boxplot comparing cell type abundance, 5% quantile of the estimated posterior was used to represent cell type abundance at each Visium spot, which represents the value of abundance the model has high confidence in.

### Annotation of granuloma structures on H&E images

Granuloma structures were manually annotated using ImageJ by experts in TB lung pathology. A band of ∼20-pixel width was then drawn outside the selection area to approximate the granuloma cuff. The spots from the Visium data are categorized to be “in,” “on,” and “out” of the granuloma border based on the spot’s corresponding position relative to this segmentation band.

### Spatial ligand–receptor analysis

Each sample was log-normalized with the scanpy package. The Python version of LIANA package was then used to impute spatial ligand–receptor interactions. First, spatial neighborhoods were calculated using the spatial_neighbors with bandwidth *= *10, cutoff *= *0.1, kernel *=* “gaussian,” and set_diag *=* True. Then bivariate scores for potential ligand–receptor pairs is calculated using the function bivariate using with parameters bivariate = “consensus,” local_name *=* “cosine” (spatially-weighted cosine similarity for local score), global_name *=* “morans” (bivariate Moran’s R for global score), n_perms *= *100, nz_prop *= *0.05, and default settings for the rest. Top L–R pairs are selected by sorting for descending Moran’s R as it describes global co-expression.

### Online supplemental material

Fig. S1 shows spatial transcriptomics on TB-infected human lung samples and single-cell deconvolution. Fig. S2 shows single-cell transcriptomic reveals heterogeneity within neutrophil populations with disease-specific difference. Fig. S3 shows deconvolution of bulk human LN dataset and fibroblast in spatial and single-cell dataset. Fig. S4 shows fibroblast subclusters’ marker genes, WGCNA, and comparisons against public datasets. Fig. S5 shows SPP1^+^ macrophage interaction with fibroblasts in TB. Data S1 shows cell type annotation and epithelial subclustering. Data S2 shows expression of marker genes from monocyte/macrophage subclusters and differential abundance testing. Data S3 shows cell–cell interaction analysis by TB conditions. Data S4 shows cell–cell interaction changes between TB conditions and AT1 cell sender activities. Data S5 shows expression of hypothesized secreted ligands by MMP1^+^CXCL5^+^ fibroblast in human TB LN granuloma vs. healthy LN. Data S6 shows spatial transcriptomics analysis on post- and current TB lung resections. Data S7 lists clinical metadata on Visium spatial transcriptomic samples. Data S8 lists subcluster DE genes, GSEA, and Enrichr results on these DE genes. Data S9 lists the top 10 markers of cell subclusters that overlap with human LN TB granuloma bulk dataset of differentially expressed genes by Reichmann et al. (2021). Data S10 shows overlap between macrophage/monocyte subcluster marker genes with NHP macrophage marker genes from Gideon et al. Data S11 shows fibroblast WGCNA of top 50 hub genes in each module. Table S1 shows metadata on scRNA-seq patient cohort in this study; Table S2 shows patient-level broad cell type representation; Table S3 shows metadata on 4-wk postinfection NHP cohort used in Fig. 5 E. Table S4 shows metadata on *M.tb*-infected patients used for flow cytometry. Table S5 shows sample count for Visium spatial transcriptomic data.

## Supplementary Material

Table S1 shows metadata on scRNA-seq patient cohort in this study.

Table S2 shows patient-level broad cell type representation.

Table S3 shows metadata on 4-wk postinfection NHP cohort.

Table S4 shows metadata on *M.tb*-infected patients used for flow cytometry.

Table S5 shows sample count for Visium data.

Data S1 shows cell type annotation and epithelial subclustering.

Data S2 shows expression of marker genes from monocyte/macrophage subclusters and differential abundance testing.

Data S3 shows cell–cell interaction analysis by TB conditions.

Data S4 shows cell–cell interaction changes between TB conditions and AT1 cell sender activities.

Data S5 shows expression of hypothesized secreted ligands by MMP1^+^CXCL5^+^ fibroblast in human TB LN granuloma vs. healthy LN.

Data S6 shows spatial transcriptomics analysis on post- and current TB lung resections.

Data S7 shows clinical metadata on Visium spatial transcriptomic samples.

Data S8 shows subcluster DE genes, GSEA, and Enrichr results on these DE genes. Enrichr result on WGCNA module genes.

Data S9 shows the top 10 markers of cell subclusters that overlap with human LN TB granuloma bulk dataset of differentially expressed genes by Reichmann et al. (2021).

Data S10 shows overlap between macrophage/monocyte subcluster marker genes with NHP macrophage marker genes by Gideon et al.

Data S11 shows fibroblast WGCNA of top 50 hub genes in each module.

## Data Availability

The raw and analyzed scRNA-seq and spatial data from this study have been deposited in the Broad Institute Single Cell Portal at https://singlecell.broadinstitute.org/single_cell/study/SCP3227/single-cell-and-spatial-profiling-reveals-a-role-for-tuberculosis-induced-myofibroblasts-in-the-immunopathology-of-infected-lungs. The 4-wk postinfection NHP were previously used in another study by Ganchua et al. (2024) and Bromley et al. (2024). The other 4-wk p.i. and 10-wk p.i. NHP granuloma dataset by Gideon et al. can be accessed from GEO with accession number GSE200151. The HLCA can be accessed at https://data.humancellatlas.org/hca-bio-networks/lung/atlases/lung-v1-0. The human TB LN bulk RNA-seq data by Reichmann et al. (2021) can be accessed on GEO with accession number GSE174443. Any additional information required to reanalyze the data from this study is available from the lead contact upon request.
