# Correction: Single-cell and spatial profiling highlights TB-induced myofibroblasts as drivers of lung pathology

**DOI:** 10.1084/jem.2025106701072026c

**Published:** 2026-01-14

**Authors:** Ian M. Mbano, Nuo Liu, Marc H. Wadsworth, Mark J. Chambers, Thabo Mpotje, Osaretin E. Asowata, Sarah K. Nyquist, Kievershen Nargan, Duran Ramsuran, Farina Karim, Travis K. Hughes, Joshua D. Bromley, Robert Krause, Threnesan Naidoo, Liku B. Tezera, Michaela T. Reichmann, Sharie Keanne Ganchua, Henrik N. Kløverpris, Kaylesh J. Dullabh, Rajhmun Madansein, Sergio Triana, Adrie J.C. Steyn, Bonnie Berger, Mohlopheni J. Marakalala, Gabriele Pollara, Sarah M. Fortune, JoAnne L. Flynn, Paul T. Elkington, Alex K. Shalek, Alasdair Leslie

Vol. 223, No. 3 | https://doi.org/10.1084/jem.20251067 | January 5, 2026

The authors regret that Gabriele Pollara was inadvertently omitted from the author list in the original version of their article. The corrected author list appears above. The error appears in PDFs downloaded before January 12, 2026.

