# Supplementary material for: Single-cell and spatial profiling highlights TB-induced myofibroblasts as drivers of lung pathology
Source: J Exp Med. 2026 Jan 5;223(3):e20251067. doi: 10.1084/jem.20251067 (PMC12767585; doi:10.1084/jem.20251067)
Supplement: Table S1 — shows metadata on scRNA-seq patient cohort in this study. [file jem_20251067_tables1.docx]

**Table S1. Metadata on scRNA-seq patient cohort in this study**

| **Patient** | **Sex** | **Age** | **Previous TB** | **HIV status** | **Pulmonary TB complication** | **Lung tissue collected** | **Cell yield** |
| --- | --- | --- | --- | --- | --- | --- | --- |
| P0 | Male | 37 | 2020 | Positive | Cavitation and Fibrosis | Right upper lobectomy | 1637 |
| P4 | Female | 49 | 2013 | Positive | Cavitation, Bronchiectasis, Nodules | Left Pneumonectomy | 5296 |
| P6 | Female | 21 | 2017 | Negative | Cavitation, Haemoptysis, Dyspnoea, Mycetoma | Right Pneumonectomy | 4985 |
| P8 | Female | 58 | 2017 | Negative | Cavitation, Haemoptysis | Left Pneumonectomy | 2159 |
| P9 | Female | 56 | none | Negative | n/a | Right VATS biopsy | 1268 |
| P10 | Female | 61 | none | Negative | n/a | Right VATS biopsy | 611 |
| P11 | Female | 32 | 2013 | Positive | Minor Haemoptysis | Left Pneumonectomy | 298 |
| P36 | Male | 41 | 2019 | Positive | Massive Haemoptysis | Left Pneumonectomy | 1036 |
| P37 | Male | 53 | 2019 | Positive | Left chest pain | Left Bullemctomy | 573 |
| P38 | Female | 58 | 2019 | Positive | Massive Haemoptysis | Left Pneumonectomy | 521 |
| P41 | Male | 45 | none | Negative | n/a | Left upper lobectomy | 291 |
| P43 | Female | 44 | 2019 | Positive | Cavitation, Haemoptysis | Right Pneumonectomy | 369 |
| P44 | Female | 36 | none | Positive | n/a | Right VATS biopsy | 588 |
| - VATs - video assisted thorascopic surgery - The patients are color coded according to group - HIV+TB+ (blue), TB+ (purple), HIV-non-TB control (red), HIV+ non-TB control (green) | | | | | | | |
