# Supplementary material for: Single-cell and spatial profiling highlights TB-induced myofibroblasts as drivers of lung pathology
Source: J Exp Med. 2026 Jan 5;223(3):e20251067. doi: 10.1084/jem.20251067 (PMC12767585; doi:10.1084/jem.20251067)
Supplement: Table S2 — shows patient-level broad cell type representation. [file jem_20251067_tables2.docx]

**Table S2. Patient-level broad cell type representation.**

|  | **P0** | **P10** | **P11** | **P36** | **P37** | **P38** | **P4** | **P41** | **P43** | **P44** | **P6** | **P8** | **P9** |
| --- | --- | --- | --- | --- | --- | --- | --- | --- | --- | --- | --- | --- | --- |
| **Proliferating** | 4 | 6 | 6 | 10 | 13 | 20 | 37 | 3 | 16 | 12 | 74 | 106 | 23 |
| **B cell** | 5 | 4 | 2 | 0 | 0 | 1 | 18 | 0 | 29 | 1 | 48 | 83 | 25 |
| **Plasma** | 29 | 7 | 80 | 1 | 2 | 2 | 61 | 0 | 32 | 4 | 75 | 71 | 7 |
| **DC** | 43 | 9 | 7 | 10 | 4 | 4 | 100 | 3 | 17 | 24 | 179 | 136 | 33 |
| **Endothelial** | 38 | 43 | 2 | 3 | 5 | 1 | 305 | 0 | 3 | 3 | 32 | 79 | 59 |
| **Mast** | 222 | 3 | 2 | 7 | 0 | 1 | 127 | 3 | 21 | 10 | 144 | 128 | 17 |
| **AT2** | 55 | 16 | 8 | 49 | 0 | 9 | 101 | 3 | 3 | 17 | 345 | 23 | 32 |
| **AT1** | 19 | 16 | 0 | 0 | 0 | 0 | 21 | 0 | 0 | 3 | 1 | 3 | 14 |
| **Club cells** | 10 | 16 | 1 | 9 | 0 | 34 | 59 | 1 | 33 | 0 | 32 | 0 | 39 |
| **T** | 105 | 45 | 16 | 1 | 1 | 0 | 136 | 7 | 14 | 3 | 770 | 339 | 197 |
| **CD8 cytotoxic T** | 109 | 118 | 10 | 0 | 1 | 1 | 213 | 5 | 27 | 10 | 229 | 327 | 185 |
| **NK** | 6 | 0 | 1 | 0 | 0 | 2 | 11 | 0 | 1 | 0 | 115 | 0 | 3 |
| **Fibroblast** | 56 | 6 | 2 | 2 | 257 | 8 | 986 | 1 | 23 | 19 | 57 | 203 | 7 |
| **Macrophage** | 321 | 221 | 74 | 877 | 195 | 418 | 958 | 182 | 37 | 328 | 987 | 506 | 476 |
| **Monocyte** | 316 | 69 | 25 | 66 | 15 | 20 | 731 | 66 | 81 | 132 | 1035 | 63 | 119 |
| **Neutrophil** | 299 | 32 | 62 | 1 | 80 | 0 | 1432 | 17 | 32 | 22 | 862 | 92 | 32 |
