# Supplementary material for: Single-cell and spatial profiling highlights TB-induced myofibroblasts as drivers of lung pathology
Source: J Exp Med. 2026 Jan 5;223(3):e20251067. doi: 10.1084/jem.20251067 (PMC12767585; doi:10.1084/jem.20251067)
Supplement: Table S3 — shows metadata on 4-wk postinfection NHP cohort. [file jem_20251067_tables3.docx]

**Table S3. Metadata on 4-week post-infection NHP cohort.**

| **Monkey number** | **#Uninvolved lung sample** | **#Granuloma sample (published)** |
| --- | --- | --- |
| 13818 | 1 | 5 |
| 13918 | 0 | 5 |
| 13018 | 1 | 0 |
| 13718 | 1 | 0 |
| 13118 | 1 | 0 |
