# Supplementary material for: Single-cell and spatial profiling highlights TB-induced myofibroblasts as drivers of lung pathology
Source: J Exp Med. 2026 Jan 5;223(3):e20251067. doi: 10.1084/jem.20251067 (PMC12767585; doi:10.1084/jem.20251067)
Supplement: Table S4 — shows metadata on M.tb-infected patients used for flow cytometry. [file jem_20251067_tables4.docx]

**Table S4. Metadata on *M.tb* infected patients used for flow cytometry.**

| **Patient** | **Sex** | **Age** | **Previous TB** | **HIV status** | **Pulmonary TB complication** | **Lung tissue collected** |
| --- | --- | --- | --- | --- | --- | --- |
| P388 | Female | 50 | 2021 | Positive | Chronic cough, minor haemoptysis | Left Pneumonectomy |
| P390 | Female | 48 | 2020 | Positive | Massive haemoptysis | Left Pneumonectomy |
| P391 | Male | 45 | 2021 | Positive | Empyema thoracis | Right transempyema Pneumonectomy |
| P392 | Female | 41 | 2020 | Positive | Bronchiectasis, persistant haemoptysis | Right lower lobectomy |
| P393 | Female | 41 | 2015 | Positive | Haemoptysis | Left Pneumonectomy |
