# Supplementary material for: Single-cell and spatial profiling highlights TB-induced myofibroblasts as drivers of lung pathology
Source: J Exp Med. 2026 Jan 5;223(3):e20251067. doi: 10.1084/jem.20251067 (PMC12767585; doi:10.1084/jem.20251067)
Supplement: Table S5 — shows sample count for Visium data. [file jem_20251067_tables5.docx]

**Table S5. Sample count for Visium data**

| TB status | HIV status | Sample count |
| --- | --- | --- |
| current-TB | positive | 10 (3 lymph node or iBALT controls, 7 granuloma) |
| current-TB | negative | 11(5 lymph node or iBALT controls, 6 granuloma) |
| post-TB | positive | 5 (1 iBALT controls, 4 granuloma) |
| post-TB | negative | 4 (1 iBALT controls, 3 granuloma) |

1. M. T. Reichmann, L. B. Tezera, A. F. Vallejo, M. Vukmirovic, R. Xiao, J. Reynolds, S. Jogai, S. Wilson, B. Marshall, M. G. Jones, A. Leslie, J. M. D’Armiento, N. Kaminski, M. E. Polak, P. Elkington, Integrated transcriptomic analysis of human tuberculosis granulomas and a biomimetic model identifies therapeutic targets. *J. Clin. Invest.* **131** (2021), doi:10.1172/JCI148136.

2. S. Raghavan, P. S. Winter, A. W. Navia, H. L. Williams, A. DenAdel, K. E. Lowder, J. Galvez-Reyes, R. L. Kalekar, N. Mulugeta, K. S. Kapner, M. S. Raghavan, A. A. Borah, N. Liu, S. A. Väyrynen, A. D. Costa, R. W. S. Ng, J. Wang, E. K. Hill, D. Y. Ragon, L. K. Brais, A. M. Jaeger, L. F. Spurr, Y. Y. Li, A. D. Cherniack, M. A. Booker, E. F. Cohen, M. Y. Tolstorukov, I. Wakiro, A. Rotem, B. E. Johnson, J. M. McFarland, E. T. Sicinska, T. E. Jacks, R. J. Sullivan, G. I. Shapiro, T. E. Clancy, K. Perez, D. A. Rubinson, K. Ng, J. M. Cleary, L. Crawford, S. R. Manalis, J. A. Nowak, B. M. Wolpin, W. C. Hahn, A. J. Aguirre, A. K. Shalek, Microenvironment drives cell state, plasticity, and drug response in pancreatic cancer. *Cell* **184**, 6119-6137.e26 (2021).
