# Supplementary material for: Single-cell and spatial profiling highlights TB-induced myofibroblasts as drivers of lung pathology
Source: J Exp Med. 2026 Jan 5;223(3):e20251067. doi: 10.1084/jem.20251067 (PMC12767585; doi:10.1084/jem.20251067)
Supplement: Data S1 — shows cell type annotation and epithelial subclustering. [file jem_20251067_datas1.pdf]

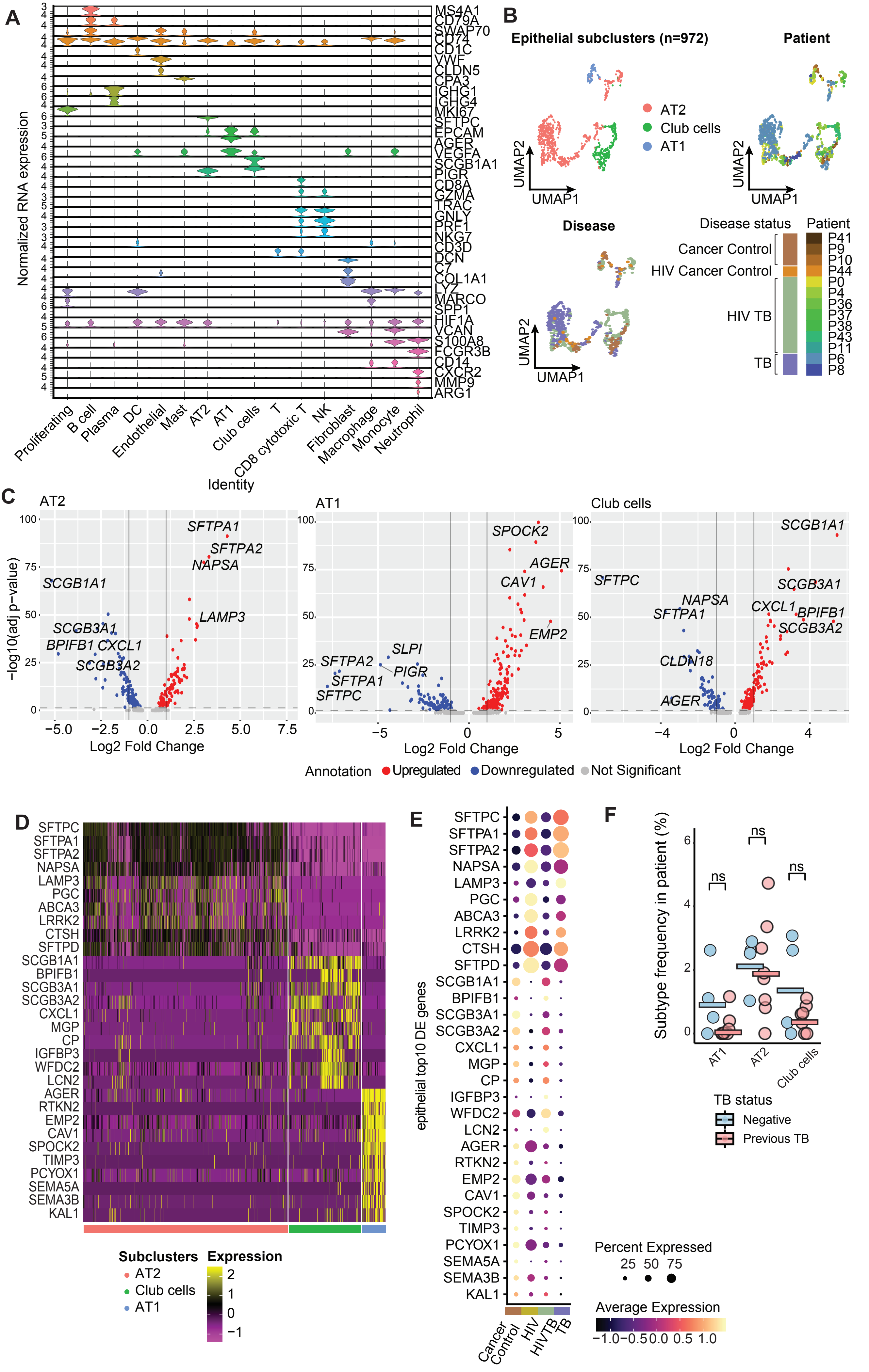
A
Normalized RNA expression
MS4A1
CD79A
SWAP70
CD74
CD1C
VWF
CLDN5
CPA3
IGHG1
IGHG4
MKI67
SFTPC
EPCAM
AGER
VEGFA
SCGB1A1
PIGR
CD8A
GZMA
TRAC
GNLY
PRF1
NKG7
CD3D
DCN
C7
COL1A1
LYZ
MARCO
SPP1
HIF1A
VCAN
S100A8
FCGR3B
CD14
CXCR2
MMP9
ARG1
Proliferating
B cell
Plasma
DC
Endothelial
Mast
AT2
AT1
Club cells
T
CD8 cytotoxic T
NK
Fibroblast
Macrophage
Monocyte
Neutrophil
Identity
B
Epithelial subclusters (n=972)
Patient
AT2
Club cells
AT1
UMAP2
UMAP1
Disease
Disease status
Patient
Cancer Control
HIV Cancer Control
HIV TB
TB
P41
P9
P10
P44
P0
P4
P36
P37
P38
P43
P11
P6
P8
C
AT2
AT1
Club cells
-log10(adj p-value)
Log2 Fold Change
SFTPA1
SFTPA2
NAPSA
SCGB1A1
LAMP3
SCGB3A1
BPIFB1
CXCL1
SCGB3A2
SPOCK2
AGER
CAV1
EMP2
SLPI
PIGR
SFTPA2
SFTPA1
SFTPC
SCGB1A1
SFTPC
SCGB3A1
NAPSA
SFTPA1
CXCL1
BPIFB1
SCGB3A2
CLDN18
AGER
Annotation
Upregulated
Downregulated
Not Significant
D
SFTPC
SFTPA1
SFTPA2
NAPSA
LAMP3
PGC
ABCA3
LRRK2
CTSH
SFTPD
SCGB1A1
BPIFB1
SCGB3A1
SCGB3A2
CXCL1
MGP
CP
IGFBP3
WFDC2
LCN2
AGER
RTKN2
EMP2
CAV1
SPOCK2
TIMP3
PCYOX1
SEMA5A
SEMA3B
KAL1
Subclusters
AT2
Club cells
AT1
Expression
E
epithelial top10 DE genes
Cancer Control
HIV
HIVTB
TB
Percent Expressed
25 50 75
Average Expression
−1.0 −0.5 0.0 0.5 1.0
F
Subtype frequency in patient (%)
ns
ns
ns
AT1
AT2
Club cells
TB status
Negative
Previous TB

Data S1. Cell type annotation and epithelial subclustering. (A) Stacked violin plots (left) for canonical markers for major cell types shown in Fig. 1A. **(B)** Analysis of 972 epithelial cells revealed 3 populations: two alveolar subsets (type 1 and type 2 alveolar cells, also known as AT1 and AT2) and club cells from respiratory bronchioles (top), also colored by patient ID (bottom left) and disease condition (bottom right). **(C)** Volcano plot of differential gene expression results of each epithelial subcluster compared to the rest. Y axis shows -log10 (BH-adjusted *p*-value), X axis shows log2 fold change between cells in subcluster and outside the subcluster. **(D)** Heatmap of subtype top 10 DE genes in each of the epithelial subcluster. **(E)** Expression of marker genes in epithelial cell subclusters by disease conditions. **(F)** Two-sided Fisher's exact test on abundance of detailed epithelial subclusters between TB conditions. Holm's method was applied to p-values to correct for multiple testing. Statistical annotations: p-value > 0.05 (ns).
