# Supplementary material for: Single-cell and spatial profiling highlights TB-induced myofibroblasts as drivers of lung pathology
Source: J Exp Med. 2026 Jan 5;223(3):e20251067. doi: 10.1084/jem.20251067 (PMC12767585; doi:10.1084/jem.20251067)
Supplement: Data S2 — shows expression of marker genes from monocyte/macrophage subclusters and differential abundance testing. [file jem_20251067_datas2.pdf]

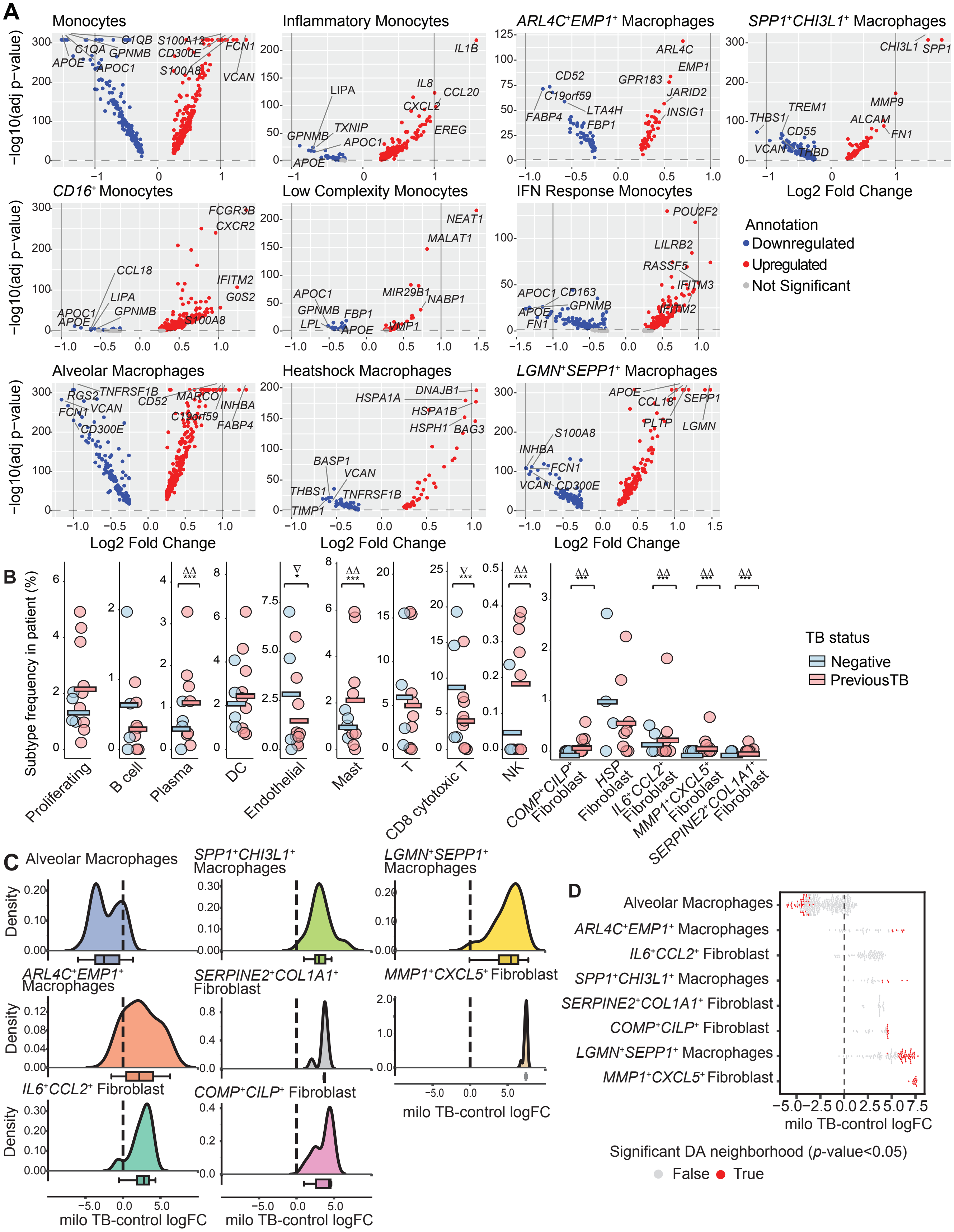

A
Monocytes
Inflammatory Monocytes
ARL4C+EMP1+ Macrophages
SPP1+CHI3L1+ Macrophages
-log10(adj p−value)
C1QB S100A12
C1QA GPNMB CD300E FCN1
APOE APOC1 S100A8
VCAN
IL1B
IL8 CCL20
LIPA
CXCL2
TXNIP EREG
GPNMB APOC1
APOE
ARL4C
EMP1
CD52 GPR183
JARID2
C19orf59
LTA4H INSIG1
FABP4 FBP1
CHI3L1 SPP1
MMP9
TREM1
THBS1 ALCAM
CD55 FN1
VCAN THBD
Log2 Fold Change
CD16+ Monocytes
Low Complexity Monocytes
IFN Response Monocytes
FCGR3B
CXCR2
CCL18
IFITM2
LIPA G0S2
APOC1 GPNMB
APOE S100A8
NEAT1
MALAT1
APOC1 MIR29B1
NABP1
GPNMB FBP1
LPL APOE VMP1
POU2F2
LILRB2
RASSF5
IFITM3
APOC1 CD163
APOE GPNMB IFITM2
FN1
Annotation
Downregulated
Upregulated
Not Significant
Alveolar Macrophages
Heatshock Macrophages
LGMN+SEPP1+ Macrophages
RGS2 TNFRSF1B
CD52 MARCO
FCN1 VCAN INHBA
C19orf59
CD300E FABP4
DNAJB1
HSPA1A
HSPA1B
HSPH1 BAG3
BASP1
VCAN
THBS1 TNFRSF1B
TIMP1
APOE
CCL18 SEPP1
PLTP LGMN
S100A8
INHBA
FCN1
VCAN CD300E
Log2 Fold Change
B
Subtype frequency in patient (%)
Proliferating
B cell
Plasma
DC
Endothelial
Mast
T
CD8 cytotoxic T
NK
COMP+CILP+ Fibroblast
HSP Fibroblast
IL6+CCL2+ Fibroblast
MMP1+CXCL5+ Fibroblast
SERPINE2+COL1A1+ Fibroblast
TB status
Negative
PreviousTB
C
Alveolar Macrophages
SPP1+CHI3L1+ Macrophages
LGMN+SEPP1+ Macrophages
ARL4C+EMP1+ Macrophages
SERPINE2+COL1A1+ Fibroblast
MMP1+CXCL5+ Fibroblast
IL6+CCL2+ Fibroblast
COMP+CILP+ Fibroblast
Density
milo TB-control logFC
D
Alveolar Macrophages
ARL4C+EMP1+ Macrophages
IL6+CCL2+ Fibroblast
SPP1+CHI3L1+ Macrophages
SERPINE2+COL1A1+ Fibroblast
COMP+CILP+ Fibroblast
LGMN+SEPP1+ Macrophages
MMP1+CXCL5+ Fibroblast
milo TB-control logFC
Significant DA neighborhood (p-value<0.05)
False True

Data S2. Expression of marker genes from monocyte/macrophage subclusters and differential abundance testing. (A) Volcano plot of differential gene expression results of each monocyte/macrophage subcluster compared to the rest. Y axis shows -log10 (BH-adjusted *p*-value), X axis shows log2 fold change between cells in subcluster and outside the subcluster. **(B)** Fisher's exact test on abundance of detailed cell subtypes (excluding monocyte/macrophage, neutrophil, epithelial cell) between TB conditions. Statistical annotations: p-value < 0.05 (*), p-value < 0.01 (**), p-value < 0.001 (***), fold-change> 1 (Δ), fold-change >2 (ΔΔ), fold-change <1 (∇). **(C)** Milo differential abundance testing neighborhood log fold change distribution density plot for macrophage and fibroblast subclusters. **(D)** Milo differential abundance testing neighborhood log-fold change and significant results. Neighborhood with differential testing *p*-value <0.05 are colored in red.
