# Supplementary material for: Single-cell and spatial profiling highlights TB-induced myofibroblasts as drivers of lung pathology
Source: J Exp Med. 2026 Jan 5;223(3):e20251067. doi: 10.1084/jem.20251067 (PMC12767585; doi:10.1084/jem.20251067)
Supplement: Data S3 — shows cell–cell interaction analysis by TB conditions. [file jem_20251067_datas3.pdf]

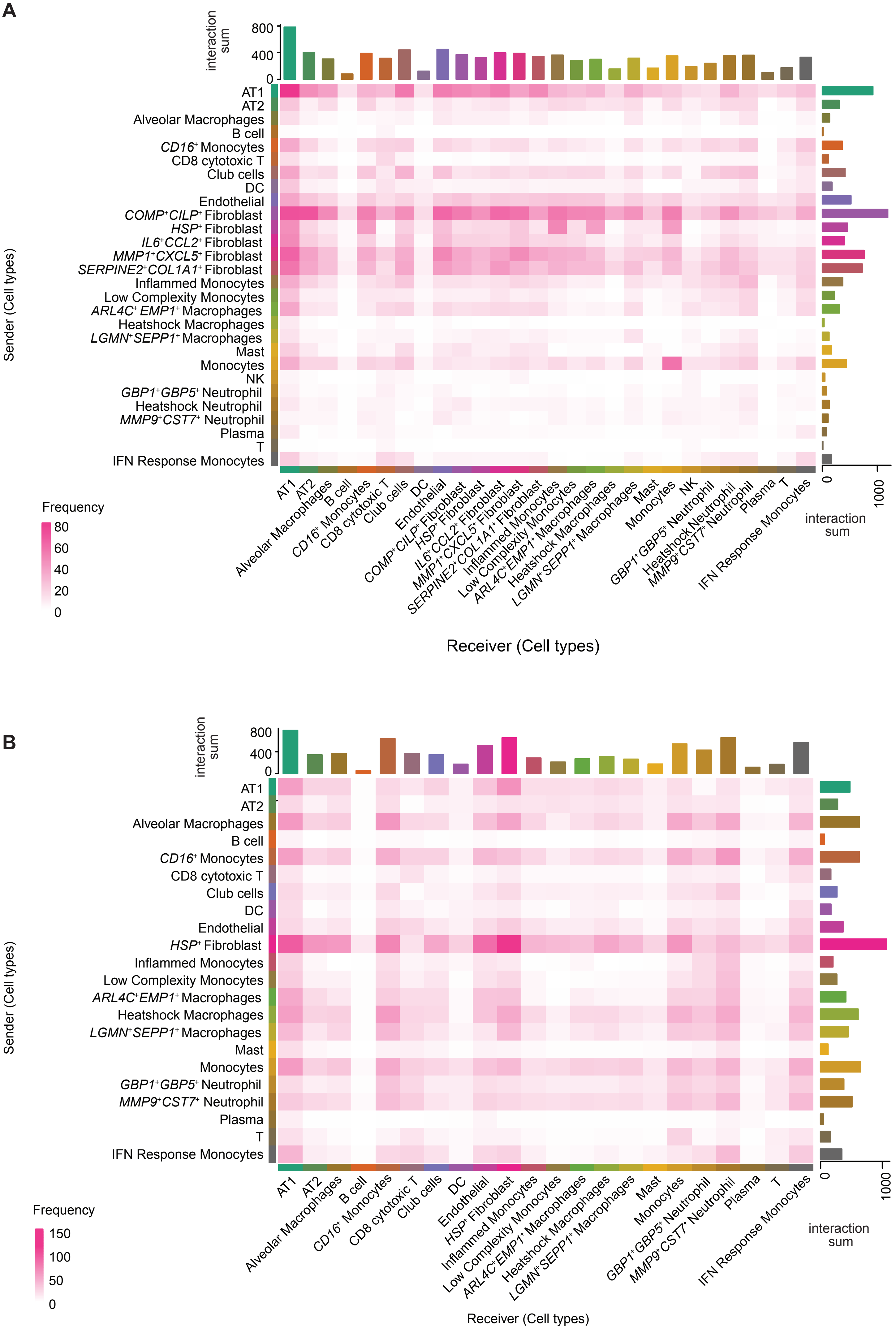
A
interaction sum
800
400
0
AT1
AT2
Alveolar Macrophages
B cell
CD16+ Monocytes
CD8 cytotoxic T
Club cells
DC
Endothelial
COMP+CILP+ Fibroblast
HSP+ Fibroblast
IL6+CCL2+ Fibroblast
MMP1+CXCL5+ Fibroblast
SERPINE2+COL1A1+ Fibroblast
Inflammed Monocytes
Low Complexity Monocytes
ARL4C+ EMP1+ Macrophages
Heatshock Macrophages
LGMN+SEPP1+ Macrophages
Mast
Monocytes
NK
GBP1+GBP5+ Neutrophil
Heatshock Neutrophil
MMP9+CST7+ Neutrophil
Plasma
T
IFN Response Monocytes
Sender (Cell types)
Receiver (Cell types)
Frequency
80
60
40
20
0
0
1000
interaction sum
B
interaction sum
800
400
0
AT1
AT2
Alveolar Macrophages
B cell
CD16+ Monocytes
CD8 cytotoxic T
Club cells
DC
Endothelial
HSP+ Fibroblast
Inflammed Monocytes
Low Complexity Monocytes
ARL4C+EMP1+ Macrophages
Heatshock Macrophages
LGMN+SEPP1+ Macrophages
Mast
Monocytes
GBP1+GBP5+ Neutrophil
MMP9+CST7+ Neutrophil
Plasma
T
IFN Response Monocytes
Sender (Cell types)
Receiver (Cell types)
Frequency
150
100
50
0
0
1000
interaction sum

Data S3. Cell–cell interaction analysis by TB conditions. (A) Heatmap of filtered sender–receiver interaction frequencies from TB-diseased lungs in LIANA analysis. **(B)** Heatmap of filtered sender–receiver interaction frequencies from TB-negative lungs in LIANA.
