# Supplementary material for: Single-cell and spatial profiling highlights TB-induced myofibroblasts as drivers of lung pathology
Source: J Exp Med. 2026 Jan 5;223(3):e20251067. doi: 10.1084/jem.20251067 (PMC12767585; doi:10.1084/jem.20251067)
Supplement: Data S4 — shows cell–cell interaction changes between TB conditions and AT1 cell sender activities. [file jem_20251067_datas4.pdf]

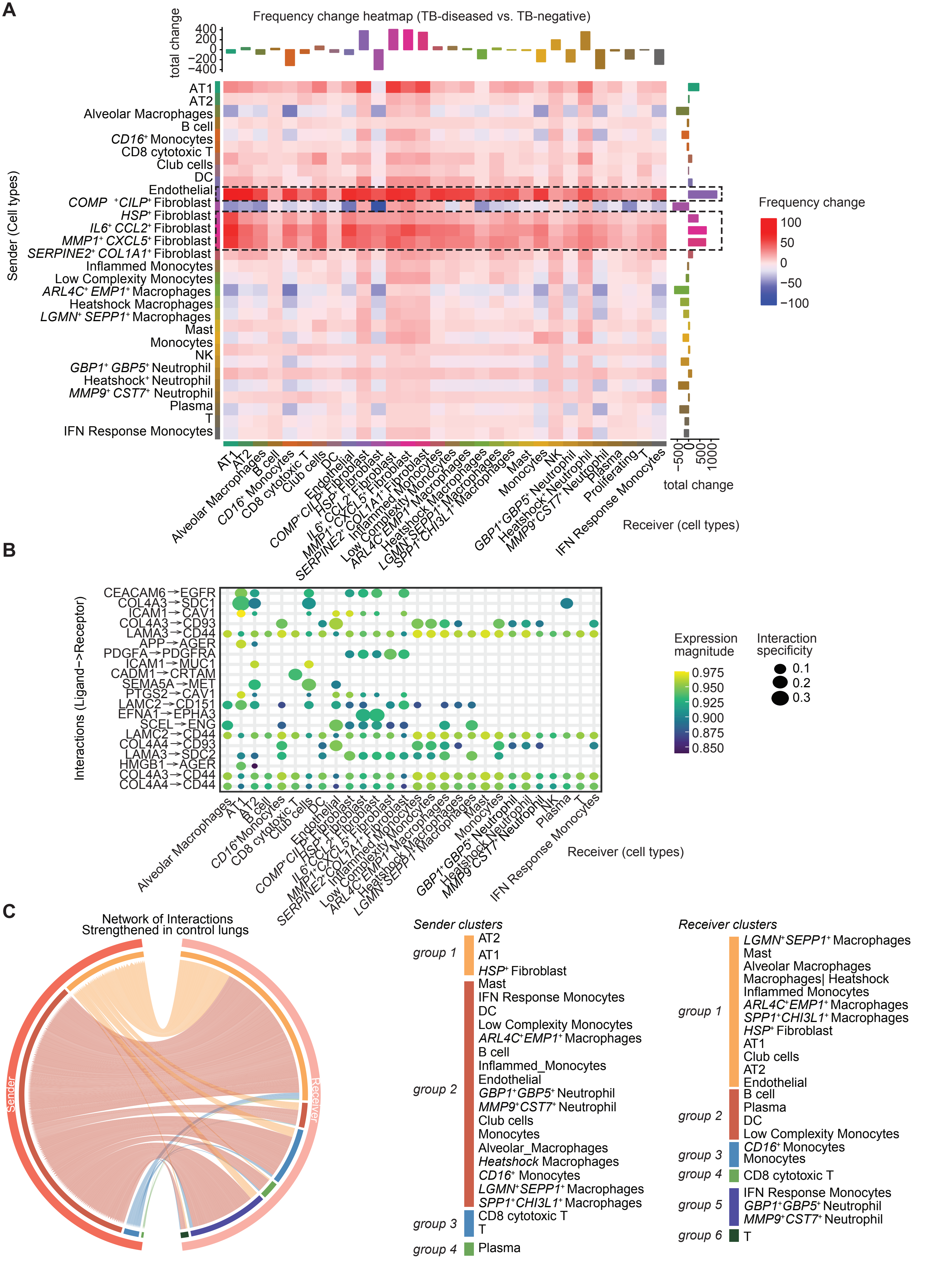
A
Frequency change heatmap (TB-diseased vs. TB-negative)
total change
400
200
0
−200
−400
AT1
AT2
Alveolar Macrophages
B cell
CD16⁺ Monocytes
CD8 cytotoxic T
Club cells
DC
Endothelial
COMP⁺CILP⁺ Fibroblast
HSP⁺ Fibroblast
IL6⁺ CCL2⁺ Fibroblast
MMP1⁺ CXCL5⁺ Fibroblast
SERPINE2⁺ COL1A1⁺ Fibroblast
Inflammed Monocytes
Low Complexity Monocytes
ARL4C⁺ EMP1⁺ Macrophages
Heatshock Macrophages
LGMN⁺ SEPP1⁺ Macrophages
Mast
Monocytes
NK
GBP1⁺ GBP5⁺ Neutrophil
Heatshock⁺ Neutrophil
MMP9⁺ CST7⁺ Neutrophil
Plasma
T
IFN Response Monocytes
Sender (Cell types)
SPP1⁺CHI3L1⁺ Macrophages
Proliferating
Receiver (cell types)
−500
0
500
1000
total change
Frequency change
100
50
0
−50
−100
B
CEACAM6→EGFR
COL4A3→SDC1
ICAM1→CAV1
COL4A3→CD93
LAMA3→CD44
APP→AGER
PDGFA→PDGFRA
ICAM1→MUC1
CADM1→CRTAM
SEMA5A→MET
PTGS2→CAV1
LAMC2→CD151
EFNA1→EPHA3
SCEL→ENG
LAMC2→CD44
COL4A4→CD93
LAMA3→SDC2
HMGB1→AGER
COL4A3→CD44
COL4A4→CD44
Interactions (Ligand->Receptor)
Receiver (cell types)
Expression magnitude
0.975
0.950
0.925
0.900
0.875
0.850
Interaction specificity
0.1
0.2
0.3
C
Network of Interactions
Strengthened in control lungs
Sender
Receiver
Sender clusters
group 1
AT2
AT1
HSP⁺ Fibroblast
group 2
Mast
IFN Response Monocytes
DC
Low Complexity Monocytes
ARL4C⁺EMP1⁺ Macrophages
B cell
Inflammed_Monocytes
Endothelial
GBP1⁺GBP5⁺ Neutrophil
MMP9⁺CST7⁺ Neutrophil
Club cells
Monocytes
Alveolar_Macrophages
Heatshock Macrophages
CD16⁺ Monocytes
LGMN⁺SEPP1⁺ Macrophages
SPP1⁺CHI3L1⁺ Macrophages
group 3
CD8 cytotoxic T
T
group 4
Plasma
Receiver clusters
group 1
LGMN⁺SEPP1⁺ Macrophages
Mast
Alveolar Macrophages
Macrophages| Heatshock
Inflammed Monocytes
ARL4C⁺EMP1⁺ Macrophages
SPP1⁺CHI3L1⁺ Macrophages
HSP⁺ Fibroblast
AT1
Club cells
AT2
Endothelial
group 2
B cell
Plasma
DC
Low Complexity Monocytes
group 3
CD16⁺ Monocytes
Monocytes
group 4
CD8 cytotoxic T
group 5
IFN Response Monocytes
GBP1⁺GBP5⁺ Neutrophil
MMP9⁺CST7⁺ Neutrophil
group 6
T

Data S4. Cell–cell interaction changes between TB conditions and AT1 cell sender activities. (A) Differential analysis on LIANA results on detailed subtypes. Differences in frequencies are normalized to maximum of absolute differences across all cell pairs. **(B)** Top 20 interactions from AT1 cells in TB-diseased lung from LIANA analysis. **(C)** Circos plots of significant interaction pairs in TB-negative lungs from LIANA where sender and receiver cell types in each condition are clustered to reflect similar patterns.
