# Supplementary material for: Single-cell and spatial profiling highlights TB-induced myofibroblasts as drivers of lung pathology
Source: J Exp Med. 2026 Jan 5;223(3):e20251067. doi: 10.1084/jem.20251067 (PMC12767585; doi:10.1084/jem.20251067)
Supplement: Data S5 — shows expression of hypothesized secreted ligands by MMP1+CXCL5+ fibroblast in human TB LN granuloma vs. healthy LN. [file jem_20251067_datas5.pdf]

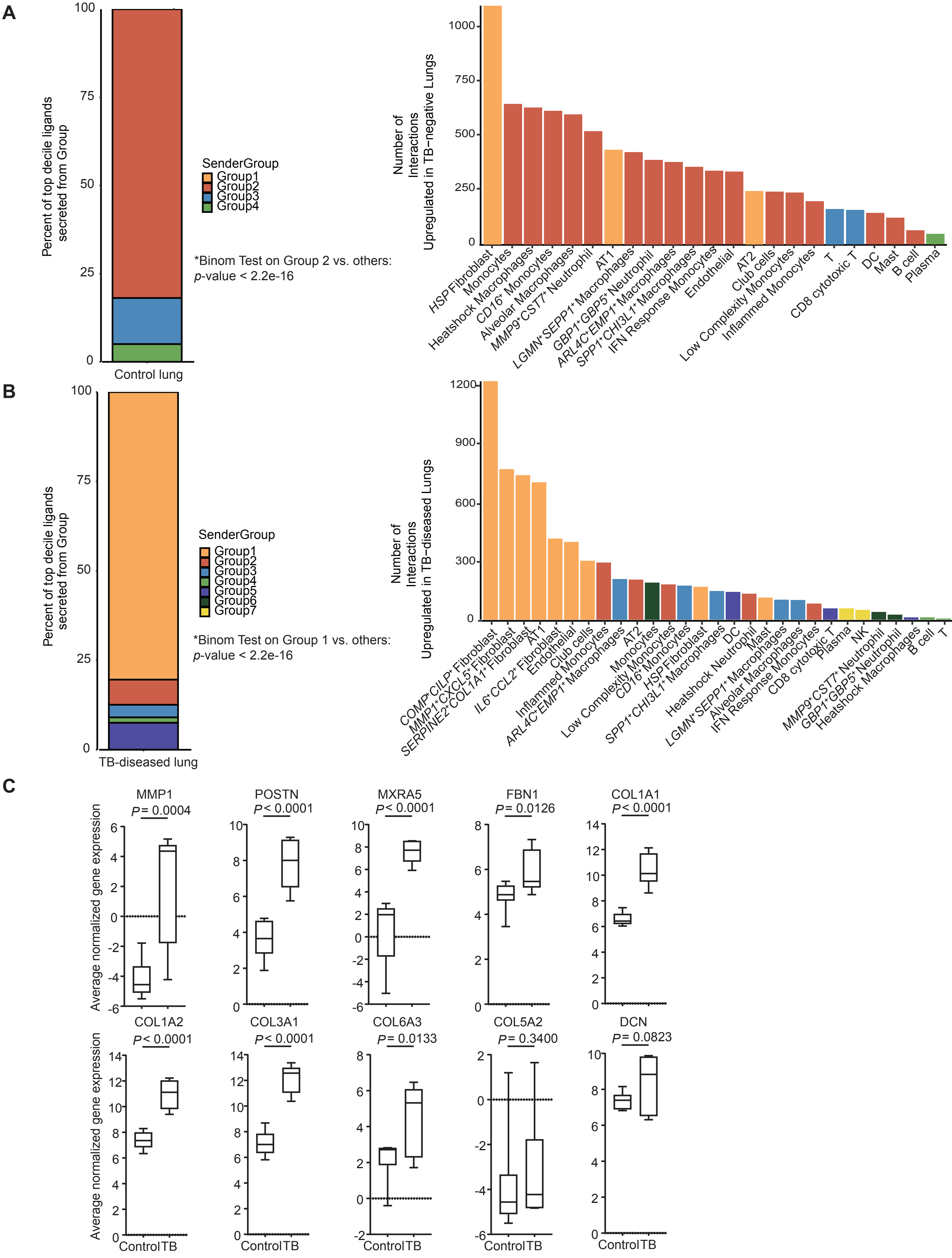

A
Percent of top decile ligands secreted from Group
Control lung
SenderGroup
Group1
Group2
Group3
Group4
*Binom Test on Group 2 vs. others:
p-value < 2.2e-16
Number of Interactions Upregulated in TB−negative Lungs
HSP Fibroblast
Monocytes
Heatshock Macrophages
CD16+ Monocytes
Alveolar Macrophages
MMP9+CST7+ Neutrophil
AT1
LGMN+SEPP1+ Macrophages
GBP1+GBP5+ Neutrophil
ARL4C+EMP1+ Macrophages
SPP1+CHI3L1+ Macrophages
IFN Response Monocytes
Endothelial
AT2
Club cells
Low Complexity Monocytes
Inflammed Monocytes
T
CD8 cytotoxic T
DC
Mast
B cell
Plasma
B
Percent of top decile ligands secreted from Group
TB-diseased lung
SenderGroup
Group1
Group2
Group3
Group4
Group5
Group6
Group7
*Binom Test on Group 1 vs. others:
p-value < 2.2e-16
Number of Interactions Upregulated in TB−diseased Lungs
COMP+CILP+ Fibroblast
MMP1+CXCL5+ Fibroblast
SERPINE2+COL1A1+ Fibroblast
AT1
IL6+CCL2+ Fibroblast
Endothelial
Club cells
Inflammed Monocytes
ARL4C+EMP1+ Macrophages
AT2
Monocytes
Low Complexity Monocytes
CD16+ Monocytes
HSP Fibroblast
SPP1+CHI3L1+ Macrophages
DC
Heatshock Neutrophil
Mast
LGMN+SEPP1+ Macrophages
Alveolar Macrophages
IFN Response Monocytes
CD8 cytotoxic T
Plasma
NK
MMP9+CST7+ Neutrophil
GBP1+GBP5+ Neutrophil
Heatshock Macrophages
B cell
T
C
Average normalized gene expression
MMP1 P = 0.0004
POSTN P < 0.0001
MXRA5 P < 0.0001
FBN1 P = 0.0126
COL1A1 P < 0.0001
COL1A2 P < 0.0001
COL3A1 P < 0.0001
COL6A3 P = 0.0133
COL5A2 P = 0.3400
DCN P = 0.0823
Control TB

Data S5. Expression of hypothesized secreted ligands by MMP1^{+}CXCL5^{+} fibroblast in human TB lymph node granuloma vs. healthy lymph node. (A) Left: Proportion of top 10% ligands secreted in TB-negative lungs by sender cluster. Right: Number of upregulated interactions by sender subcluster in TB-negative lungs. **(B)** Left: Proportion of top 10% ligands secreted in TB-diseased lungs by sender cluster. Right: Number of upregulated interactions by sender subcluster in TB-diseased lungs. **(C)** Evaluation of LIANA imputed ligand expression in bulk lymph node TB granuloma dataset from Reichmann et al. (2021). COL1A1, COL1A2, COL3A1, COL5A2, COL6A3, POSTN, FBN1, MXRA5, DCN are all suggested by LIANA analysis as ligands secreted by the *MMP1^{+} CXCL5^{+}* fibroblast population. Average gene expression of 7 control and 7 TB lymph nodes are shown, with one-tailed unpaired T test *p*-value result shown on top.

Reference

Reichmann, M.T., L.B. Tezera, A.F. Vallejo, M. Vukmirovic, R. Xiao, J. Reynolds, S. Jogai, S. Wilson, B. Marshall, M.G. Jones, A. Leslie, J.M. D'Armiento, N. Kaminski, M.E. Polak, and P. Elkington. 2021. Integrated transcriptomic analysis of human tuberculosis granulomas and a biomimetic model identifies therapeutic targets. *J. Clin. Invest.* 131:e148136. https://doi.org/10.1172/JCI148136
