# Supplementary material for: Single-cell and spatial profiling highlights TB-induced myofibroblasts as drivers of lung pathology
Source: J Exp Med. 2026 Jan 5;223(3):e20251067. doi: 10.1084/jem.20251067 (PMC12767585; doi:10.1084/jem.20251067)
Supplement: Data S6 — shows spatial transcriptomics analysis on post- and current TB lung resections. [file jem_20251067_datas6.pdf]

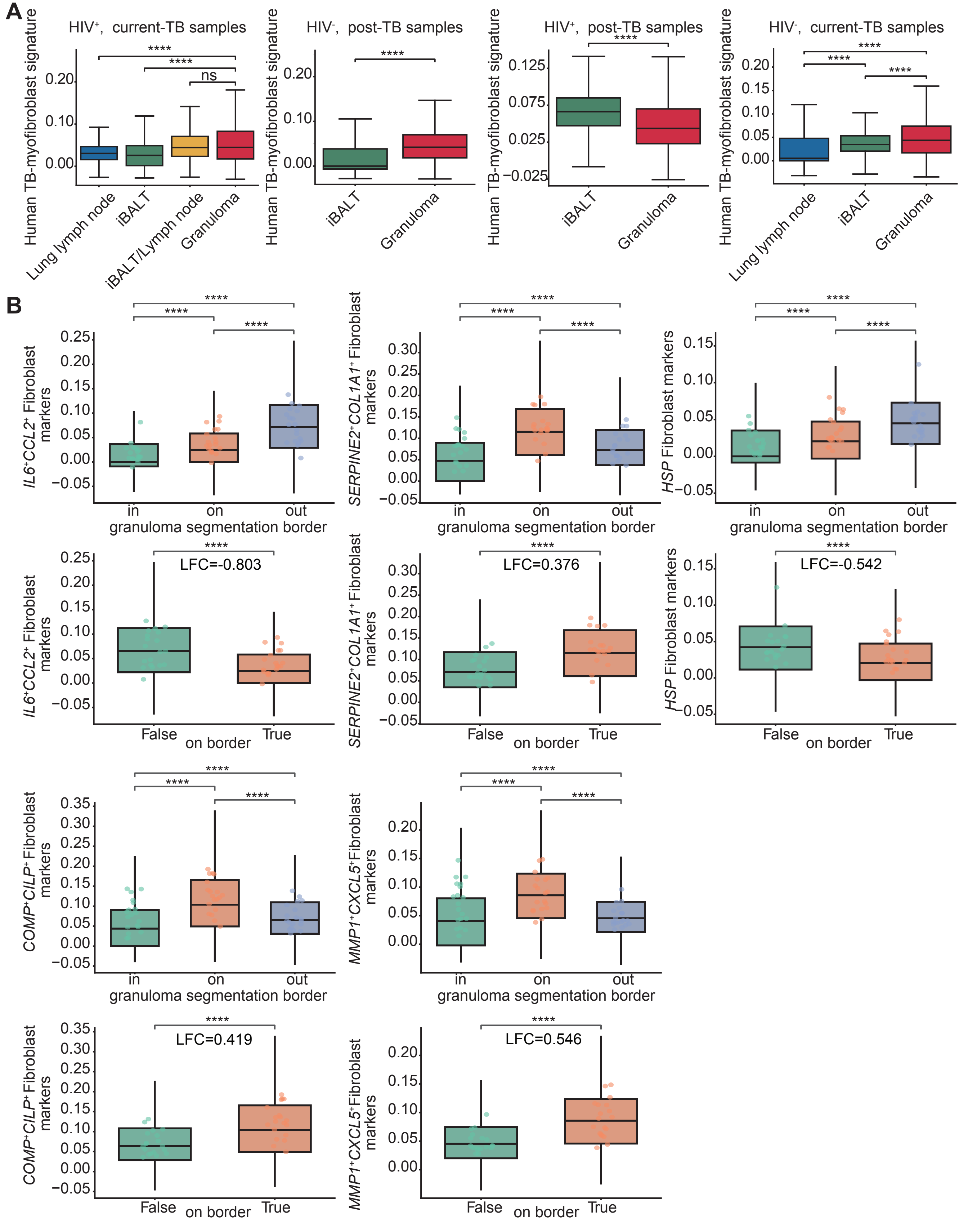
A
HIV+, current-TB samples
HIV-, post-TB samples
HIV+, post-TB samples
HIV-, current-TB samples
Human TB-myofibroblast signature
Lung lymph node
iBALT
iBALT/Lymph node
Granuloma
ns
****
B
IL6+CCL2+ Fibroblast markers
SERPINE2+COL1A1+ Fibroblast markers
HSP Fibroblast markers
COMP+CILP+ Fibroblast markers
MMP1+CXCL5+ Fibroblast markers
in
on
out
granuloma segmentation border
False
True
on border
LFC=-0.803
LFC=0.376
LFC=-0.542
LFC=0.419
LFC=0.546

Data S6. Spatial transcriptomics analysis on post- and current-TB lung resections. (A) Distribution of human TB-myofibroblast signature score by tissue type in each disease condition of Visium cohort. The plotted values are the module score on each Visium spot. iBALT: induced bronchus-associated lymphoid tissue. Two-sided Mann-Whitney U test without correction was used for statistical testing. Statistical annotations: *p*-value < 0.0001 (****), *p*-value>0.05 (ns). **(B)** Distribution of fibroblast subtype marker (top 50 DE genes) module scores on Visium data by segmented regions with respect to granuloma structures. Two-sided Mann-Whitney U test without correction was used for statistical testing. Statistical annotations: *p*-value < 0.0001 (****). Log fold-change (LFC) shown are computed between spots on granuloma segmentation border and the rest.
